# Supplementary material for: Genome-wide CRISPR screens identify novel regulators of wild-type and mutant p53 stability
Source: Mol Syst Biol. 2024 Apr 5;20(6):719–40. doi: 10.1038/s44320-024-00032-x (PMC11148184; doi:10.1038/s44320-024-00032-x)
Supplement: Supplementary file 1 — Appendix [file 44320_2024_32_MOESM1_ESM.pdf]

APPENDIX FIGURES

Genome-wide CRISPR screens identify novel regulators of wild-type and mutant p53 stability

YiQing Lü[1, 2, 8], Tiffany Cho[1,2], Saptaparna Mukherjee[3], Carmen Florencia Suarez[4], Nicolas S. Gonzalez-Foutel[4], Ahmad Malik[1,2], Sebastien Martinez[1], Dzana Dervovic[1], Robin Hyunseo Oh[1,2], Ellen Langille[1, 2], Khalid N. Al-Zahrani[1], Lisa Hoeg[1], Zhen Yuan Lin[1], Ricky Tsai[1], Geraldine Mbamalu[1], Varda Rotter[3], Patricia Ashton-Prolla[5], Jason Moffat[2,6,7], Lucia B. Chemes[4], Anne-Claude Gingras[1,2], Moshe Oren[3], Daniel Durocher[1,2] and Daniel Schramek[1,2 *]

[1] Centre for Molecular and Systems Biology, Lunenfeld-Tanenbaum Research Institute, Mount Sinai Hospital, Toronto, Ontario, M5G 1X5, Canada

[2] Department of Molecular Genetics, University of Toronto, Toronto, Ontario, M5S 1A8, Canada

[3] Department of Molecular Cell Biology, Weizmann Institute of Science, Rehovot, Israel

[4] Instituto de Investigaciones Biotecnológicas (IIBiO-CONICET), Universidad Nacional de San Martín, Buenos Aires, Argentina

[5] Departamento de Genética, UFRGS and Serviço de Genetica Médica HCPA, Porto Alegre, Brasil

[6] Institute of Biomedical Engineering, University of Toronto, Toronto, Ontario, M5S3G9, Canada

[7]Genetics and Genome Biology Program, Hospital for Sick Children, Toronto, Ontario, M5G 1X8, Canada

[8] current affiliation: Department of Biology, Suffolk University, Boston, Massachusetts, 02108, USA

* Correspondence and requests for materials should be addressed to Daniel Schramek

Daniel Schramek, PhD
Lunenfeld-Tanenbaum Research Institute
Mount Sinai Hospital
Toronto, Ontario, Canada M5G 1X5
Phone: +1 416 586-4800
Fax: +1 416 586-8869
schramek@lunenfeld.ca

Table of Contents

Appendix Figure S1 page 2
Appendix Figure S2 page 3
Appendix Figure S3 page 4
Appendix Figure S4 page 6
Appendix Figure S5 page 8
Appendix Figure S6 page 9
Appendix Figure S7 page 10

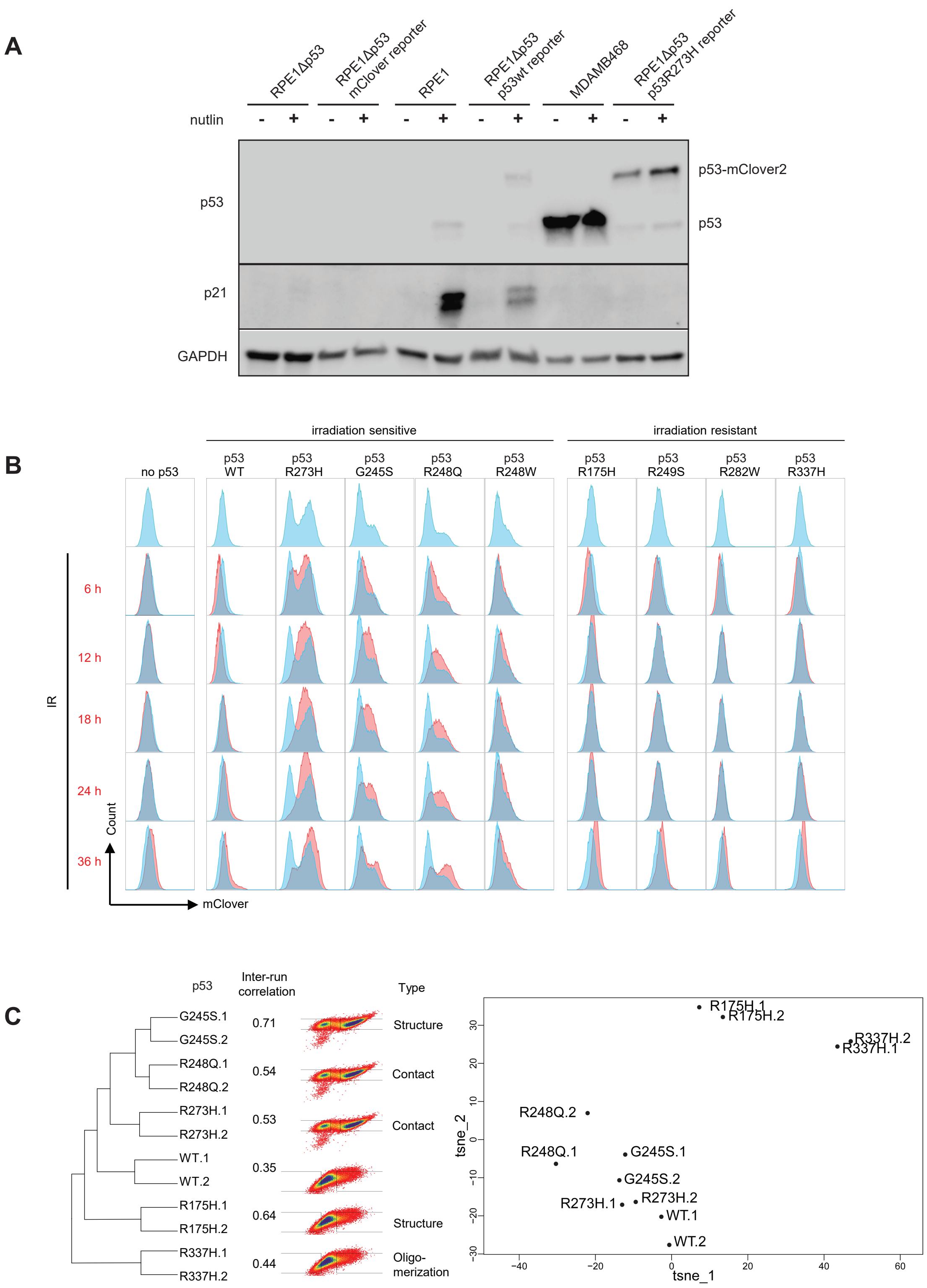

Appendix Figure S1. CRISPR screen for regulators of p53 stability.
A. Representative Western Blot comparing the protein levels of p53 and p21 in RPE1 reporter lines (empty reporter, wild-type p53, and p53R273H) and in parental RPE1 as well as p53-knockout RPE1 cell lines carrying endogenous wild-type p53 and in MB-MDA-468 cell line carrying mutant R273H p53 treated with Nutlin 3a or untreated. GAPDH served as a loading control. Results are reproducible over biological triplicates.
B. Flow cytometry blots depicting the dynamic levels of each p53 mutant after x-irradiation (1 Gy) over time. Results are reproducible over biological triplicates.
C. Unsupervised hierarchy clustering of each screen replicate (left) and visualized as t-distributed stochastic neighbor embedding (t-SNE) blot (right). The inter-run correlation of each screen is computed by comparing the normZ scores of each gene from the two replicates.

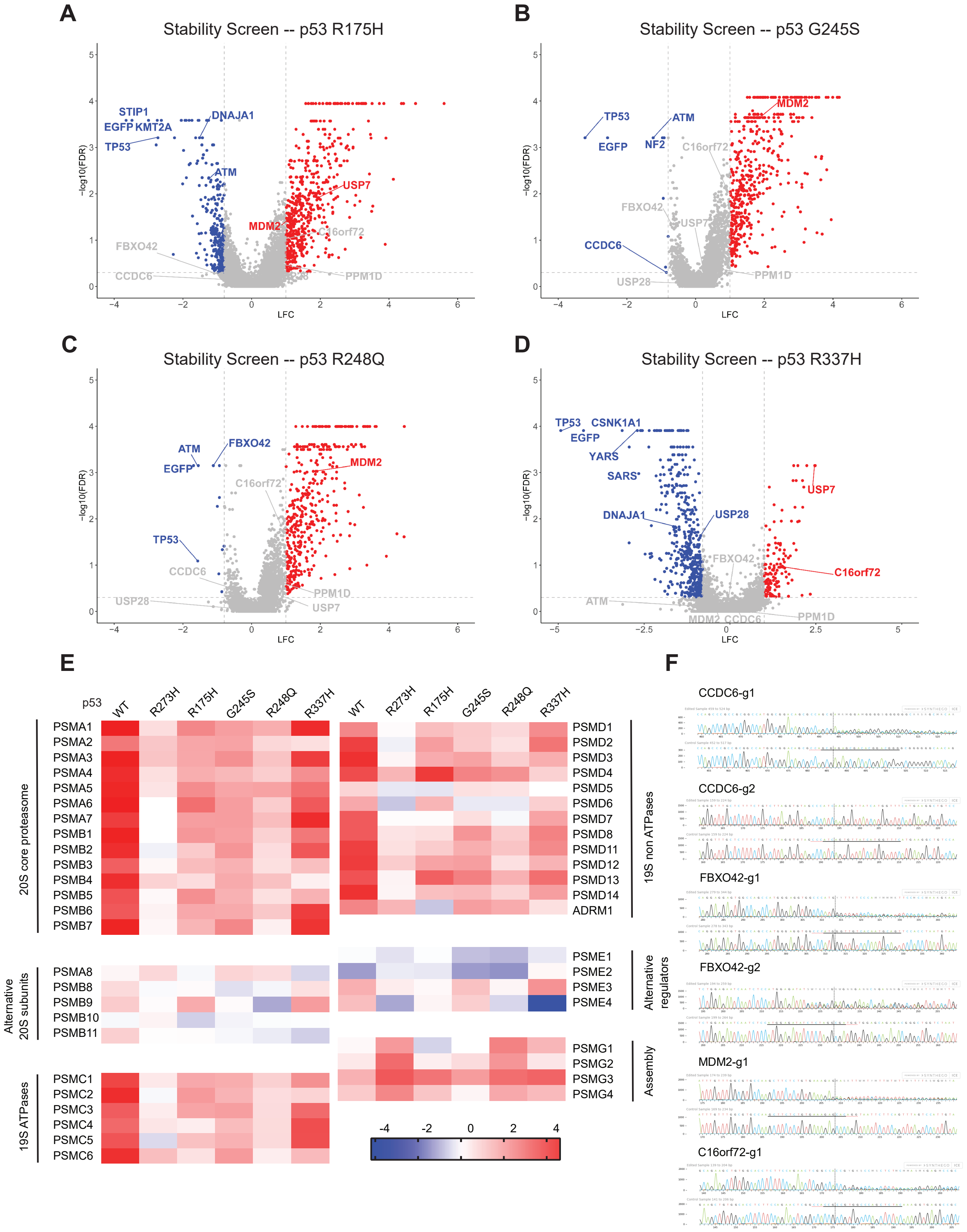

Appendix Figure S2. CRISPR screen for regulators of p53 stability.
A-D. Screen results for R175H, G245, R248Q, and R337H p53 stability reporter screens.
E. Comparison of different p53 mutants and the effects on their stabilities (normZ scores) upon CRISPR/Cas9-mediated mutagenesis of individual members of the proteasome subunits. A positive normZ (red) indicates that genetic ablation of a gene leads to increased p53 protein stability, and negative normZ (blue) indicates decreased p53 stability.
F. Guides efficiency measured as genetic indel abundances of sgRNAs targeting *CCDC6, FBXO42, MDM2, and C16orf72* in the clonal RPE1-reporters and PANC-1 lines, using Inference of CRISPR Edits (ICE) analysis. All guides used achieved >50% knockout (indel%) efficiency.

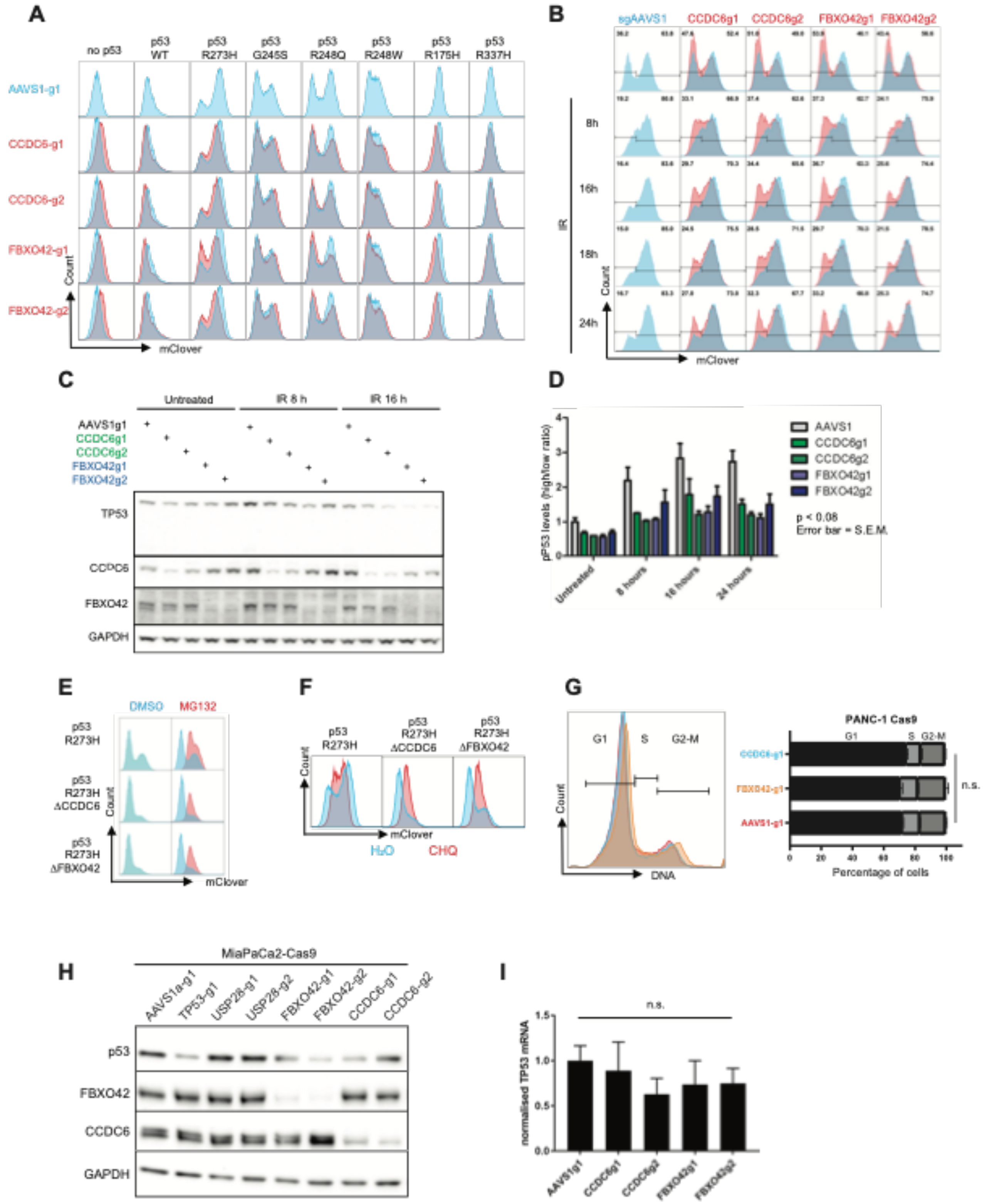

Appendix Figure S3. FBXO42-CCDC6 axis regulates p53 stabilities across wild-type and different mutants.

A. Flow cytometry blots depicting the levels of indicated p53 mutants upon depletion of CCDC6 and FBXO42 measured 10 days post-transduction. Results are reproducible over biological triplicates.

B. Flow cytometry blots depicting the levels of p53 R273H mutant upon depletion of CCDC6 or FBXO42 and after x-irradiation (0.5 Gy) over time. The percentage of p53-mClover low and high cells is displayed. Results are reproducible over biological triplicates.

C. Western blot analysis of endogenous p53 R273H protein levels in PANC-1-Cas9 cells upon depletion of *CCDC6* or *FBXO42* measured 10 days post-transduction, and at various time points after irradiation. Results are reproducible over biological triplicates.

D. Quantification of p53 R273H protein levels in PANC-1-Cas9 cells upon depletion of *CCDC6,* or *FBXO42,* and at various time points after irradiation, measured using a flow cytometer at 10 days post-transduction. The ratios of the bimodal populations (p53-mClover high vs low) from all time points were normalized to the untreated sgAAVS1 control group. (*: $p<0.08$, error bar indicates the standard deviation of the mean). Results are reproducible over biological triplicates. Two tailed unpaired t-test was used for statistical analysis.
E. Flow cytometry blots depicting the $p53^{R273H}$-mClover levels upon depletion of *CCDC6* or *FBXO42* and treatment with the proteasomal inhibitor MG132 (10 µM; 12 hours). Results are reproducible over biological triplicates.
F. Flow cytometry blots depicting the $p53^{R273H}$-mClover levels upon depletion of *CCDC6* or *FBXO42* and treatment with the lysosome inhibitor chloroquine (10 µM; 30 hours). Results are reproducible over biological triplicates.
G. Flow cytometry blots depicting the cell cycle profile (left) of PANC-1 Cas9 cells that have been depleted of FBXO42 or CCDC6 compared to AAVS1 control. The cell cycle profile is further quantified in the bar graph (right) (n.s. $p > 0.25$). Results are reproducible over biological triplicates.
H. Western blot analysis of endogenous p53 R248W protein levels in MiaPaCa2-Cas9 cells upon depletion of *TP53, USP28, CCDC6* or *FBXO42* compared to *AAVS1* control. Results are reproducible over biological triplicates.
I. Quantitative real-time PCR analysis of the PANC-1 cells with depletion of *CCDC6* or *FBXO42*. The *TP53* transcript level was normalised to the housekeeping gene Peptidyl-prolyl cis-trans isomerase B (*PPIB*). The experiment was performed in biological triplicates. Results are analyzed from biological triplicates, and two-tailed unpaired t-test was used for statistical analysis.

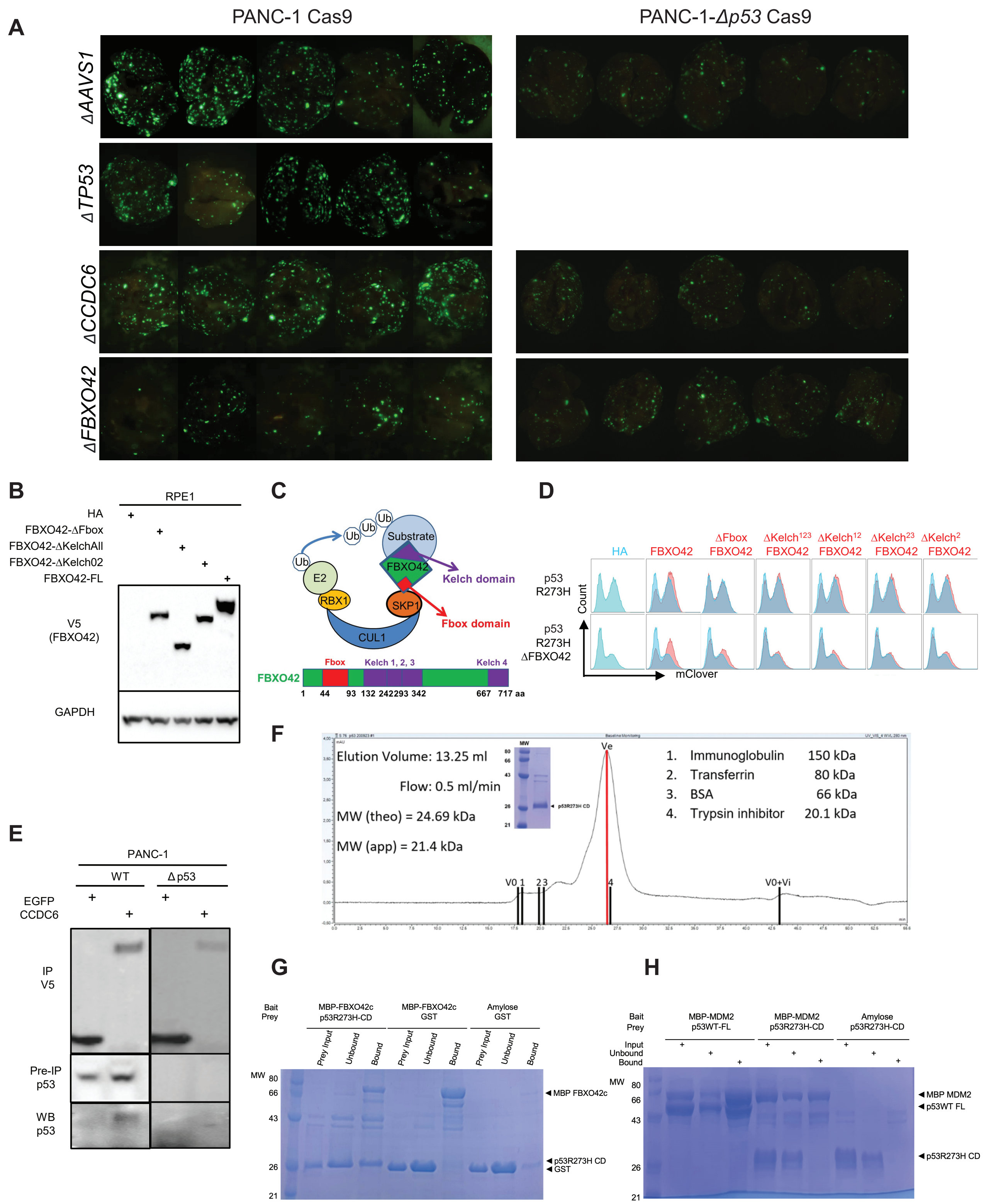

Appendix Figure S4. Regulation of p53 stability by FBXO42-CCDC6.
A. The metastatic colonization of the lung by PANC-1-Cas9-GFP cells and p53-null PANC-1-Δp53-Cas9-GFP cells with the indicated genotype was assessed. Whole lung green-fluorescence images are shown. (n=5 for each condition.)
B. Western blot showing the expression levels of the FBXO42 full-length protein and the indicated FBXO42 truncation mutants. Results are reproducible over biological triplicates.
C. Schematic of FBXO42 and its functional domains and interaction partners.
D. Flow cytometry blots depicting the $p53^{R273H}$-mClover levels upon depletion of *FBXO42* and ectopic expression of various FBXO42 truncation mutants measured at 7 days post-transduction. The truncation domains are illustrated by the schematics in C. Results are reproducible over biological triplicates.

E. Co-immunoprecipitation (co-IP) of CCDC6 and p53 R273H in PANC-1 cells. Lysates of PANC-1 cells overexpressing V5-tagged CCDC6 or an EGFP control construct with or without genetic ablation of the endogenous p53 R273H gene were immunoprecipitated using a V5-specific antibody, followed by Western blot analysis of endogenous p53. Results are reproducible over biological triplicates.
F. Recombinantly expressed p53-CD-R273H is mainly monomeric as assessed by Size Exclusion Chromatography (SEC). The p53-CD-R273H construct elutes in a single major peak from a Superdex-75 column, with a Ve of 13.25 ml at a flow rate of 0.5 ml/min. The calculated MW (21.4 KDa) is in good agreement with the theoretical MW of a p53CD monomer (24.7 KDa). No detectable protein oligomerization was observed, as revealed by no peak in the column Vo. All runs were carried out in a 20 mM sodium phosphate buffer, with 2 mM DTT, 200 mM NaCl, pH 7.00. 100 μL loop injections of p53-CD-R273H were performed, p53-CD-R273H stock was used at a concentration of 2.88 mg/ml (115.2 μM). Markers used were: Acetone 2% (Vo+Vi), Blue dextran 1 mg/ml (Vo), Immunoglobulin 2.5 mg/ml, Transferrin 1 mg/ml, BSA 5 mg/ml and Trypsin inhibitor 3 mg/ml.
G. *In vitro* binding assay of the core domain of p53-R273H (p53CD-R273H) with MBP-FBXO42c. The MBP-tagged Kelch domain of FBXO42c (FBXO42c, aa 105-360) and the core DNA-binding domain of p53 R273H (p53CD-R273H, aa 90-311) were recombinantly expressed and purified from the Bl21DE3 *E. coli* strain. MBP-FBXO42c coupling was pre-coupled to amylose resin. Following incubation of MBP-FBXO42c with p53CD-R273H, amylose resin-coupled MBP-FBXO42c was able to capture p53CD-R273H and a fraction of p53CD-R273H was found in the Bound fraction. As a specificity control, amylose-coupled MBP-FBXO42c was unable to bind to the unrelated protein GST, which remained in the unbound fraction. A second control was performed to show that GST does not bind to the amylose resin. In this control, GST was found in the unbound fraction. The input, unbound and bound fractions were resolved on SDS-PAGE and stained with Coomassie blue.
H. Specificity controls demonstrate *in vitro* binding of MDM2 to p53-full length-WT (p53-FL-WT) but not to p53CD-R273H. MBP-MDM2, p53-FL-WT and p53CD-R273H were recombinantly expressed and purified from the Bl21DE3 *E. coli* strain. MBP-MDM2 was pre-coupled to amylose resin. Following incubation of MBP-MDM2 and p53-FL-WT, amylose-resin coupled MBP-MDM2 was able to capture p53-FL-WT and p53-FL-WT was found mainly in the bound fraction. The results are in agreement with the main binding site for MDM2, which is located in the p53 N-terminal transactivation domain. However, after incubation of MBP-MDM2 with P53CD-R273H, amylose-coupled MBP-MDM2 was unable to capture p53-CD-R273H, which remained fully in the unbound fraction. The results are expected since the main MDM2 binding site is not present in the p53 core domain region. An additional control was performed to show that p53-CD-R273H was unable to bind to amylose resin. The input, unbound and bound fractions were resolved on SDS-PAGE and stained with Coomassie blue.

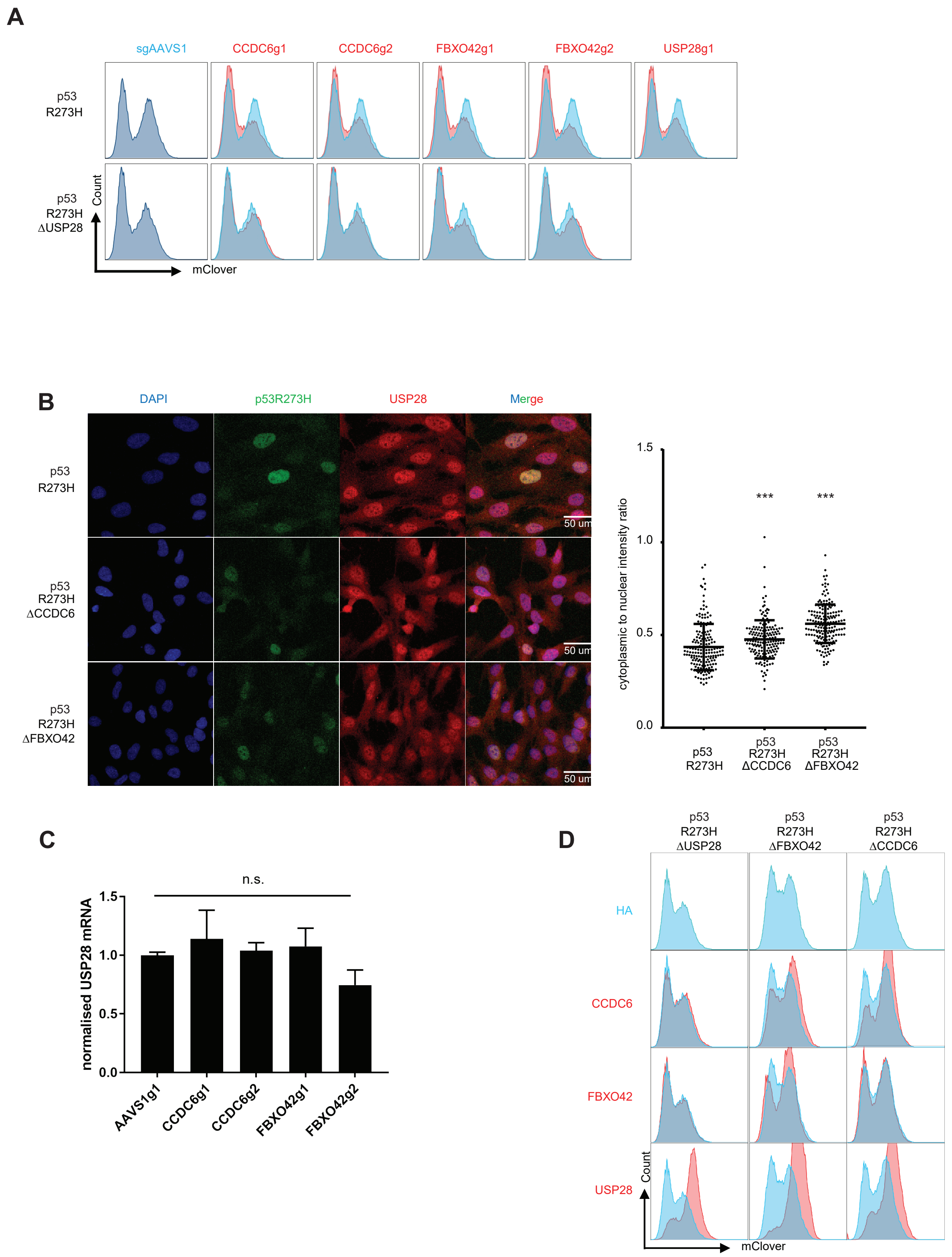

Appendix Figure S5. Mapping the genetic interaction network of FBXO42-CCDC6 and mutant p53.
A. Flow cytometry blots depicting the p53^{R273H}-mClover levels upon depletion of *CCDC6* or *FBXO42* or *USP28* or upon concomitant depletion of *USP28* and *CCDC6* or concomitant depletion of *USP28* and *FBXO42*. Results are reproducible over biological triplicates.
B. Immunofluorescence imaging showing localization and levels of R273H p53-mClover (FITC) and USP28 (Alexa 568) in RPE1 p53^{R273H} -mClover cells with depletion of *CCDC6* or *FBXO42*. The ratio of cytoplasmic to nuclear intensities were quantified (n>200). Error bar = S.E.M. *** p<0.01.
C. Quantitative real-time PCR analysis of the PANC-1 cells with depletion of *CCDC6* or *FBXO42*. The *USP28* transcript level was normalized to *PPIB*. Experiment performed in biological triplicates.
D. Flow cytometry blots depicting p53^{R273H} -mClover levels in p53 R273H-reporter RPE1 cells with clonal depletions of *USP28* (left), *FBXO42* (middle) or *CCDC6* (right), upon ectopic expression of CCDC6 FBXO42 or USP28, measured at 7 days post-transduction.

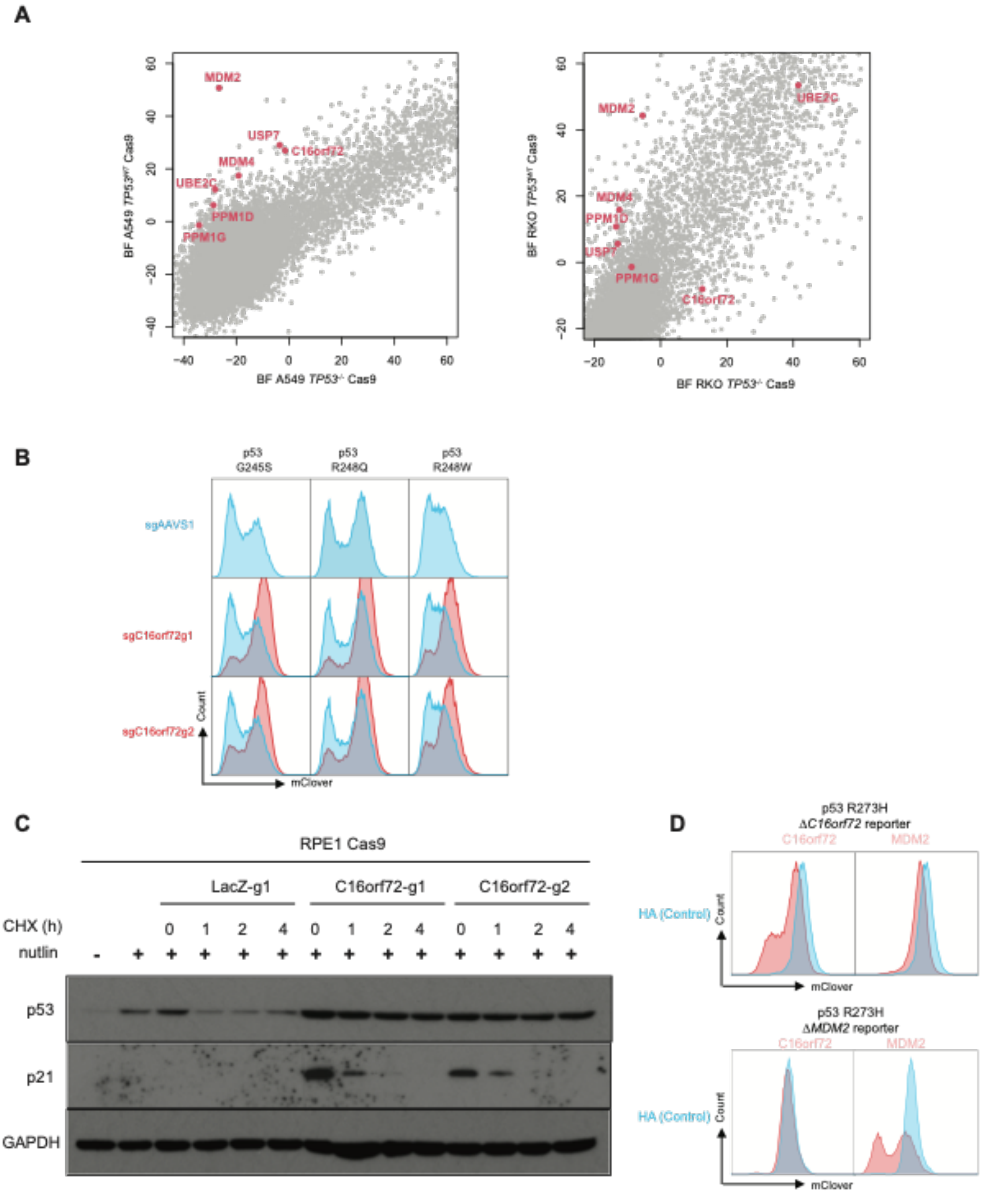

Appendix Figure S6. C16orf72/HAPSTR1 regulates wild-type and mutant p53 stability.
A. Synthetic viability screen in A549 and RKO cells. Bayesian Factors (BF) as a measurement of essentiality (high values indicate a lethal gene) are shown for all protein-coding genes in p53 wild-type (y-axis) versus p53 null (x-axis) background. All BFs were computed using the BAGEL2 algorithm (Kim & Hart, 2021). Hits identified from the RPE1 screens are highlighted in red.
B. Flow cytometry blots depicting the levels of p53 G245S, R248Q, and R248W in the p53-mClover RPE1 reporter cells upon depletion of *C16orf72/HAPSTR1*.
C. Cycloheximide (CHX) chase experiment showing p53 and p21 levels upon nutlin treatment with or without knock-out of C16orf72, and upon translation inhibition with CHX at various time points. Results are reproducible over biological triplicates.
D. Flow cytometry blots depicting the level of $p53^{R273H}$-mClover protein levels in reporters that have been clonally depleted of *C16orf72*, and upon the overexpression of HA (control), full-length *C16orf72*, and *MDM2*. Results are reproducible over biological triplicates.

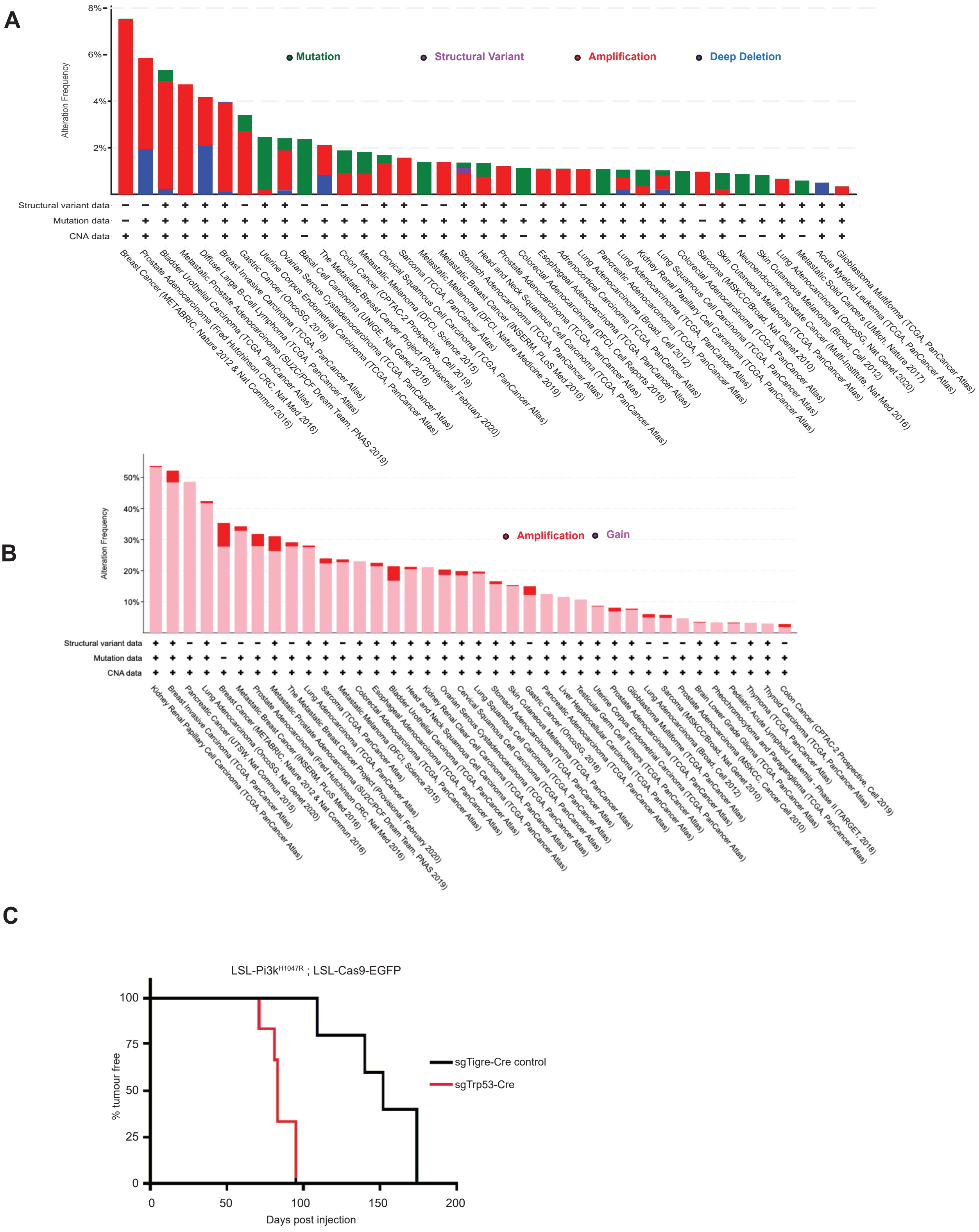

Appendix Figure S7. *C16orf72/HAPSTR1* functions as oncogene and regulates p53 stability in the mammary gland.
A. cBioPortal OncoPrint displaying mutations, amplifications, deletions and structural variants of *C16orf72/HAPSTR1* in all cancers.
B. cBioPortal OncoPrint displaying amplifications and gains of *C16orf72/HAPSTR1* in all cancers.
C. Kaplan-Meier plot comparing the tumor-free survival of tumor-prone LSL-*Pi3k*^{H1047R} mice intraductally injected with lentiviral particles expressing Cre as well as a sgRNA targeting *Trp53* (sg*Trp53*-Cre) or the control safe harbor locus Tigre (sg*Tigre*-Cre). (n=5 for each condition, log-rank test was used for statistical analysis.)
