# Supplementary figures and images for: Genome-wide CRISPR screens identify novel regulators of wild-type and mutant p53 stability
Source: Mol Syst Biol. 2024 Apr 5;20(6):719–40. doi: 10.1038/s44320-024-00032-x (PMC11148184; doi:10.1038/s44320-024-00032-x)

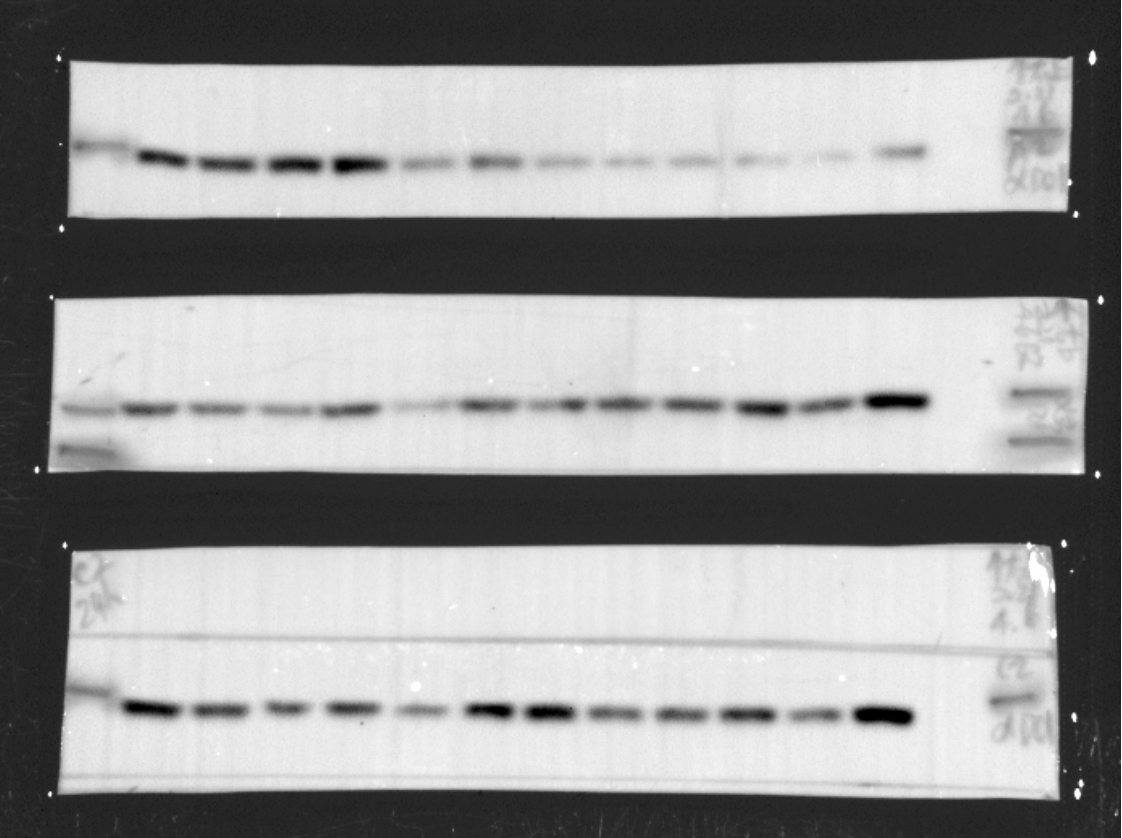

Supplement: Supplementary file 11 — Source data Fig. 2 [file 44320_2024_32_MOESM11_ESM.zip › Figure 2/Figure 2F/p53-merged.tif]

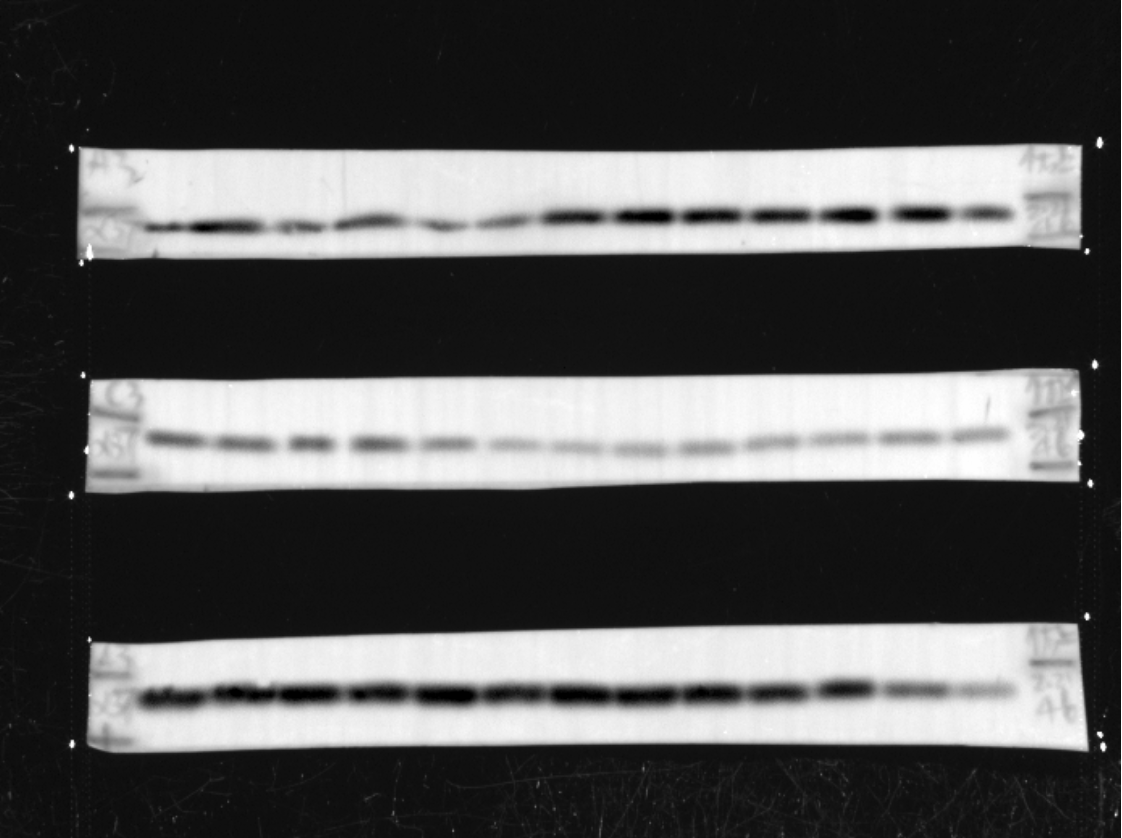

Supplement: Supplementary file 11 — Source data Fig. 2 [file 44320_2024_32_MOESM11_ESM.zip › Figure 2/Figure 2F/GAPDH-merged.tif]

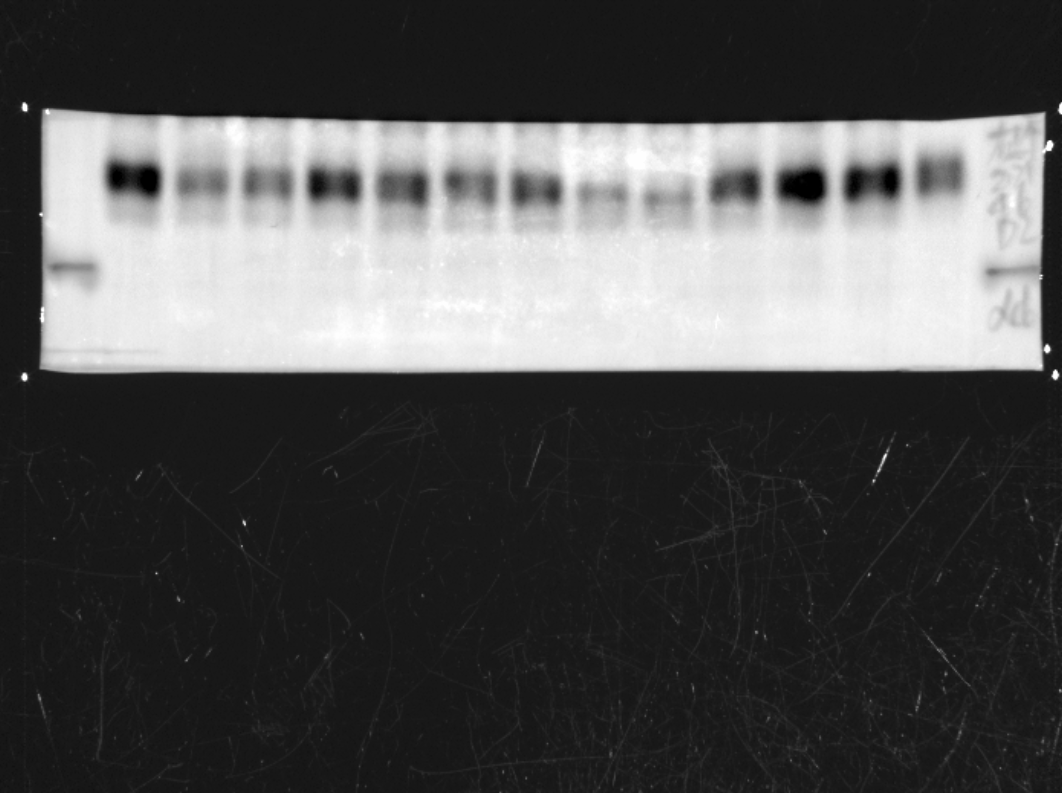

Supplement: Supplementary file 11 — Source data Fig. 2 [file 44320_2024_32_MOESM11_ESM.zip › Figure 2/Figure 2F/CCDC6-merged.tif]

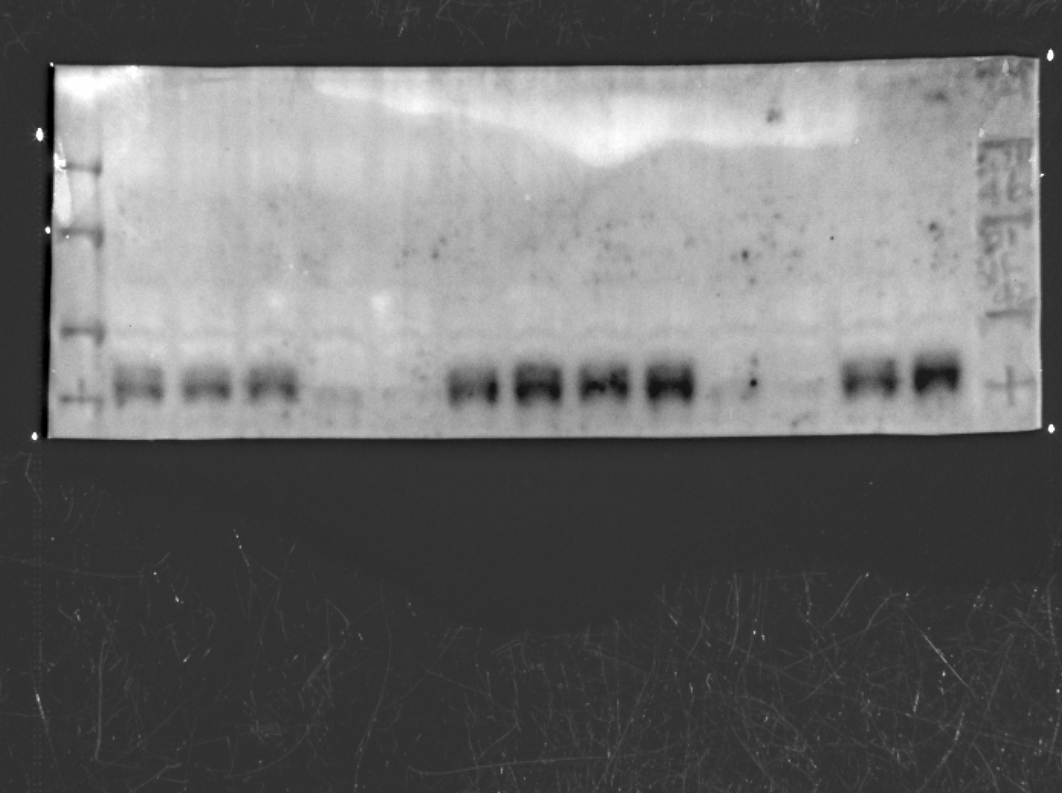

Supplement: Supplementary file 11 — Source data Fig. 2 [file 44320_2024_32_MOESM11_ESM.zip › Figure 2/Figure 2F/FBXO42-merged.tif]

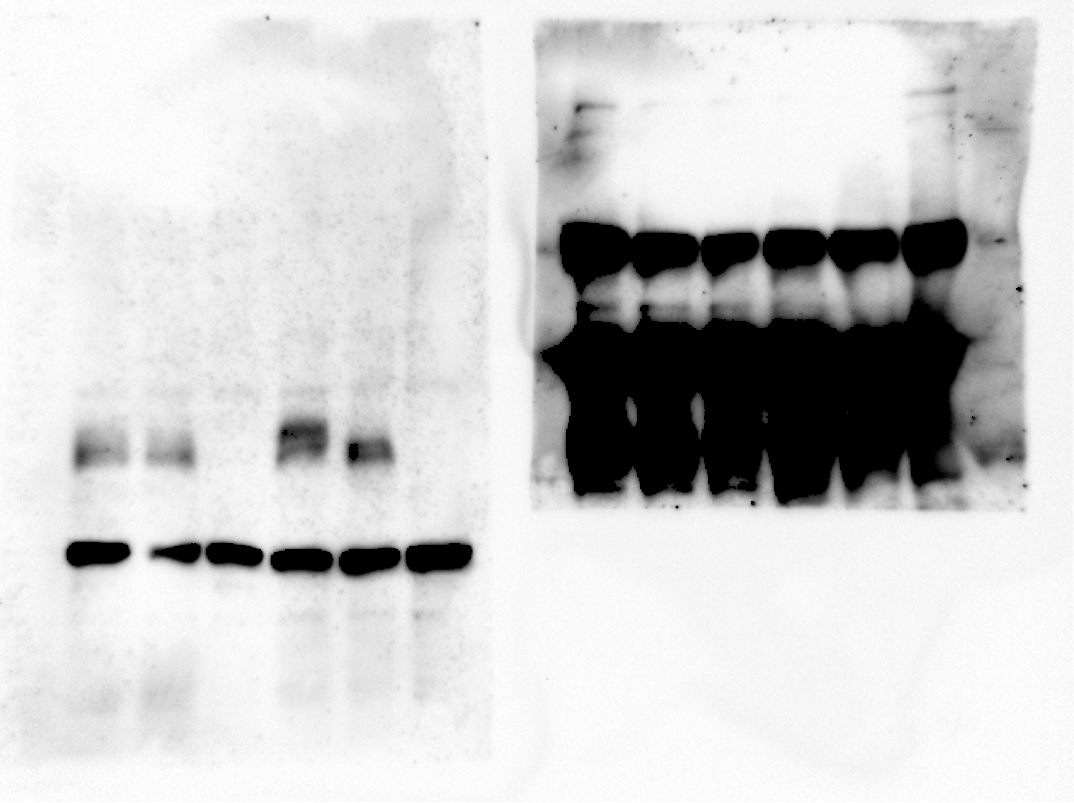

Supplement: Supplementary file 11 — Source data Fig. 2 [file 44320_2024_32_MOESM11_ESM.zip › Figure 2/Figure 2E/FBXO42-UpperLeft.tif]

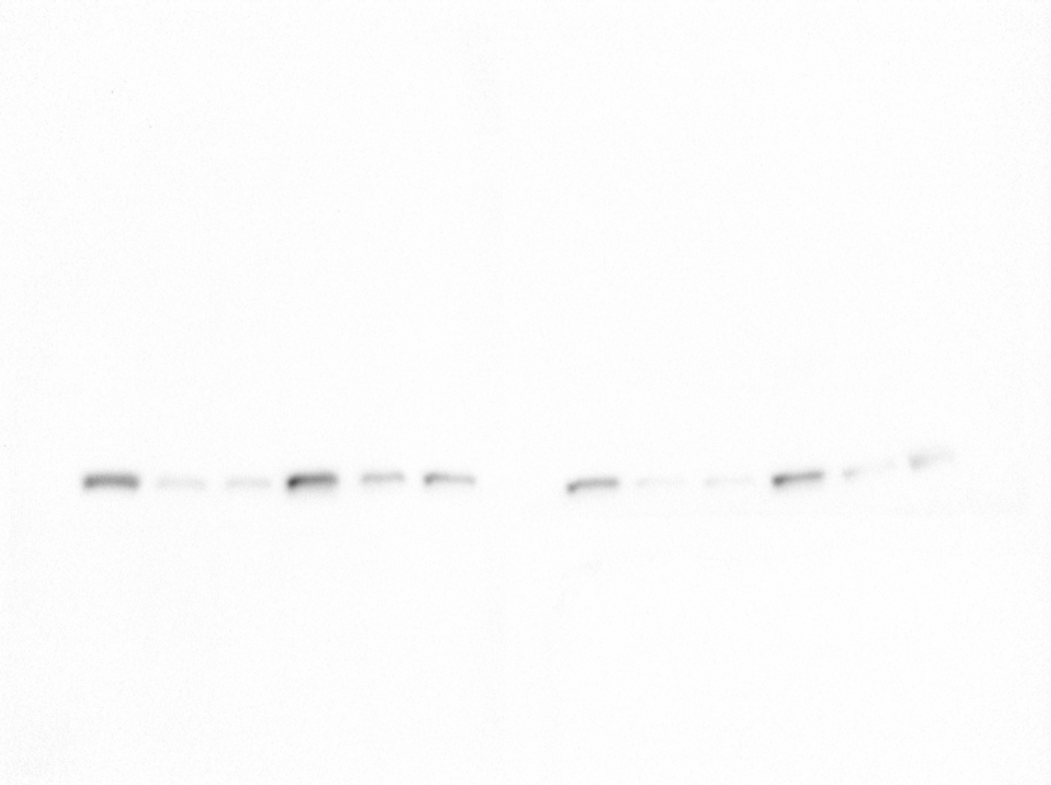

Supplement: Supplementary file 11 — Source data Fig. 2 [file 44320_2024_32_MOESM11_ESM.zip › Figure 2/Figure 2E/p53DO1HRP-Left.tif]

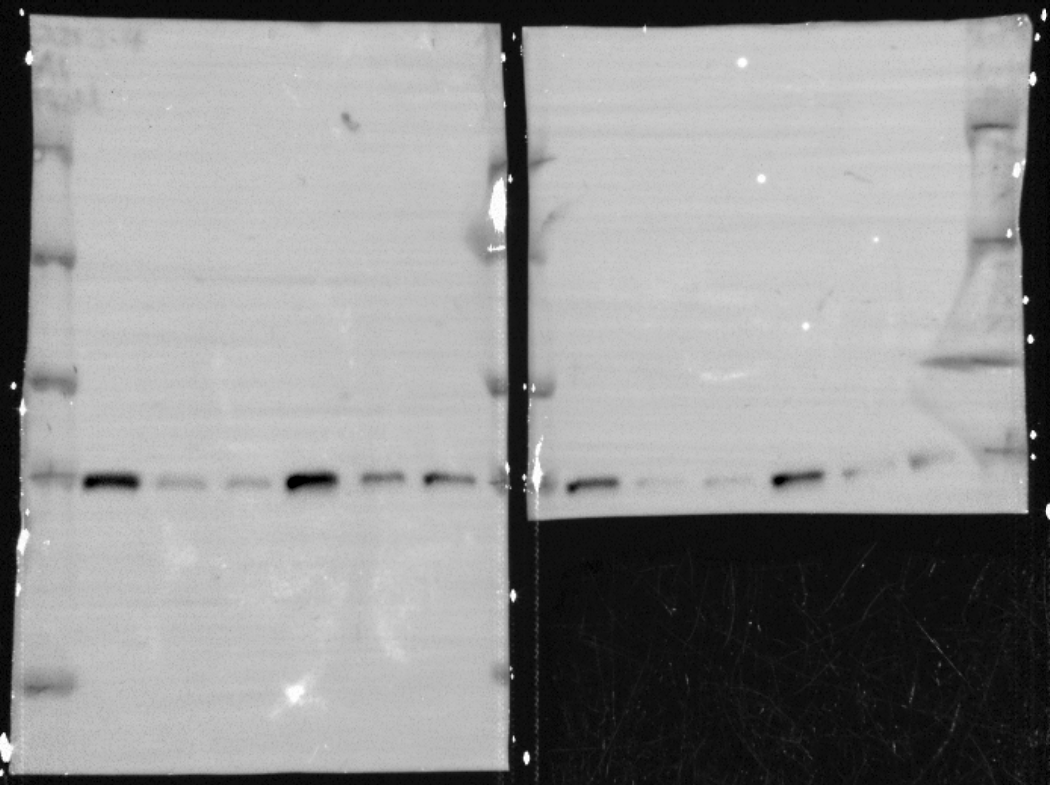

Supplement: Supplementary file 11 — Source data Fig. 2 [file 44320_2024_32_MOESM11_ESM.zip › Figure 2/Figure 2E/p53-merged.tif]

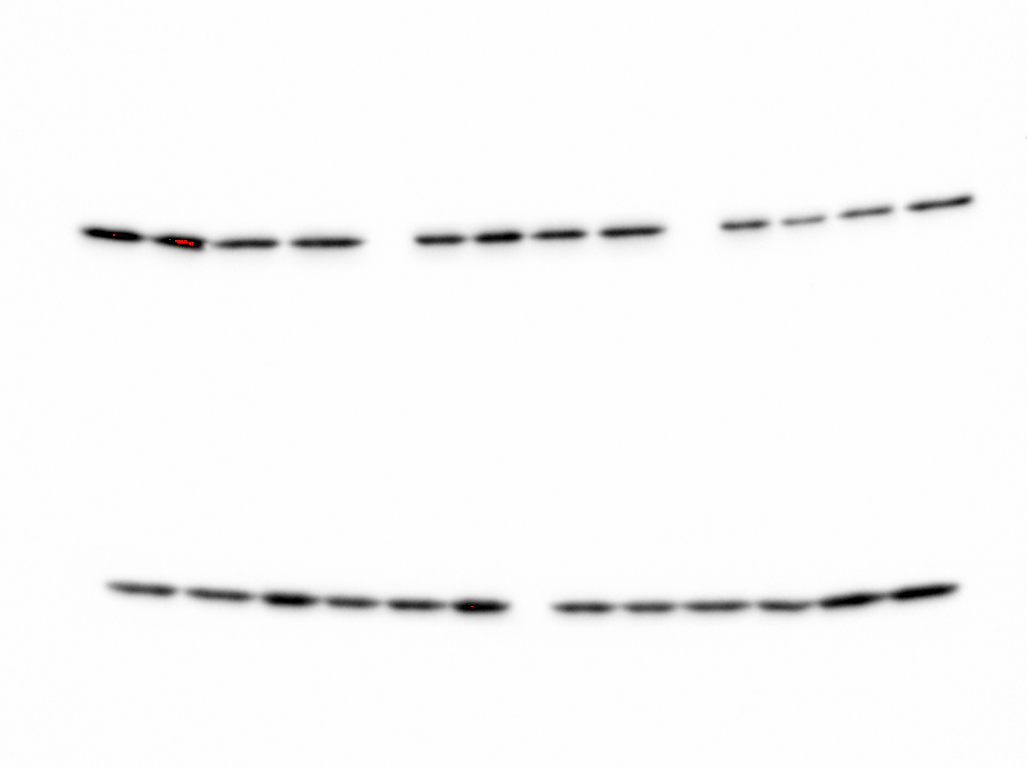

Supplement: Supplementary file 11 — Source data Fig. 2 [file 44320_2024_32_MOESM11_ESM.zip › Figure 2/Figure 2E/GAPDH-LowerLeft.tif]

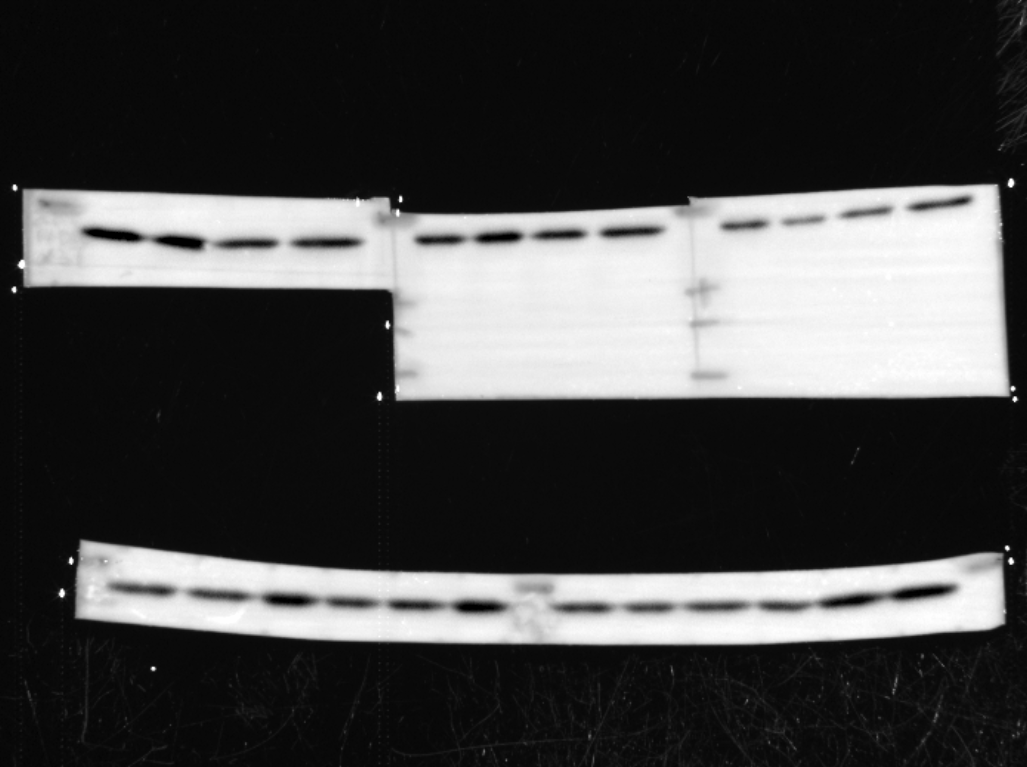

Supplement: Supplementary file 11 — Source data Fig. 2 [file 44320_2024_32_MOESM11_ESM.zip › Figure 2/Figure 2E/GAPDH-merged.tif]

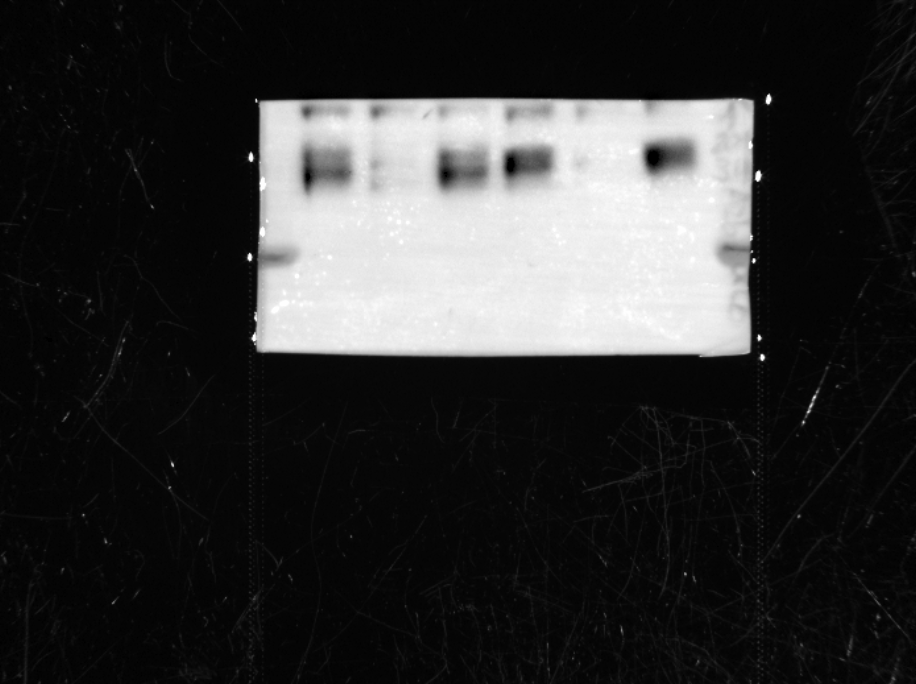

Supplement: Supplementary file 11 — Source data Fig. 2 [file 44320_2024_32_MOESM11_ESM.zip › Figure 2/Figure 2E/CCDC6-merged.tif]

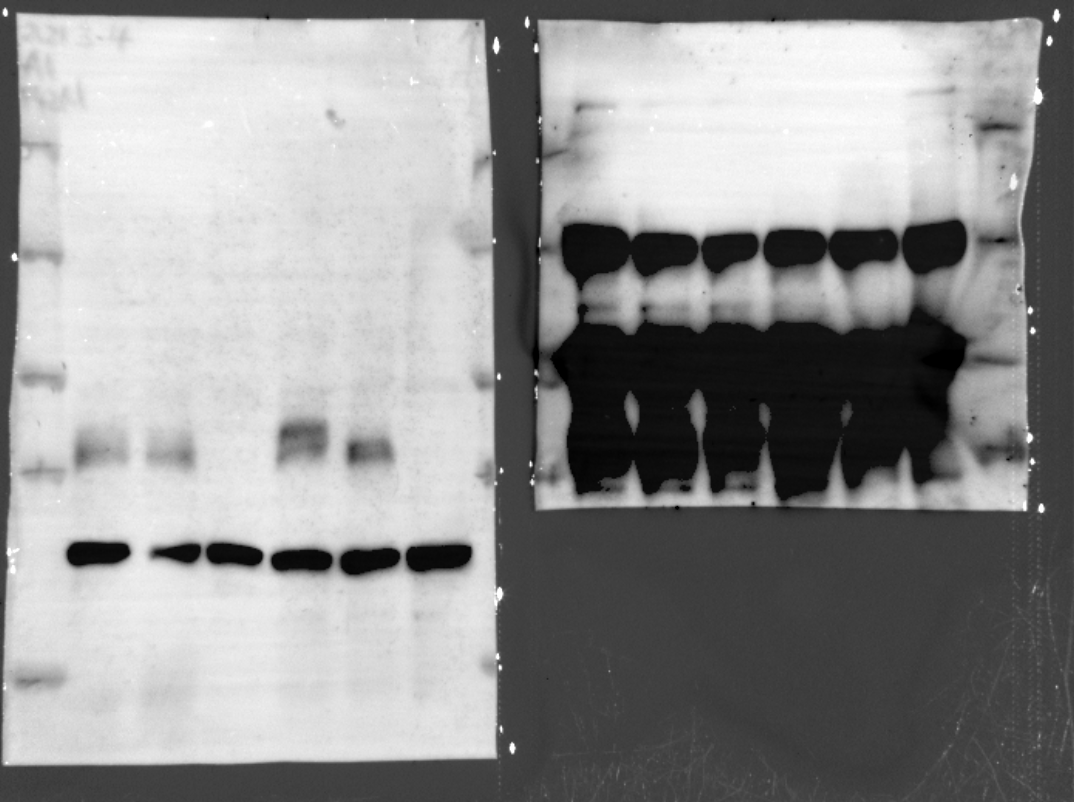

Supplement: Supplementary file 11 — Source data Fig. 2 [file 44320_2024_32_MOESM11_ESM.zip › Figure 2/Figure 2E/FBXO42-merged.tif]

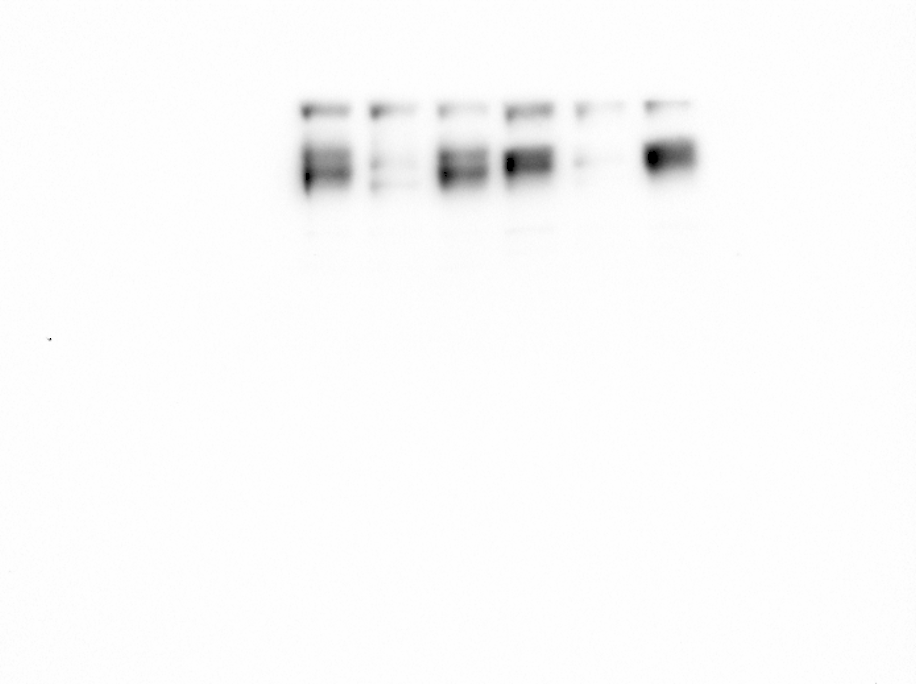

Supplement: Supplementary file 11 — Source data Fig. 2 [file 44320_2024_32_MOESM11_ESM.zip › Figure 2/Figure 2E/CCDC6-BottomDoublet.tif]

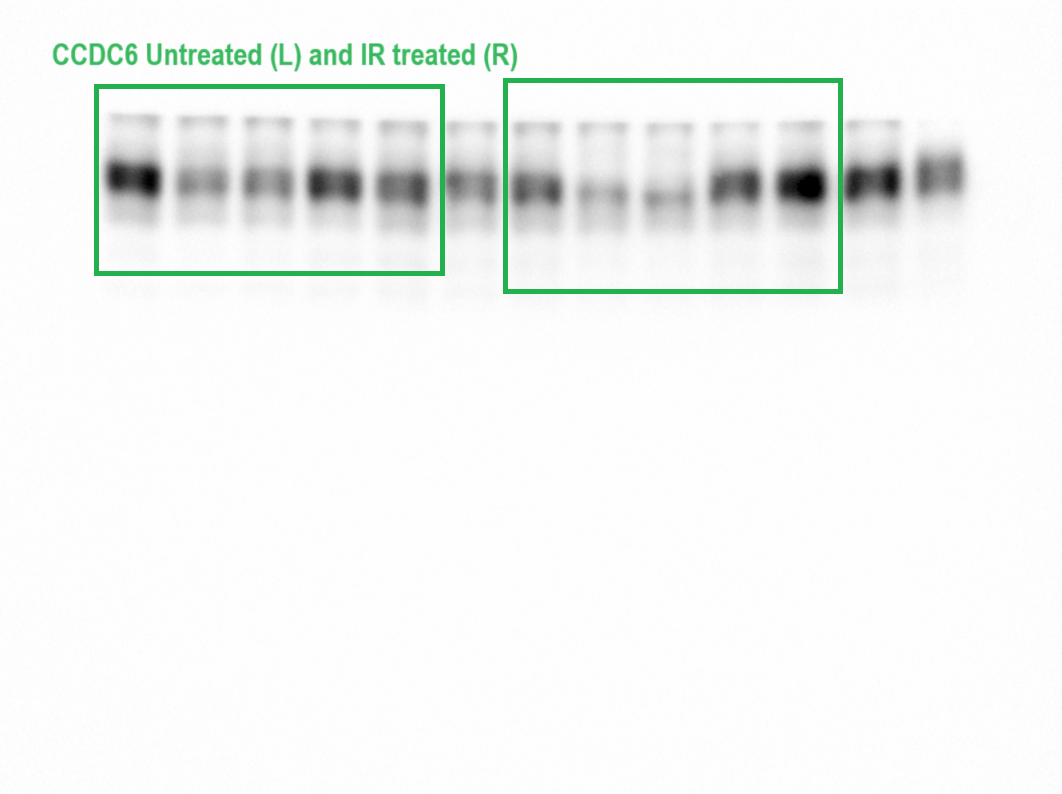

Supplement: Supplementary file 11 — Source data Fig. 2 [file 44320_2024_32_MOESM11_ESM.zip › Figure 2/Figure 2F/Annotated/CCDC6.tif]

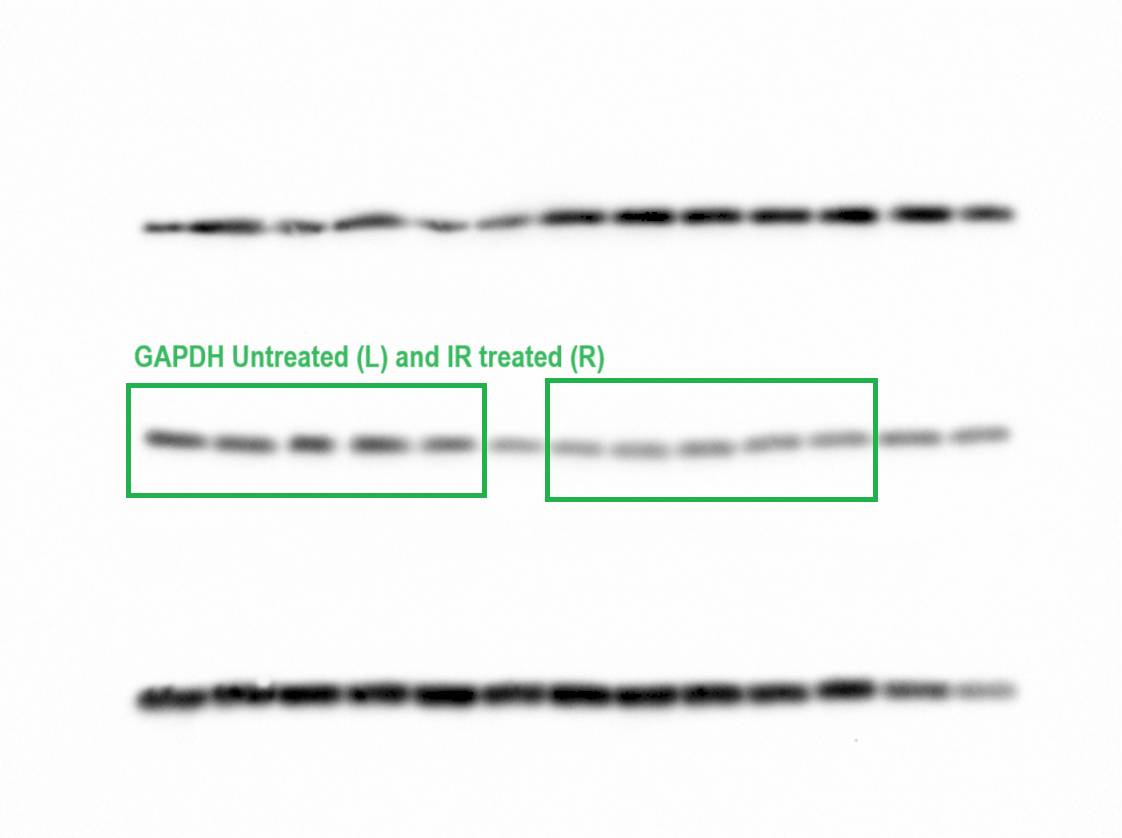

Supplement: Supplementary file 11 — Source data Fig. 2 [file 44320_2024_32_MOESM11_ESM.zip › Figure 2/Figure 2F/Annotated/GAPDH.tif]

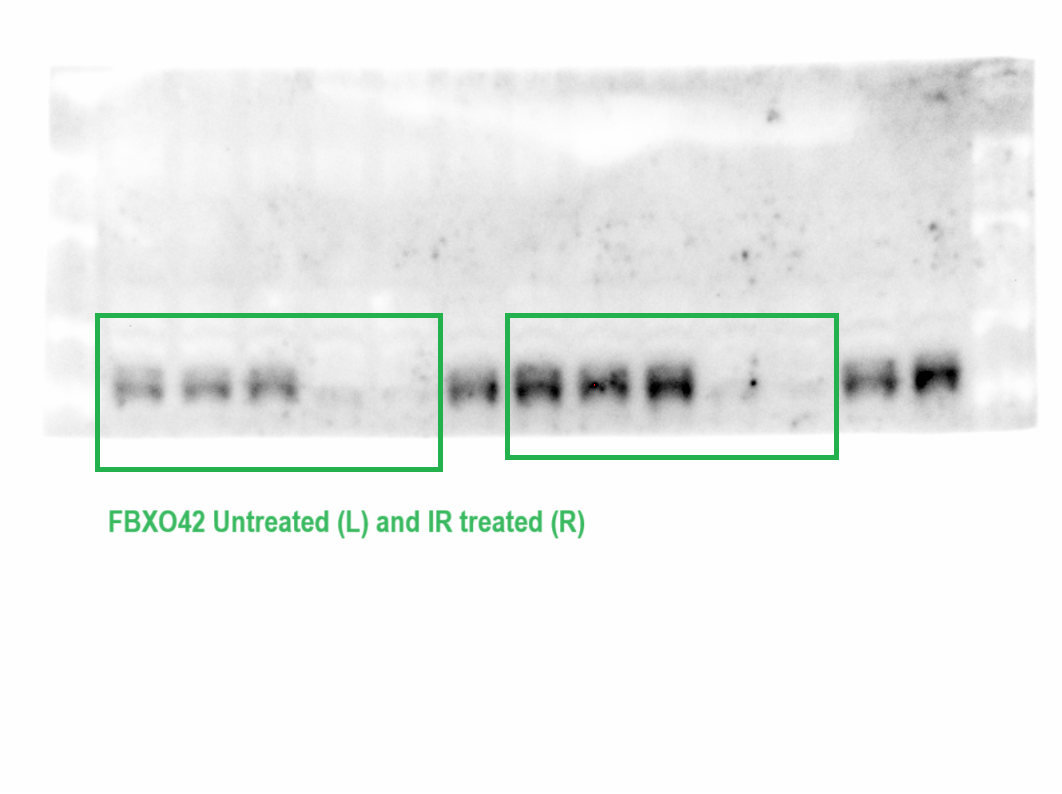

Supplement: Supplementary file 11 — Source data Fig. 2 [file 44320_2024_32_MOESM11_ESM.zip › Figure 2/Figure 2F/Annotated/FBXO42.tif]

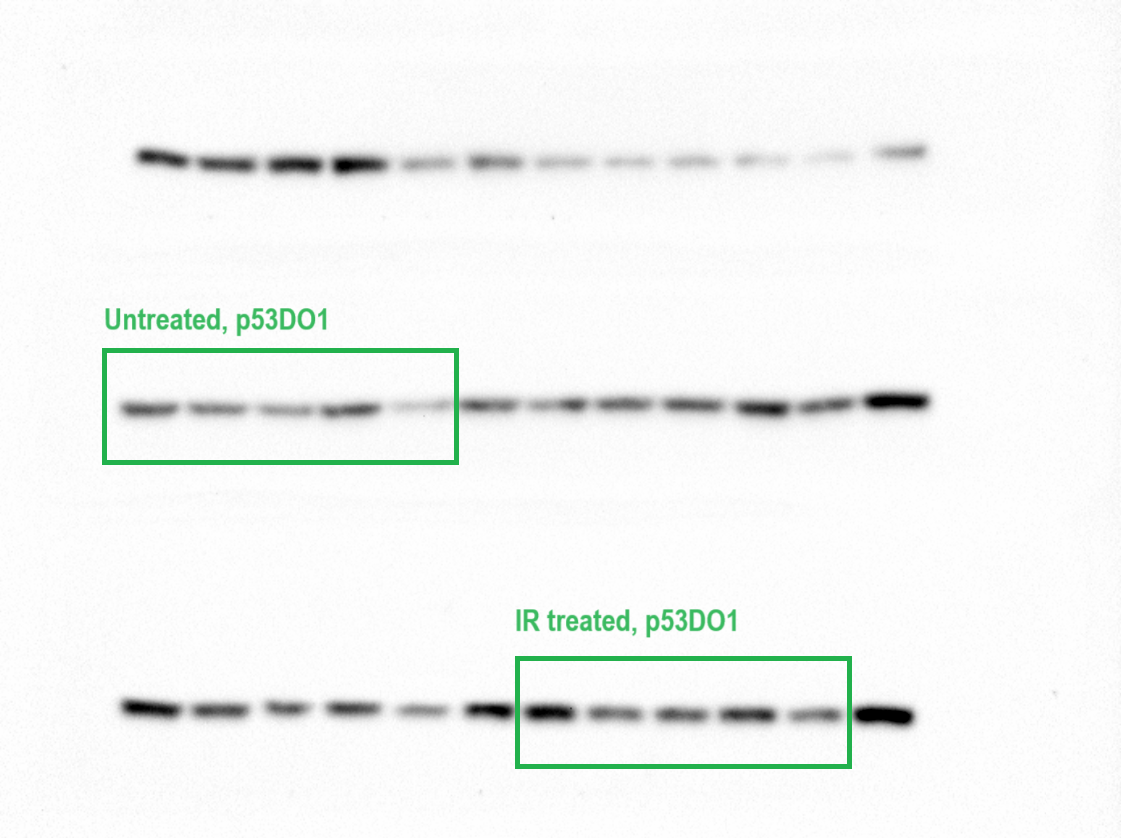

Supplement: Supplementary file 11 — Source data Fig. 2 [file 44320_2024_32_MOESM11_ESM.zip › Figure 2/Figure 2F/Annotated/p53.tif]

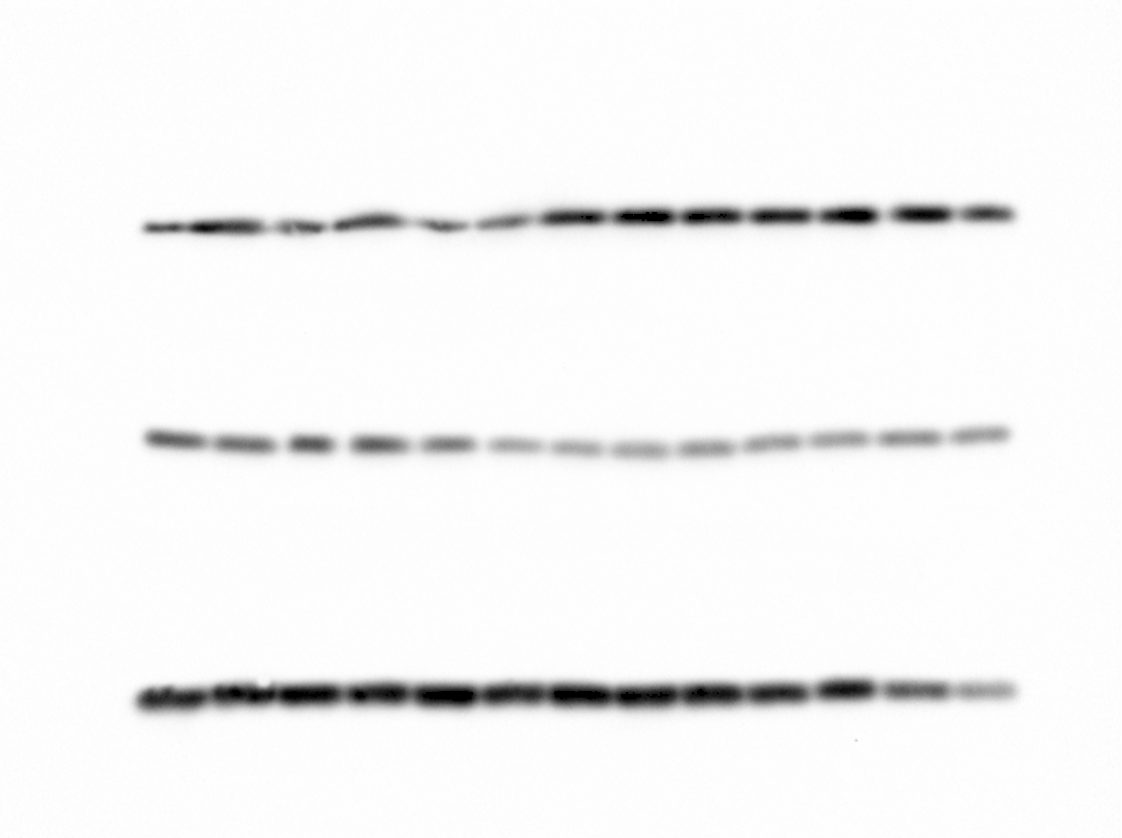

Supplement: Supplementary file 11 — Source data Fig. 2 [file 44320_2024_32_MOESM11_ESM.zip › Figure 2/Figure 2F/Non-annotated/YiQing L∩┐╜ 2021-04-08 01hr 37min_Exposure_37.5sec-GAPDH.tif]

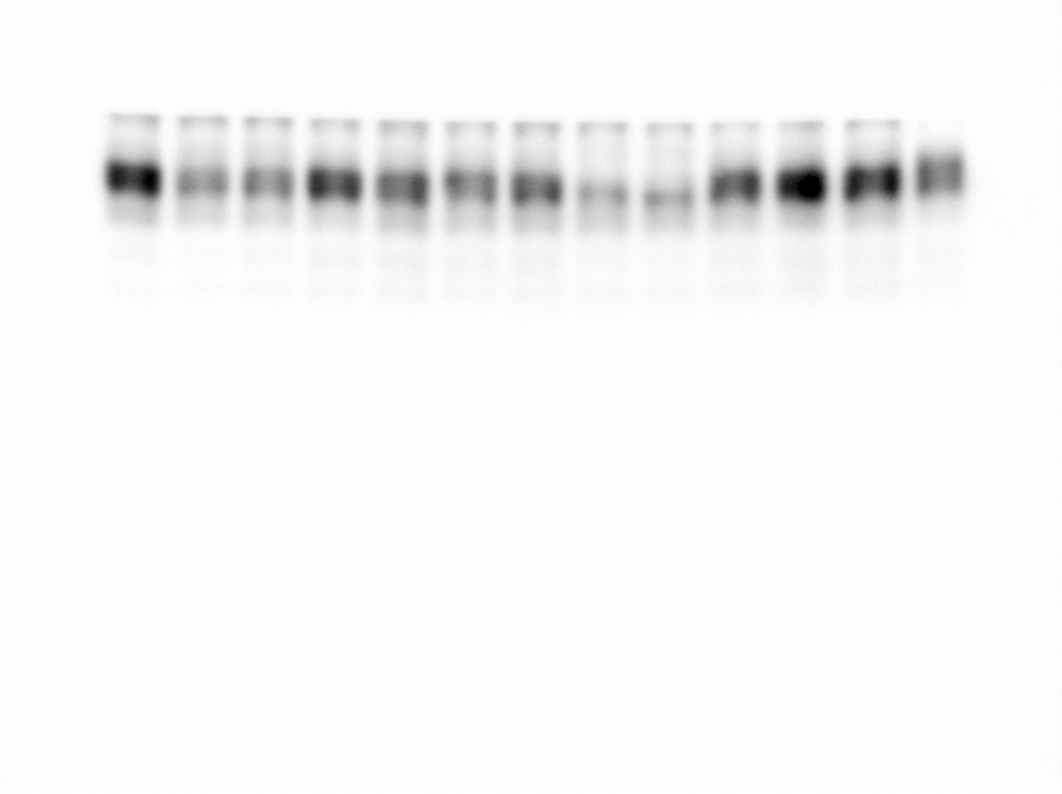

Supplement: Supplementary file 11 — Source data Fig. 2 [file 44320_2024_32_MOESM11_ESM.zip › Figure 2/Figure 2F/Non-annotated/YiQing L∩┐╜ 2021-04-08 18hr 22min_Exposure_5.9sec-CCDC6.tif]

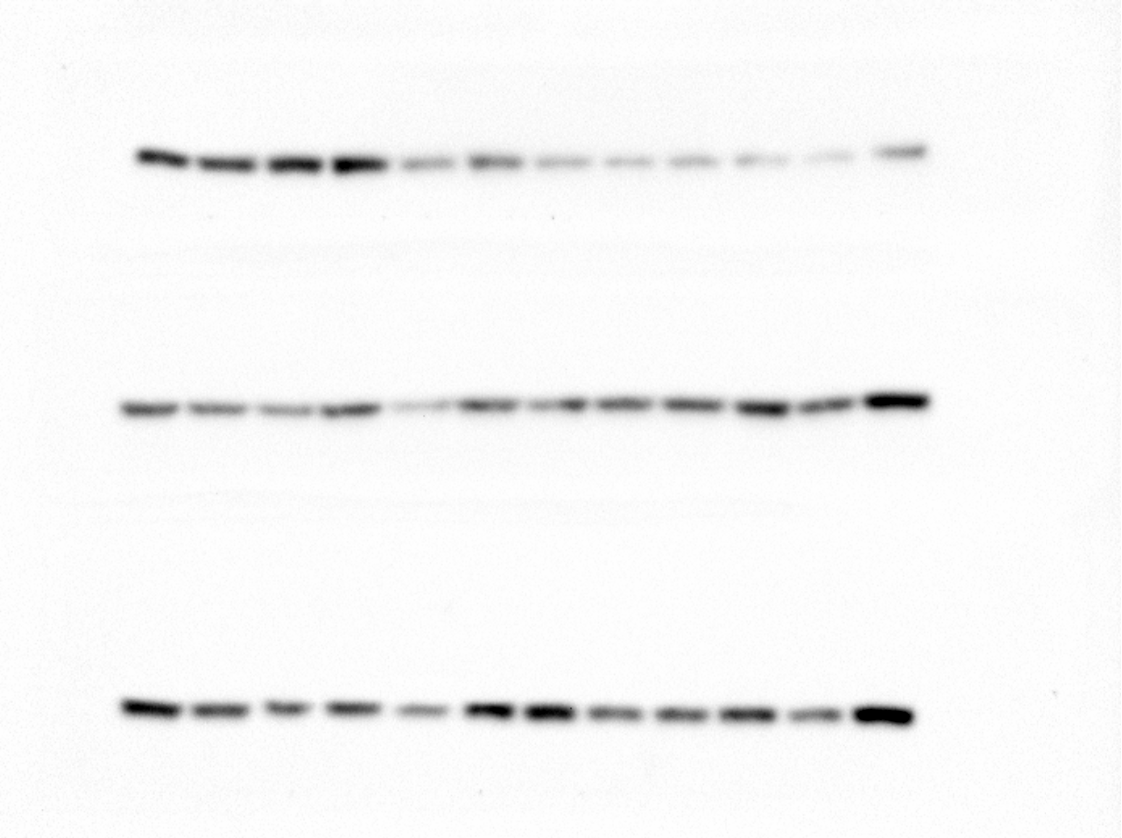

Supplement: Supplementary file 11 — Source data Fig. 2 [file 44320_2024_32_MOESM11_ESM.zip › Figure 2/Figure 2F/Non-annotated/YiQing L∩┐╜ 2021-04-08 01hr 21min_Exposure_161.4sec-p53DO1.tif]

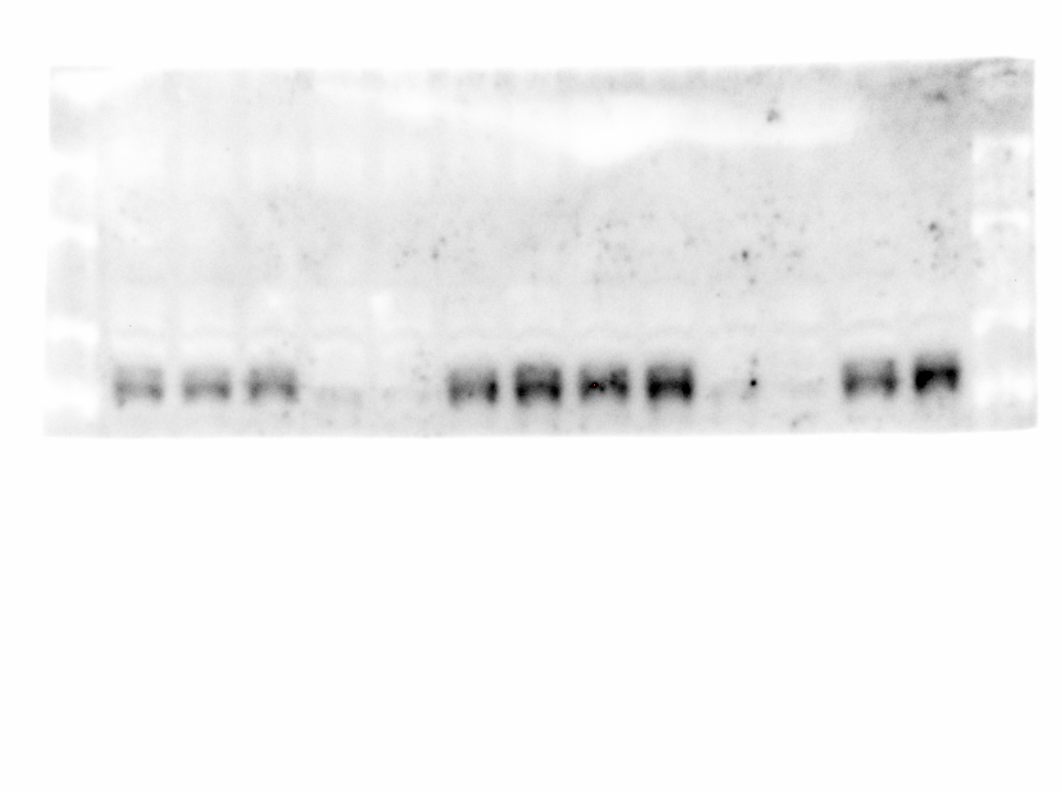

Supplement: Supplementary file 11 — Source data Fig. 2 [file 44320_2024_32_MOESM11_ESM.zip › Figure 2/Figure 2F/Non-annotated/YiQing L∩┐╜ 2021-04-08 18hr 57min_Exposure_15.6sec-FBXO42.tif]

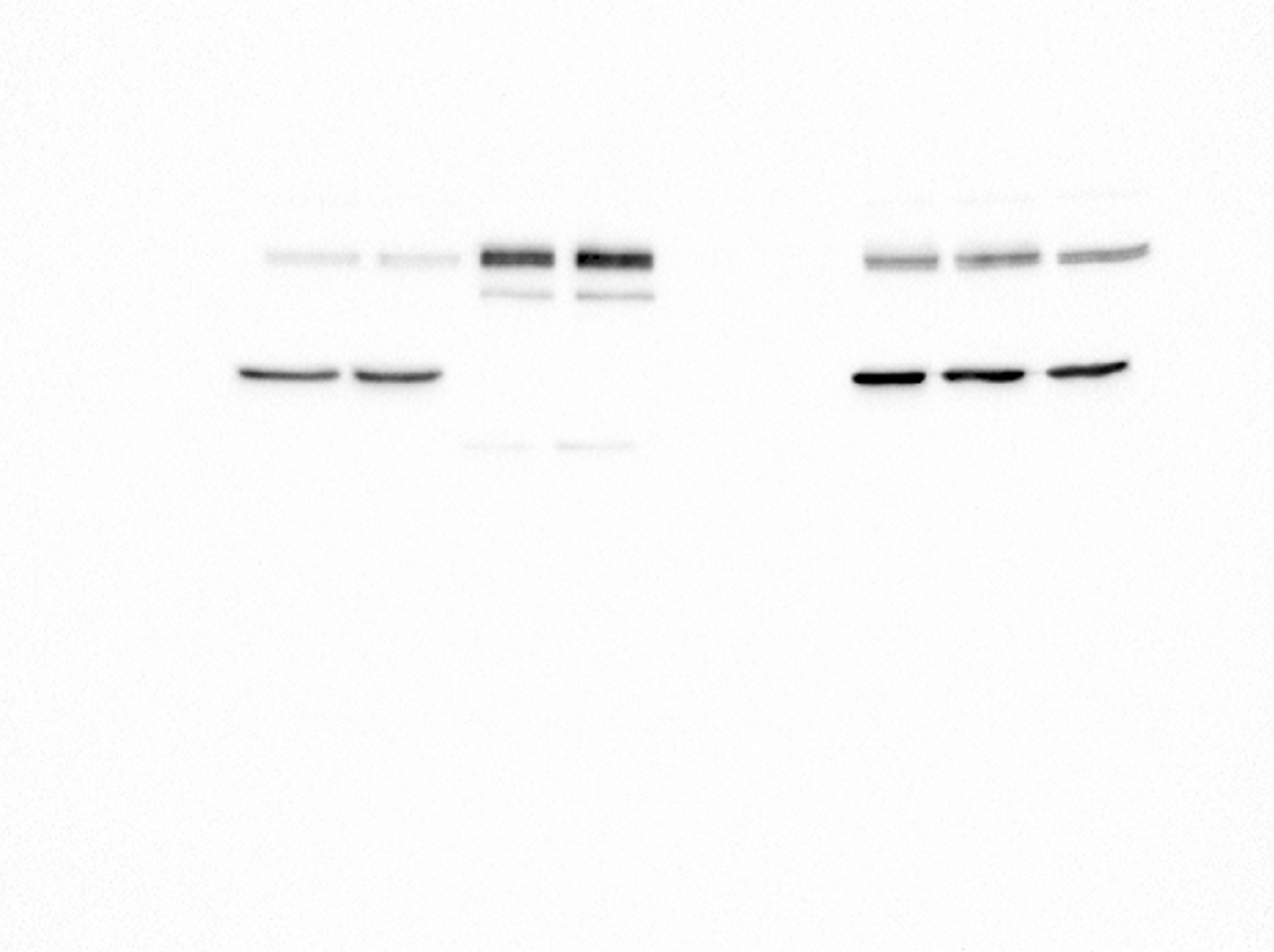

Supplement: Supplementary file 12 — Source data Fig. 3 [file 44320_2024_32_MOESM12_ESM.zip › Figure 3/Figure 3B/CCDC6-top-actin-bottom.tif]

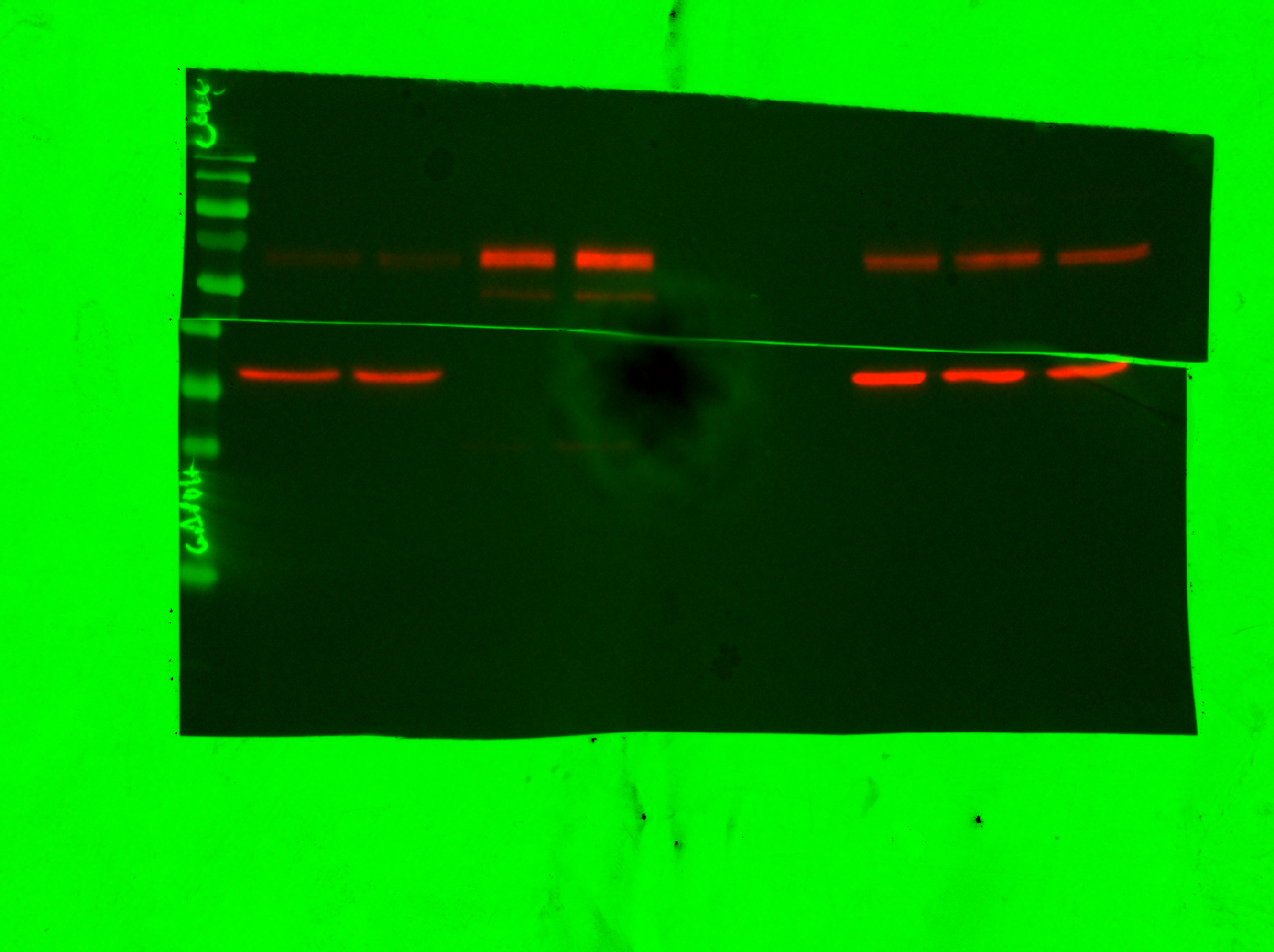

Supplement: Supplementary file 12 — Source data Fig. 3 [file 44320_2024_32_MOESM12_ESM.zip › Figure 3/Figure 3B/CCDC6-top-actin-bottom-merge.tif]

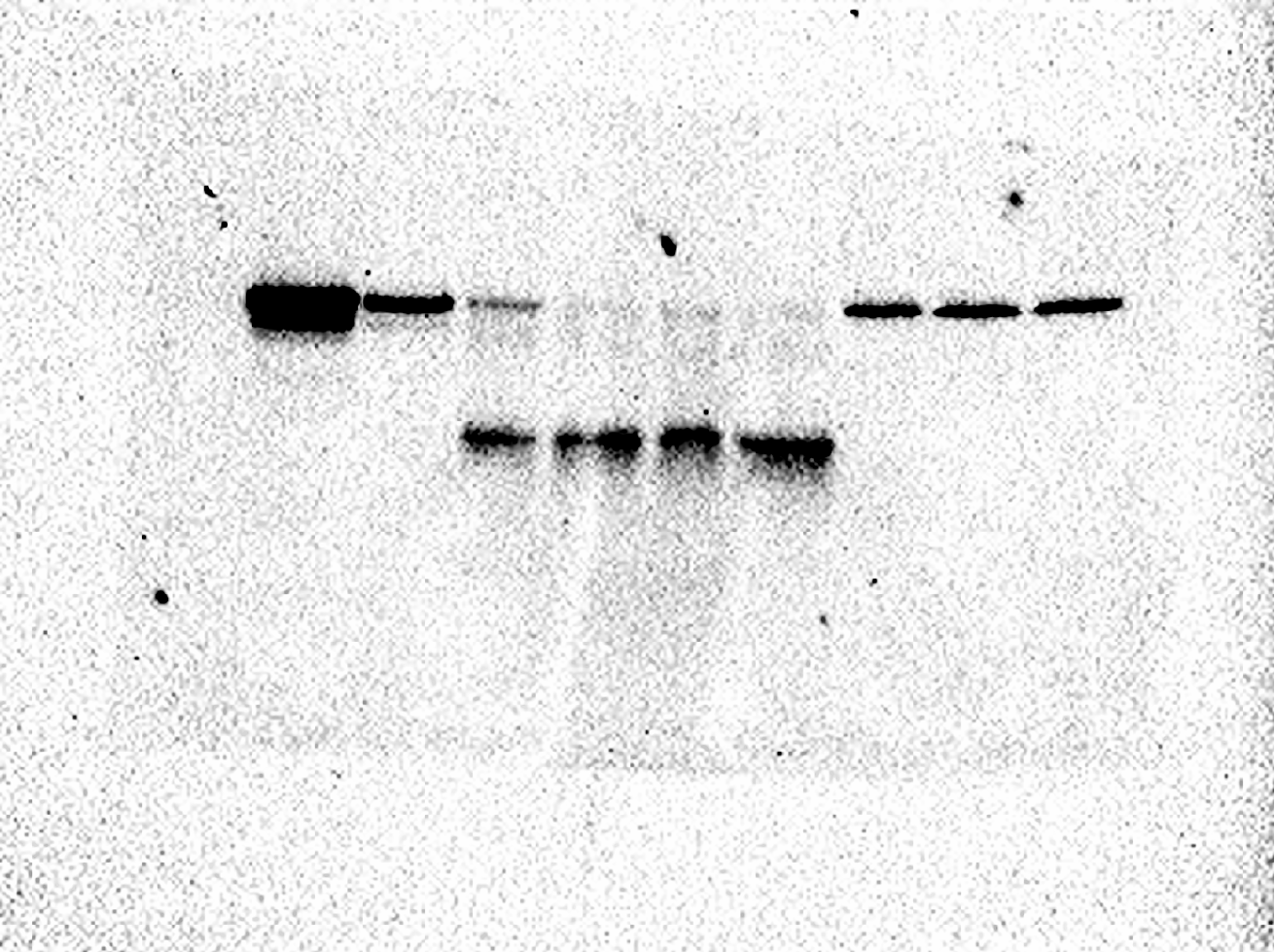

Supplement: Supplementary file 12 — Source data Fig. 3 [file 44320_2024_32_MOESM12_ESM.zip › Figure 3/Figure 3B/p53.tif]

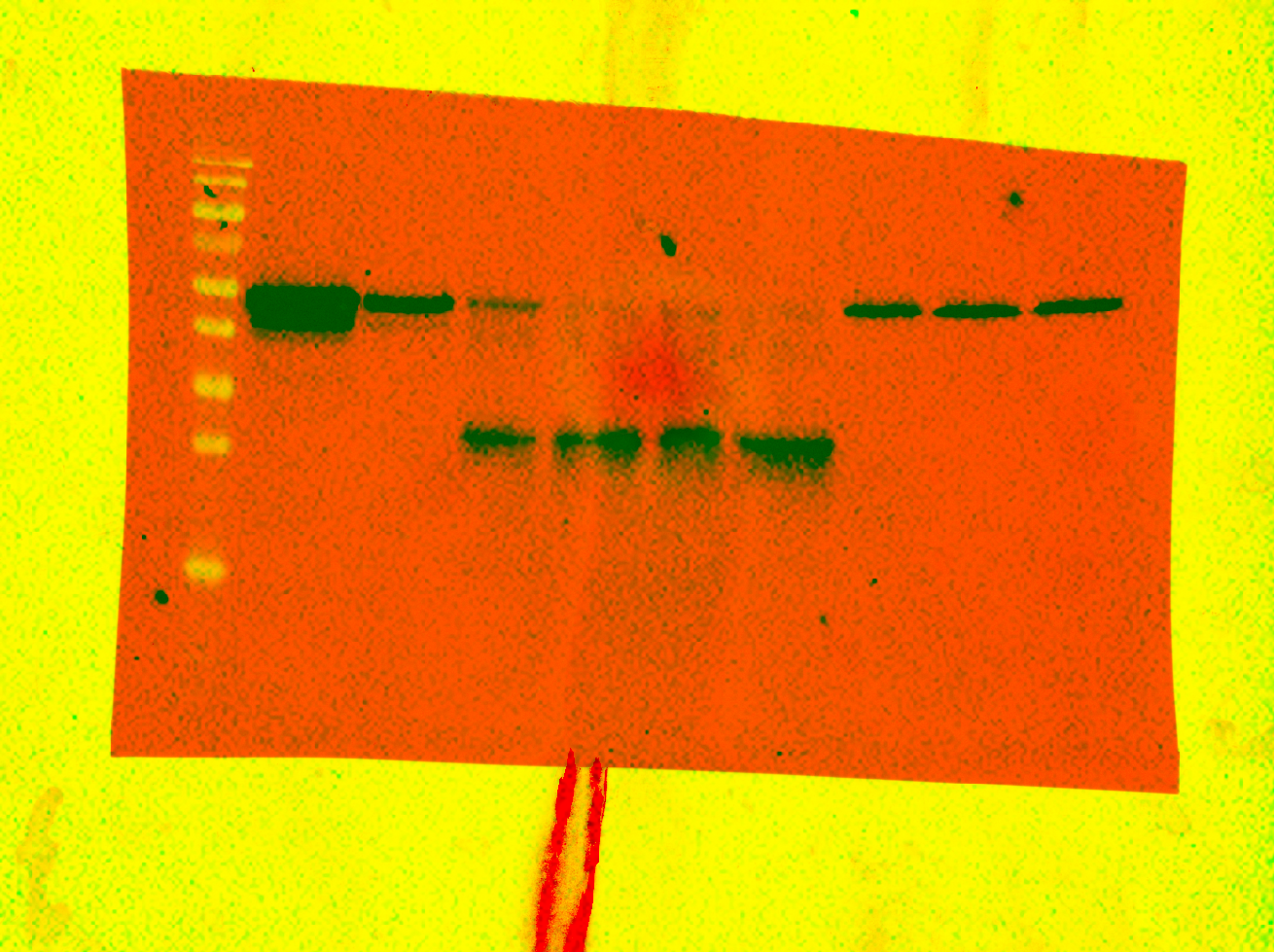

Supplement: Supplementary file 12 — Source data Fig. 3 [file 44320_2024_32_MOESM12_ESM.zip › Figure 3/Figure 3B/p53-merge.tif]

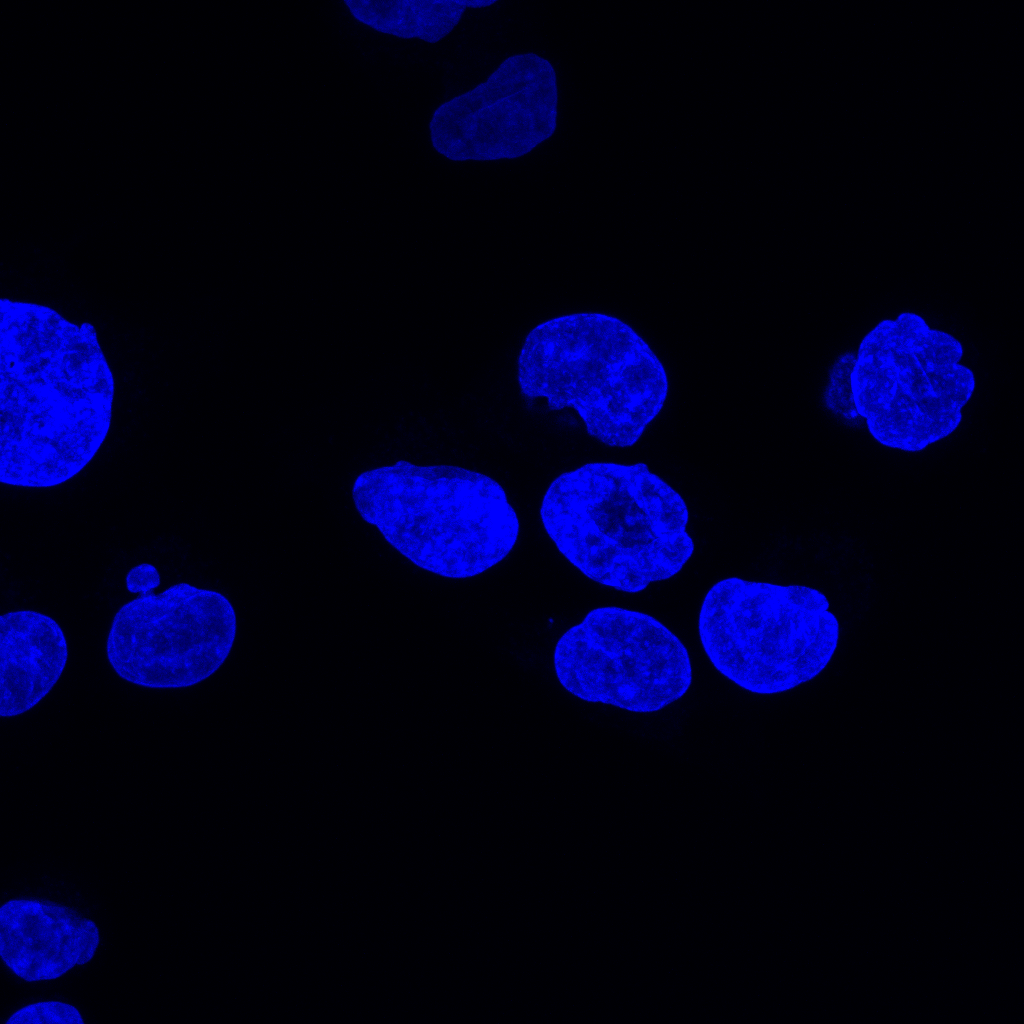

Supplement: Supplementary file 12 — Source data Fig. 3 [file 44320_2024_32_MOESM12_ESM.zip › Figure 3/Figure 3C/shc_1_60x_dapi.tif]

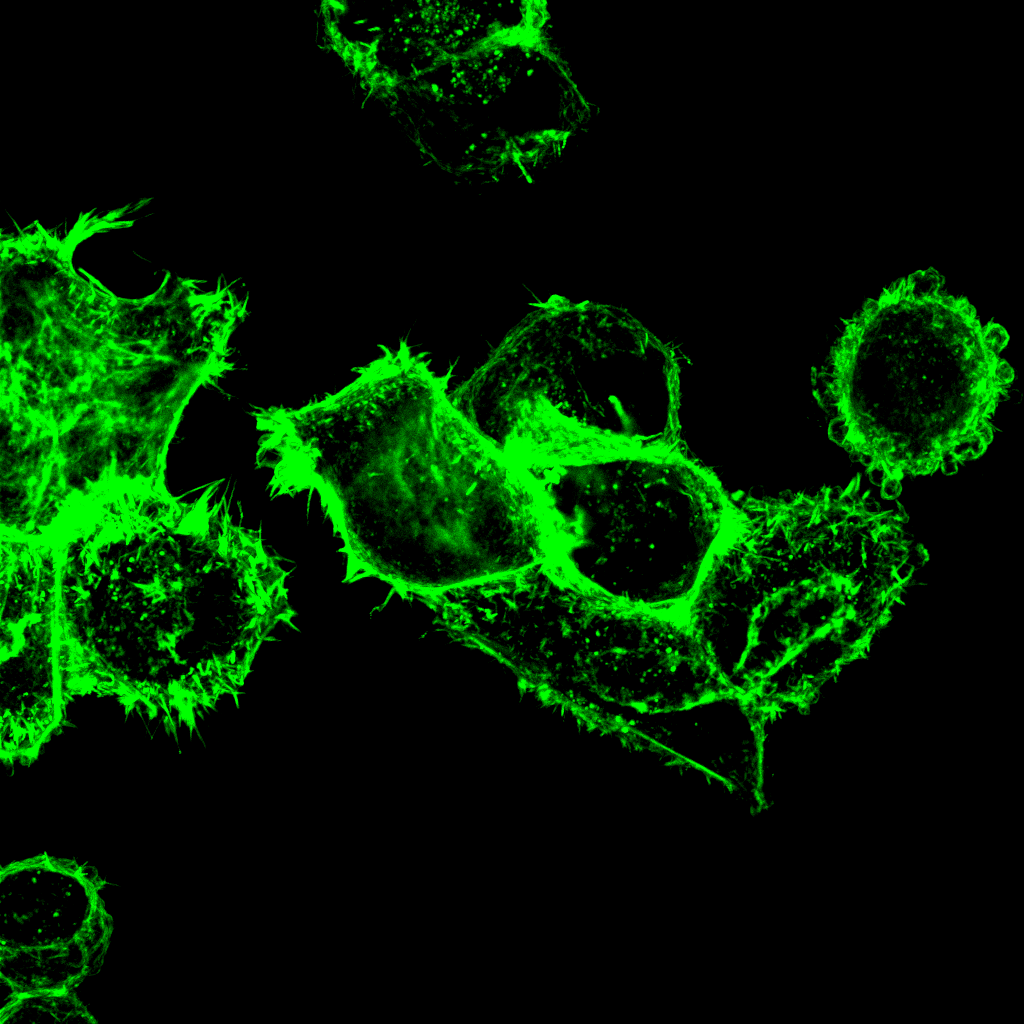

Supplement: Supplementary file 12 — Source data Fig. 3 [file 44320_2024_32_MOESM12_ESM.zip › Figure 3/Figure 3C/shc_1_60x_factin.tif]

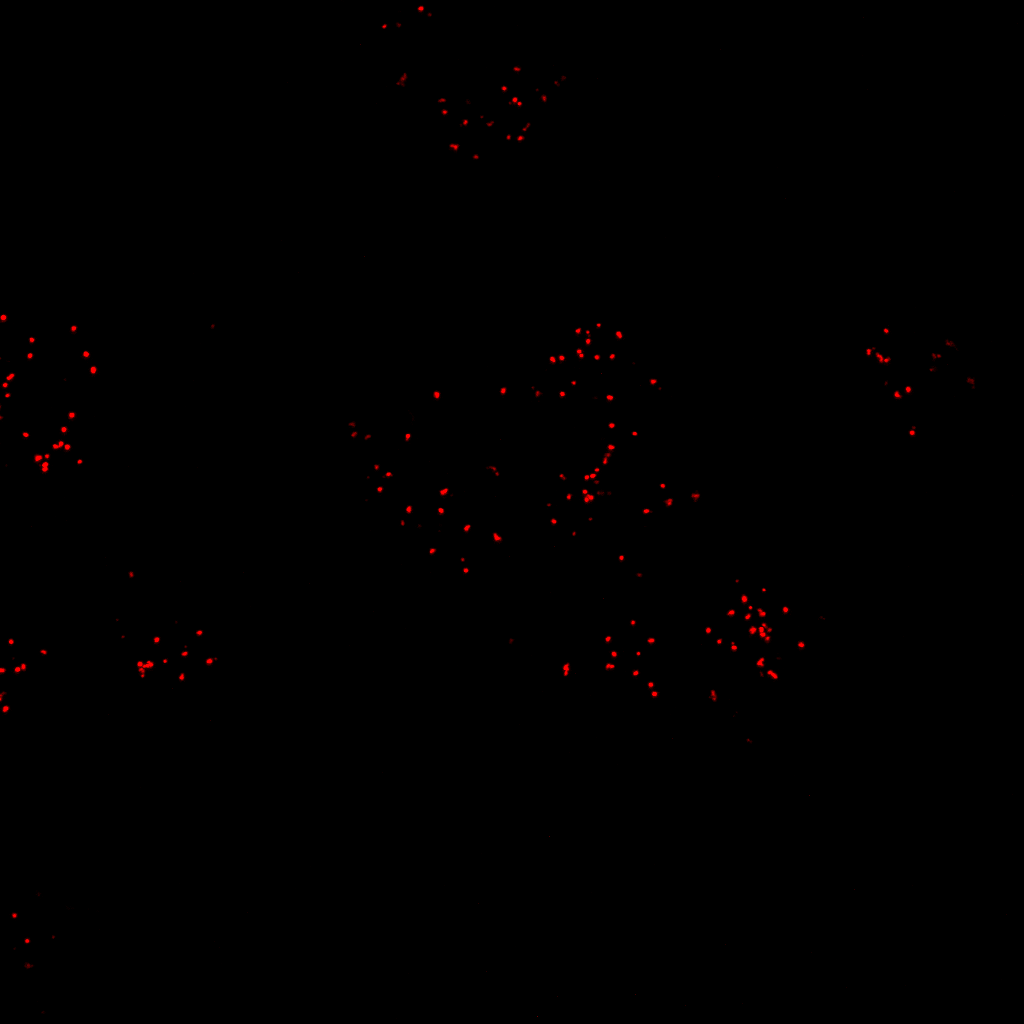

Supplement: Supplementary file 12 — Source data Fig. 3 [file 44320_2024_32_MOESM12_ESM.zip › Figure 3/Figure 3C/shc_1_60x_red.tif]

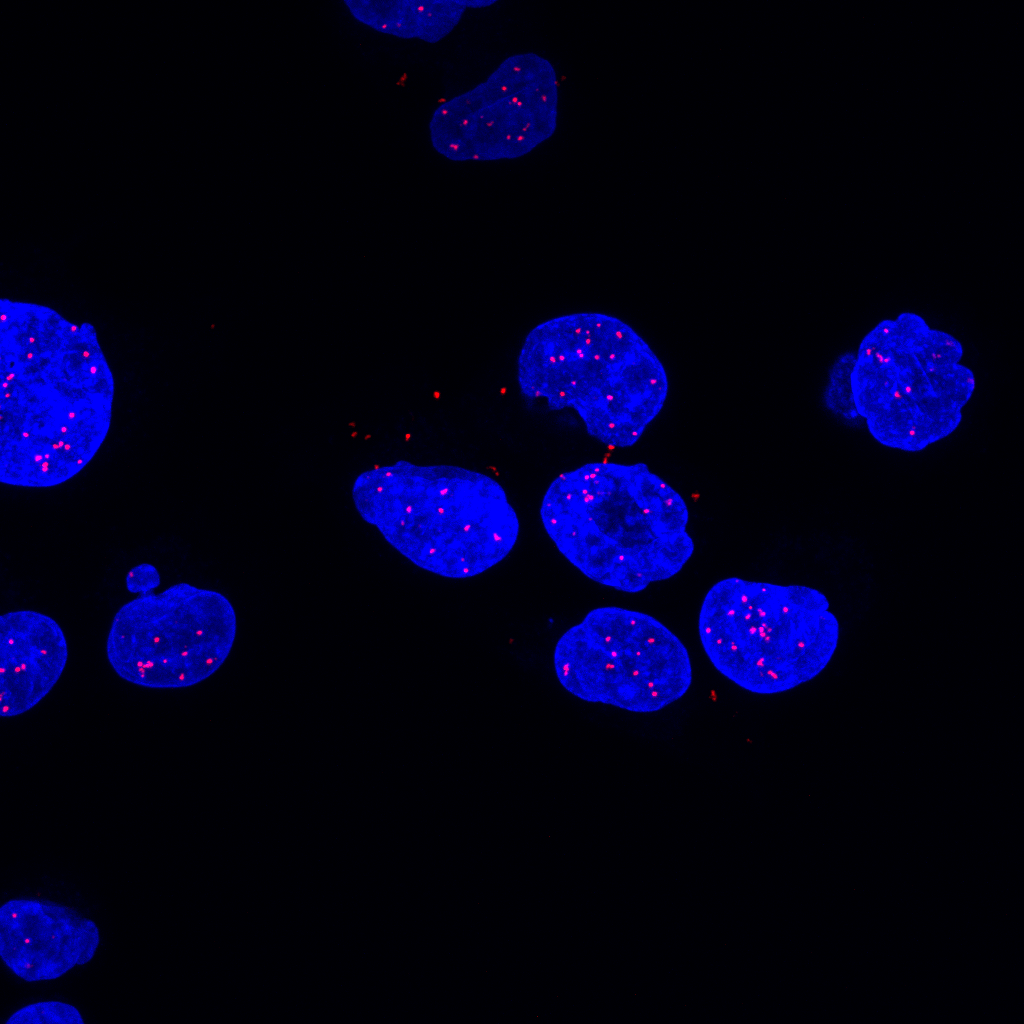

Supplement: Supplementary file 12 — Source data Fig. 3 [file 44320_2024_32_MOESM12_ESM.zip › Figure 3/Figure 3C/shc_1_60x_red_dapi.tif]

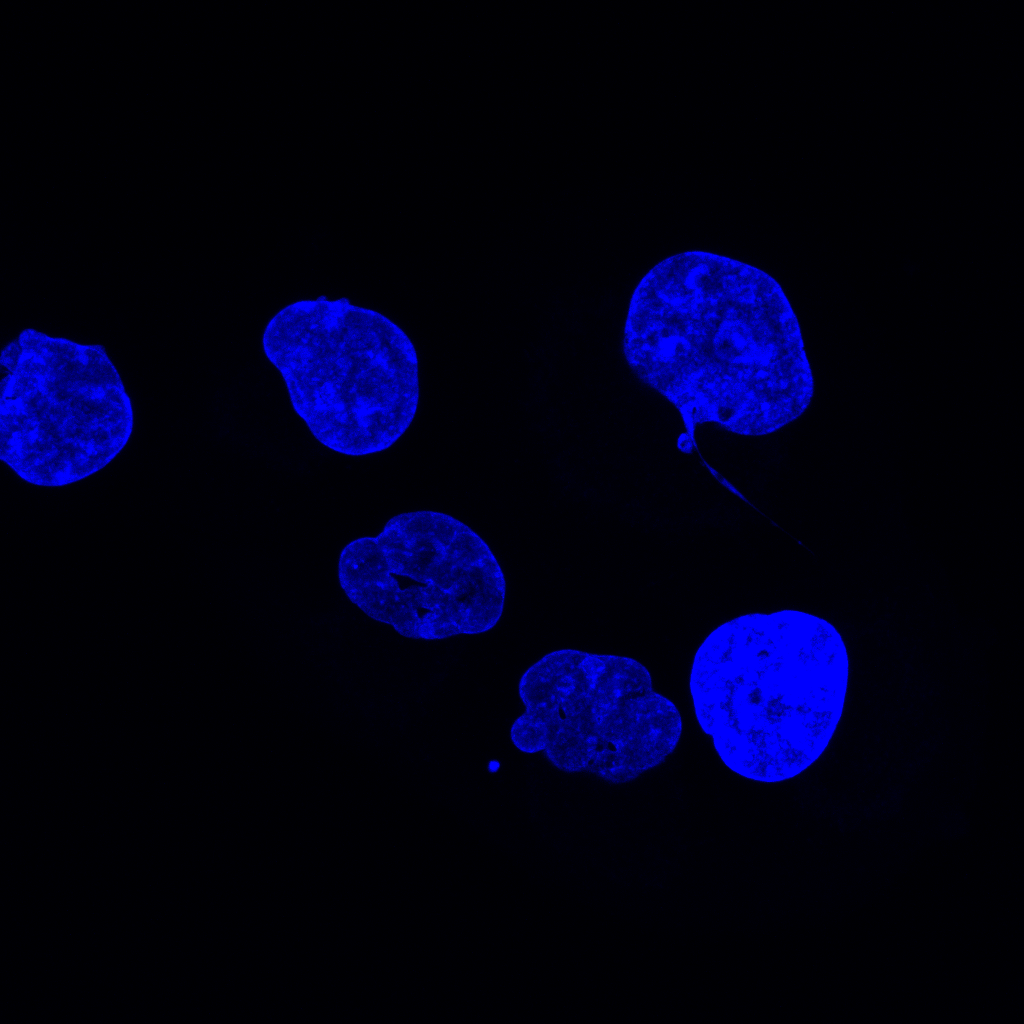

Supplement: Supplementary file 12 — Source data Fig. 3 [file 44320_2024_32_MOESM12_ESM.zip › Figure 3/Figure 3C/shp53_1_60x_dapi.tif]

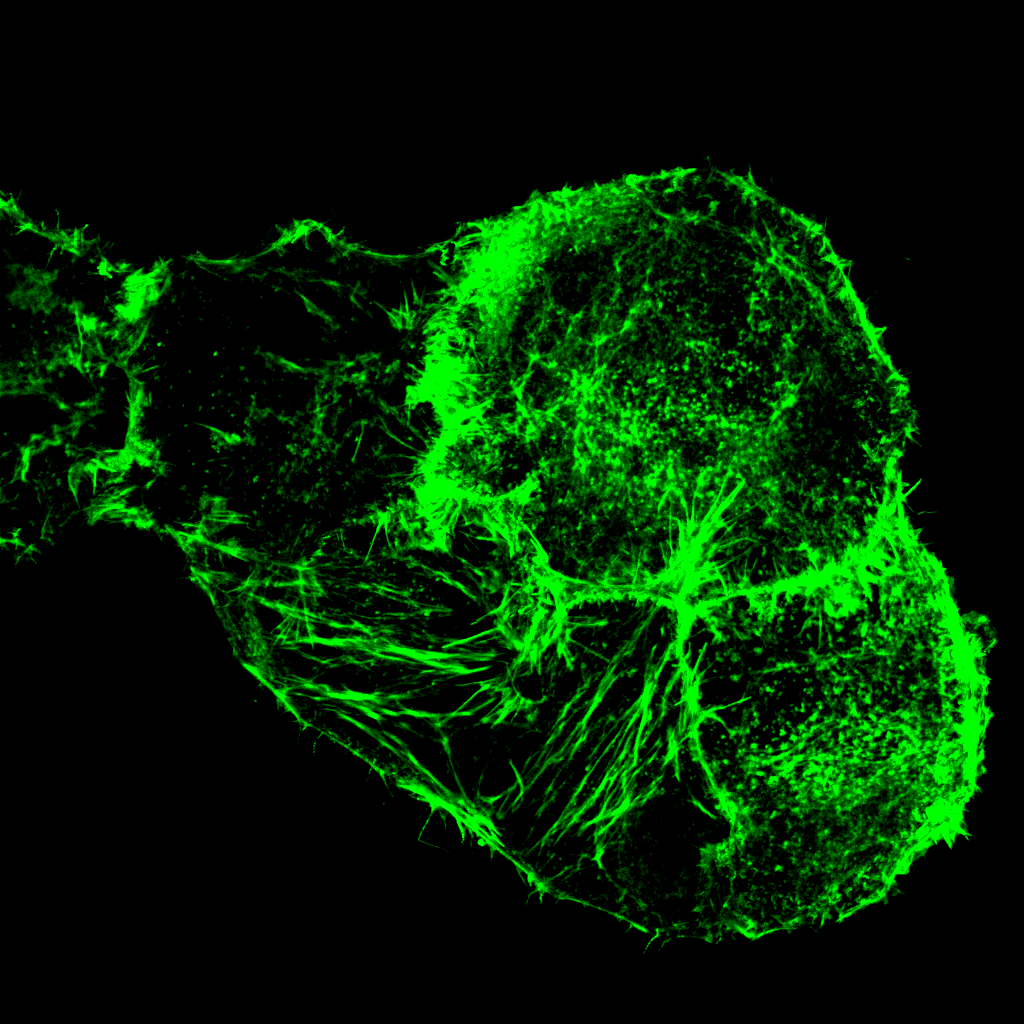

Supplement: Supplementary file 12 — Source data Fig. 3 [file 44320_2024_32_MOESM12_ESM.zip › Figure 3/Figure 3C/shp53_1_60x_factin.tif]

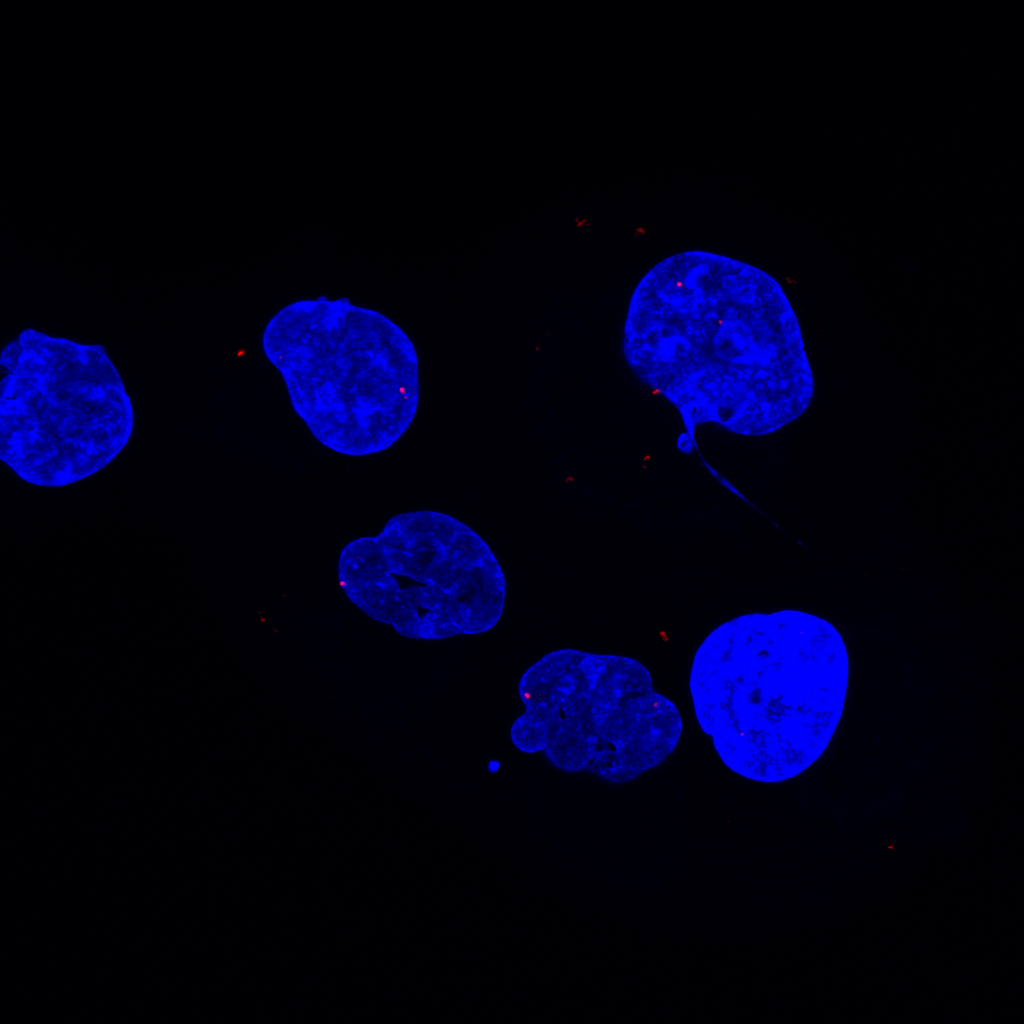

Supplement: Supplementary file 12 — Source data Fig. 3 [file 44320_2024_32_MOESM12_ESM.zip › Figure 3/Figure 3C/shp53_1_60x_red_dapi.tif]

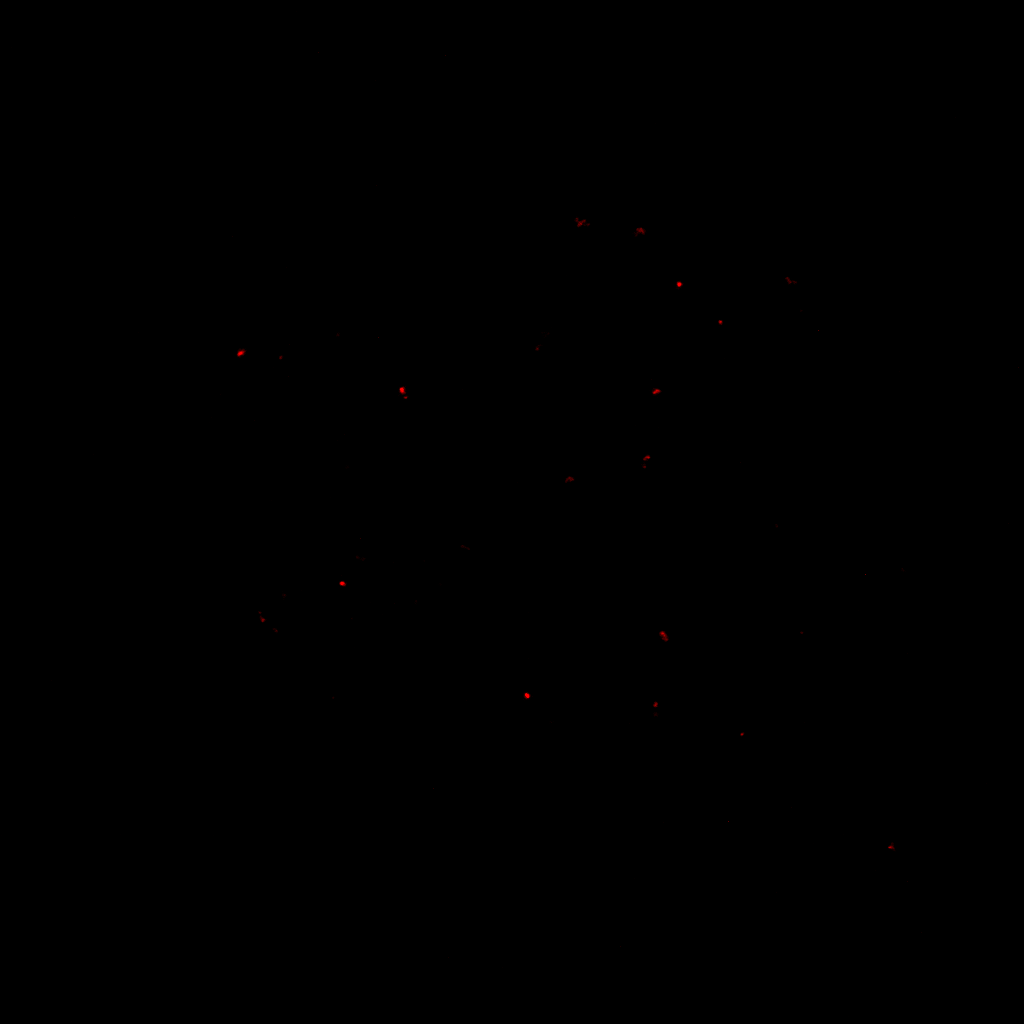

Supplement: Supplementary file 12 — Source data Fig. 3 [file 44320_2024_32_MOESM12_ESM.zip › Figure 3/Figure 3C/shp53_1_red_60x.tif]

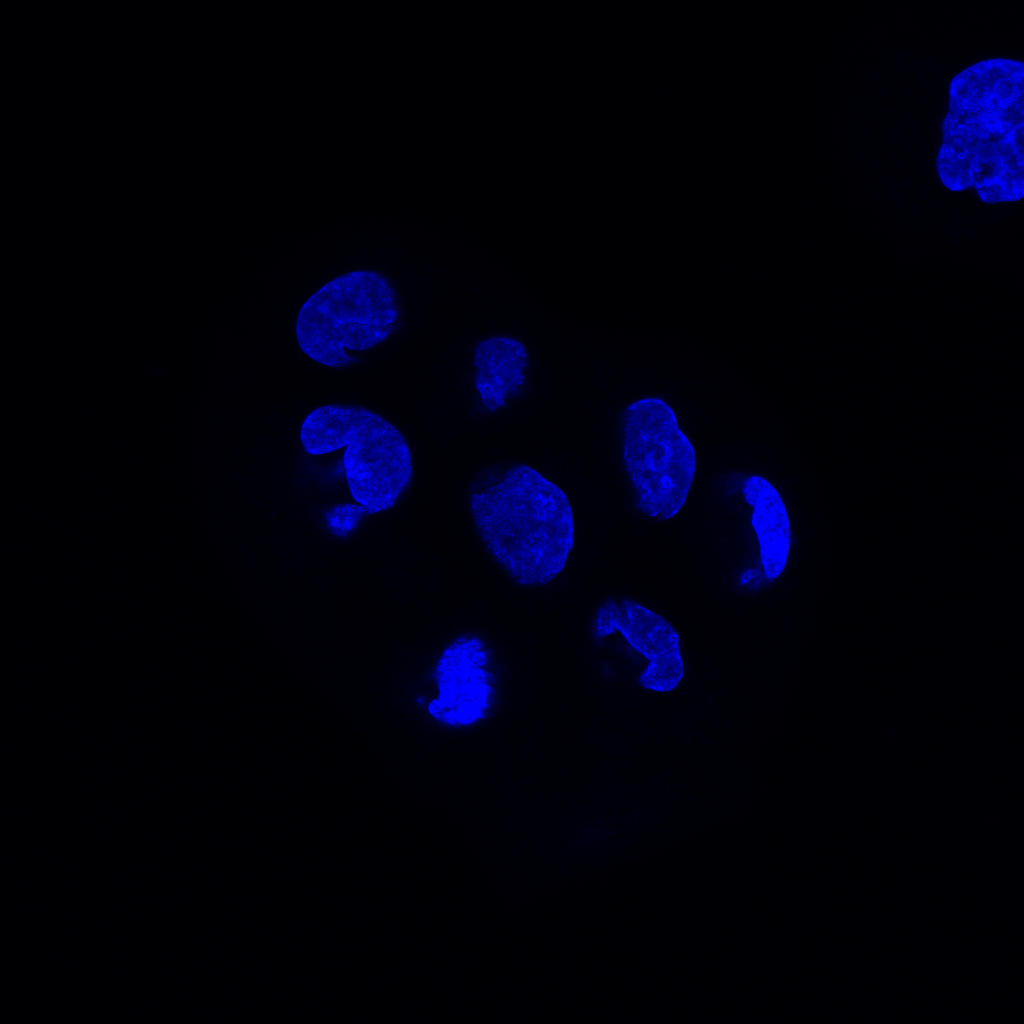

Supplement: Supplementary file 12 — Source data Fig. 3 [file 44320_2024_32_MOESM12_ESM.zip › Figure 3/Figure 3C/shp53_2_60x_dapi.tif]

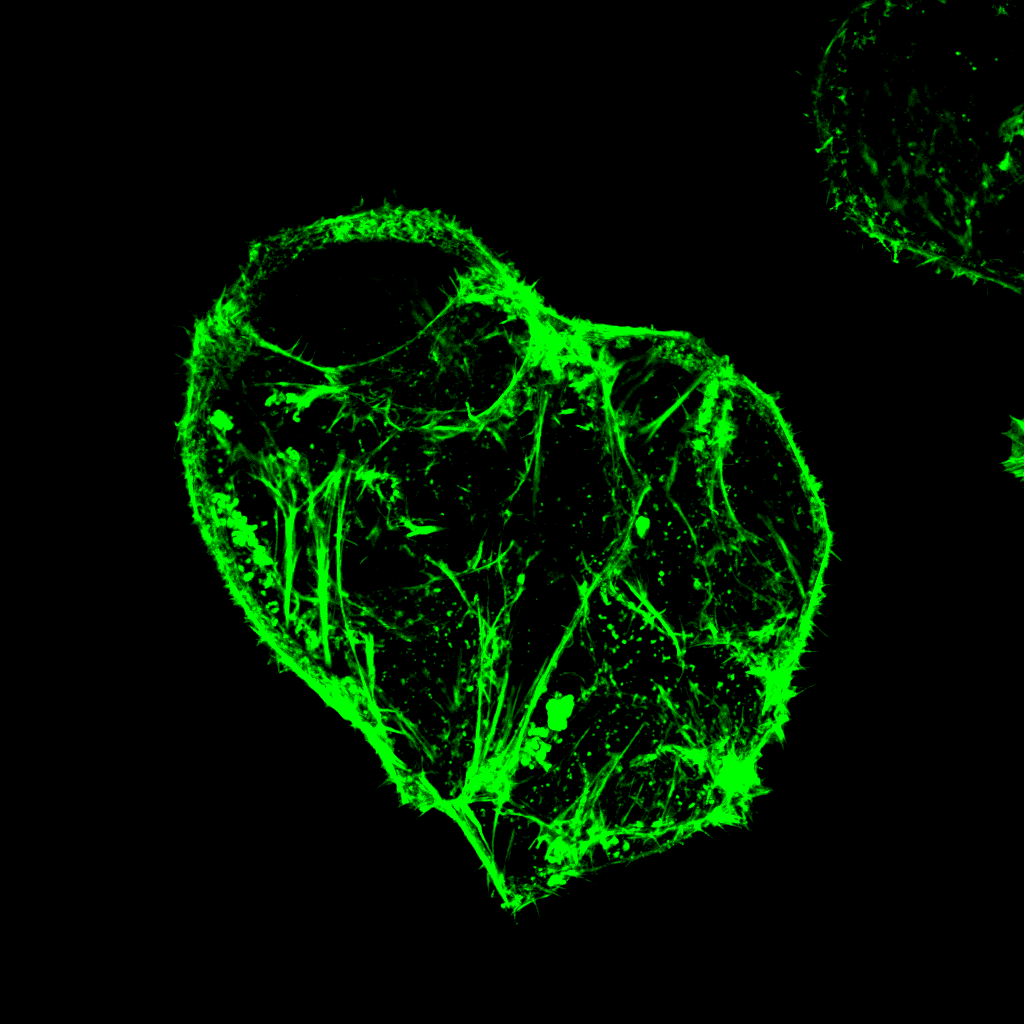

Supplement: Supplementary file 12 — Source data Fig. 3 [file 44320_2024_32_MOESM12_ESM.zip › Figure 3/Figure 3C/shp53_2_60x_factin.tif]

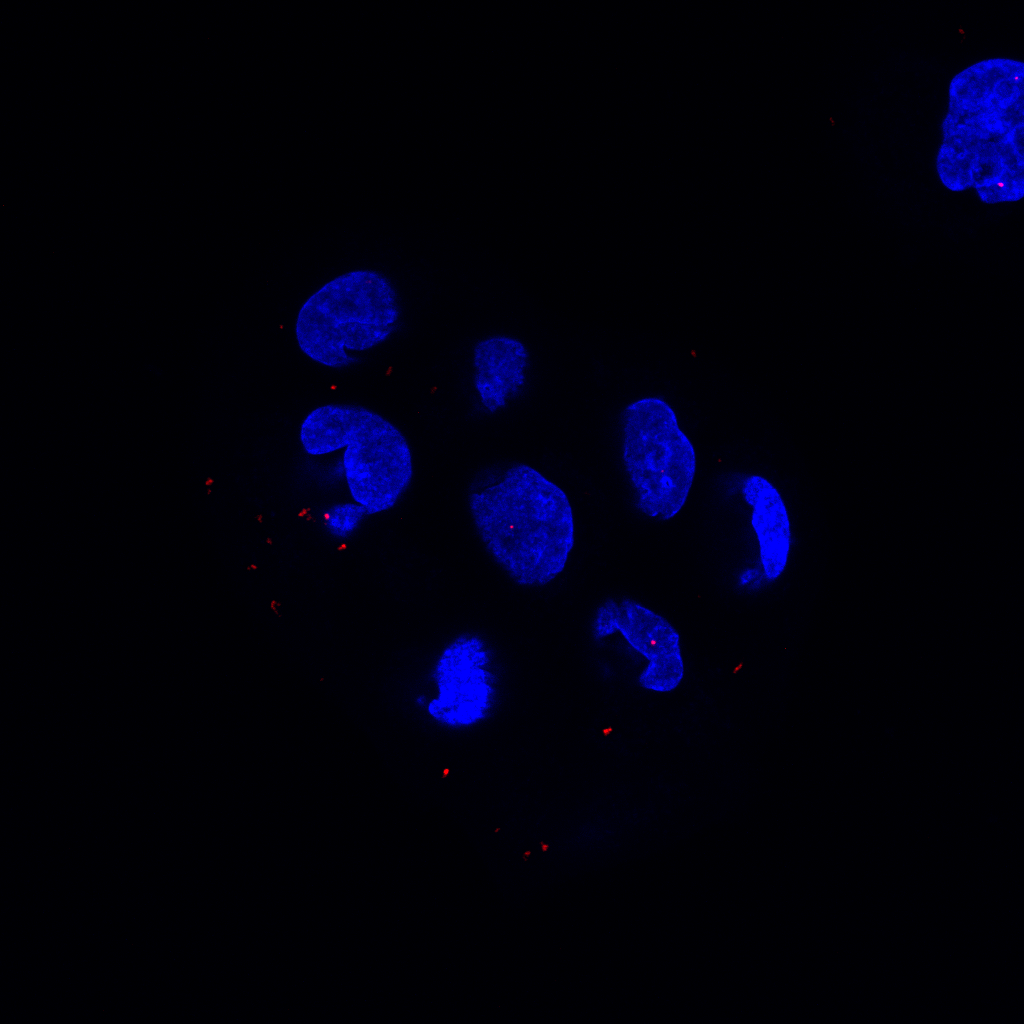

Supplement: Supplementary file 12 — Source data Fig. 3 [file 44320_2024_32_MOESM12_ESM.zip › Figure 3/Figure 3C/shp53_2_60x_red_dapi.tif]

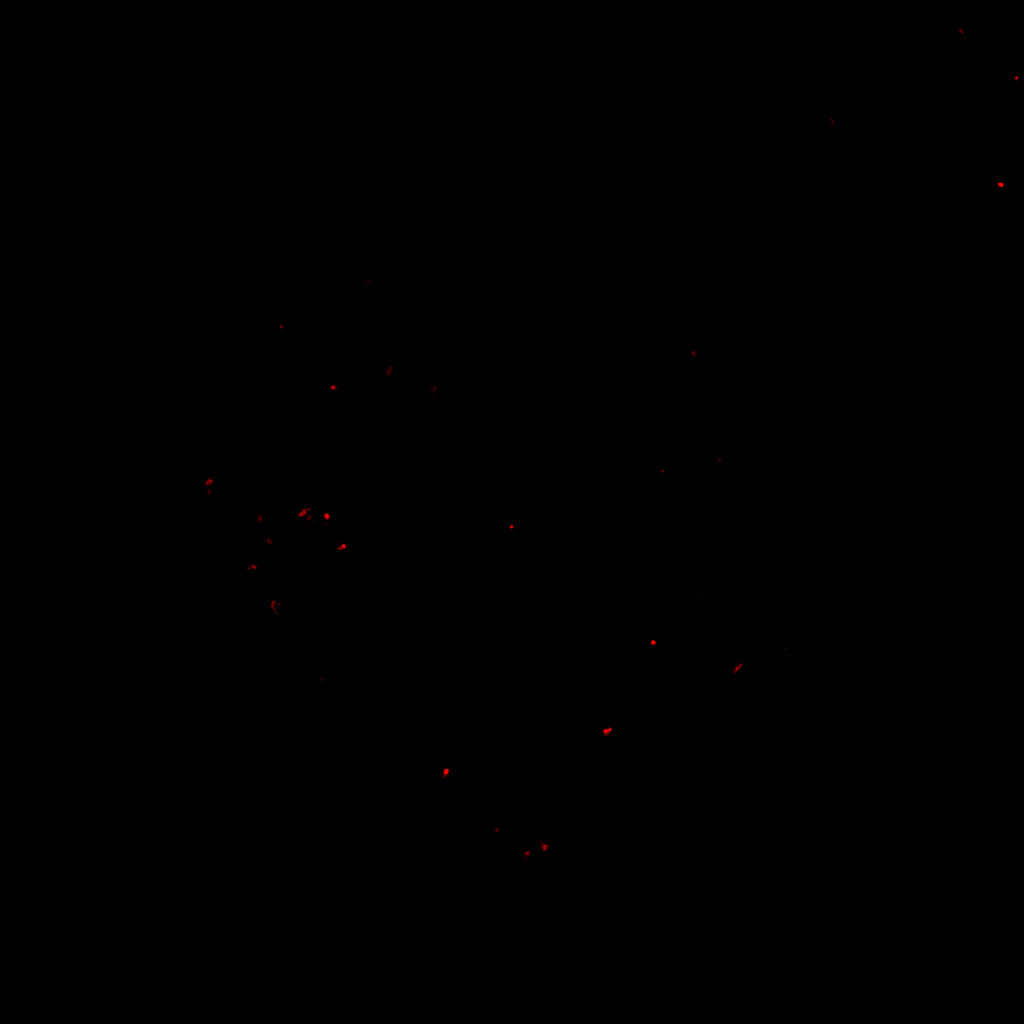

Supplement: Supplementary file 12 — Source data Fig. 3 [file 44320_2024_32_MOESM12_ESM.zip › Figure 3/Figure 3C/shp53_2_red_60x.tif]

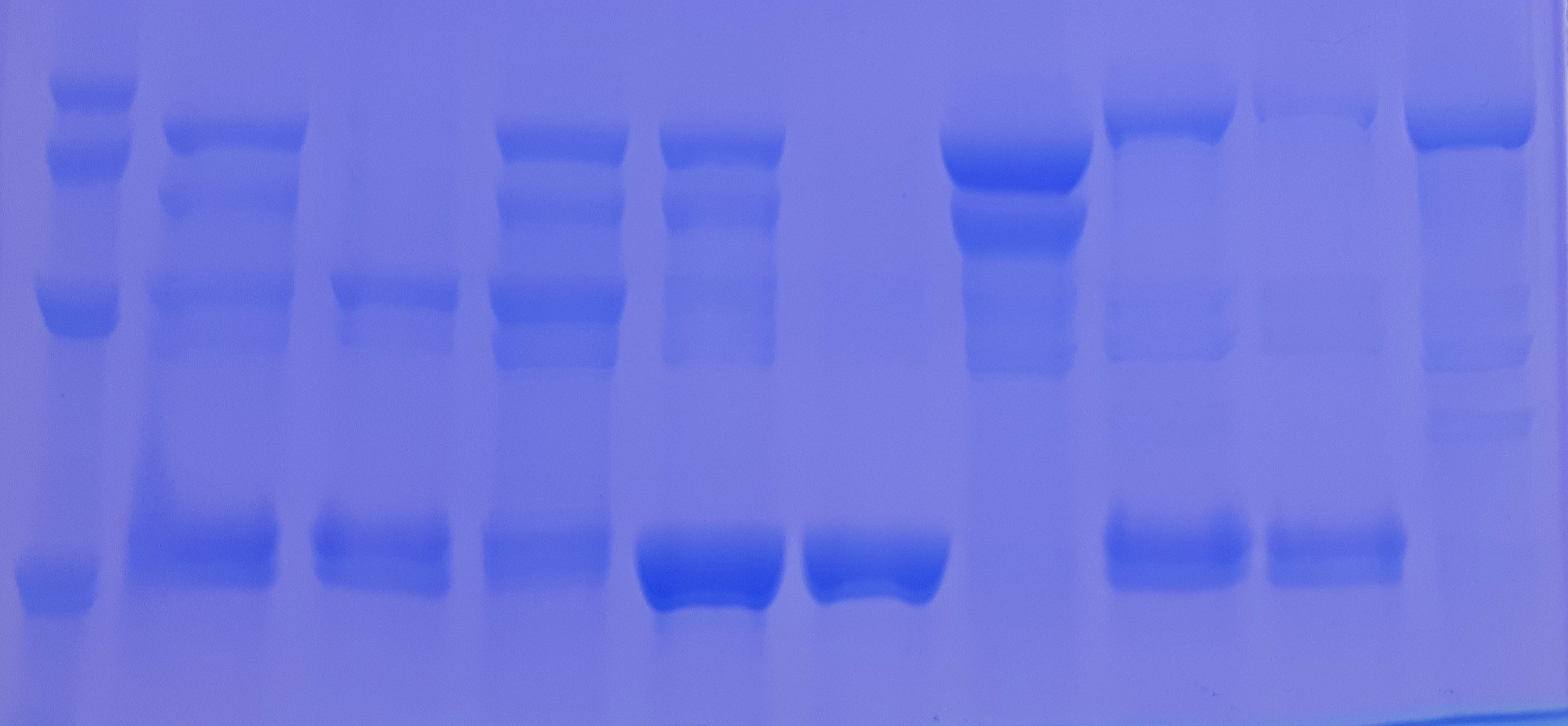

Supplement: Supplementary file 12 — Source data Fig. 3 [file 44320_2024_32_MOESM12_ESM.zip › Figure 3/Figure 3D/Coomassie-Stain-Figure 3D.tif]

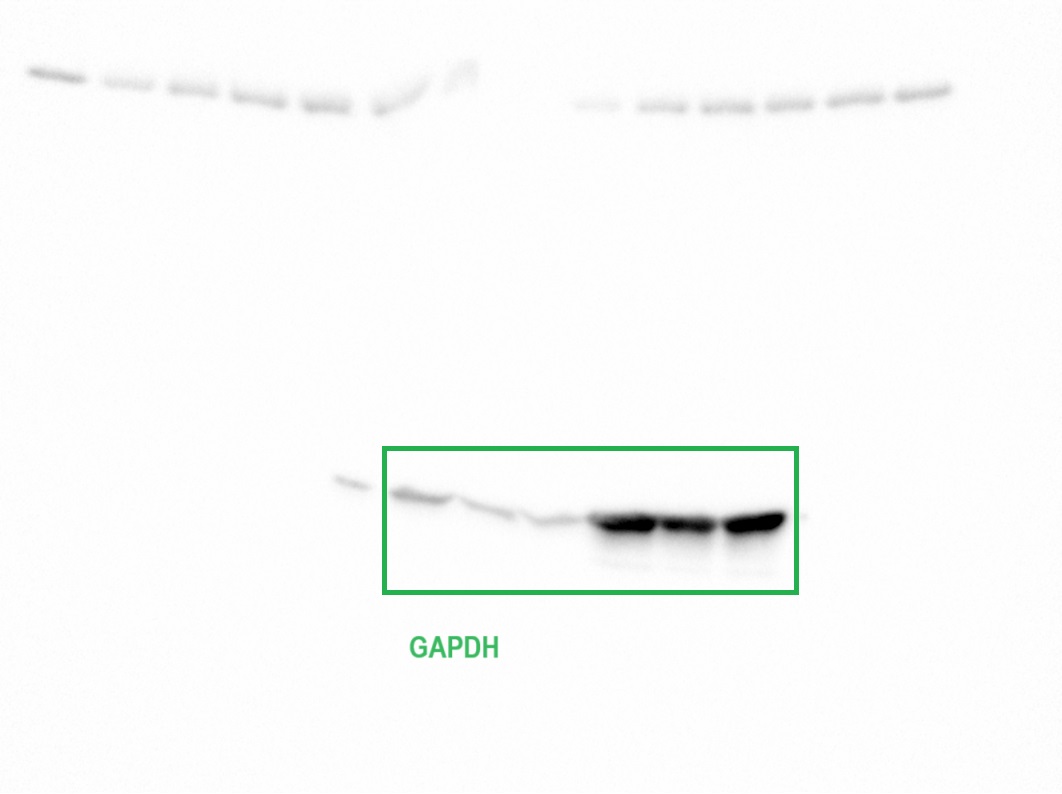

Supplement: Supplementary file 12 — Source data Fig. 3 [file 44320_2024_32_MOESM12_ESM.zip › Figure 3/Figure 3G/Annotated/GAPDH-annotated.jpg]

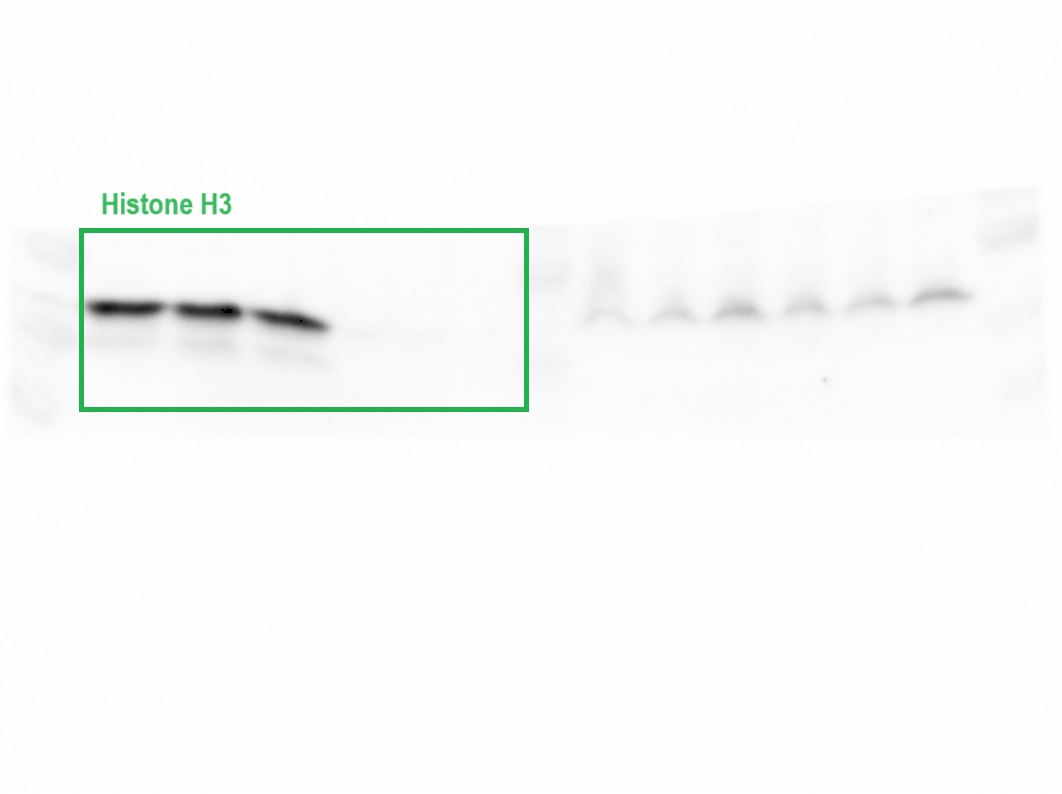

Supplement: Supplementary file 12 — Source data Fig. 3 [file 44320_2024_32_MOESM12_ESM.zip › Figure 3/Figure 3G/Annotated/Histone H3-annotated.jpg]

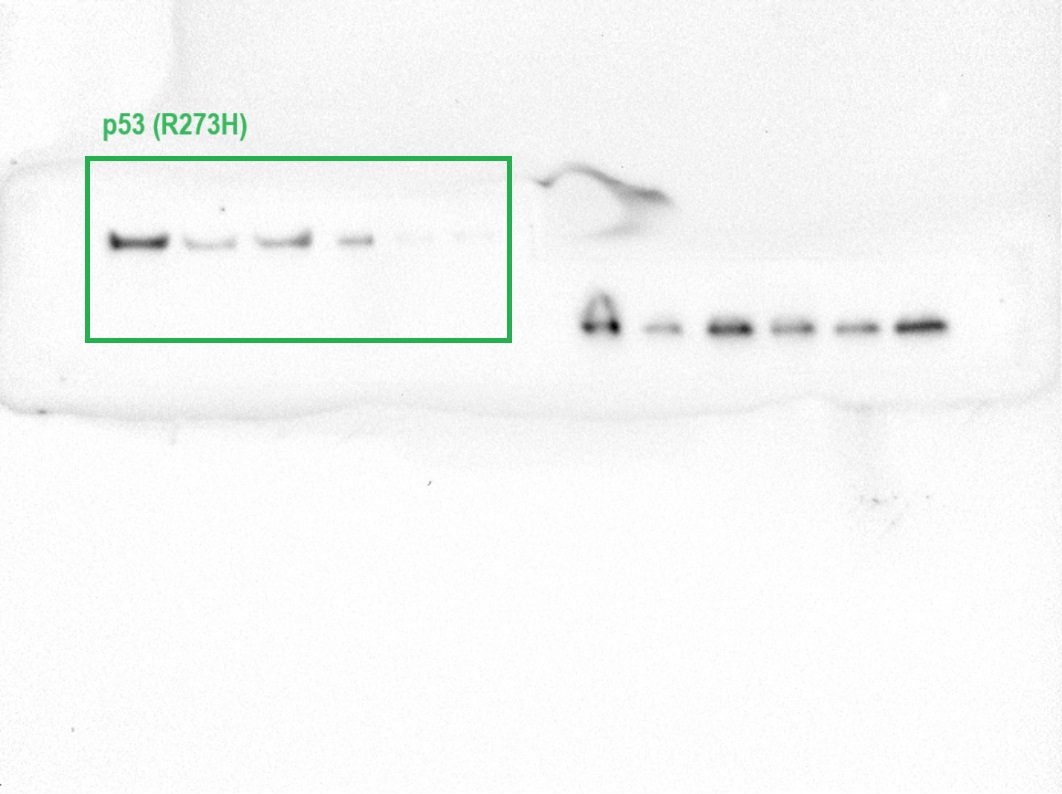

Supplement: Supplementary file 12 — Source data Fig. 3 [file 44320_2024_32_MOESM12_ESM.zip › Figure 3/Figure 3G/Annotated/p53-annotated.jpg]

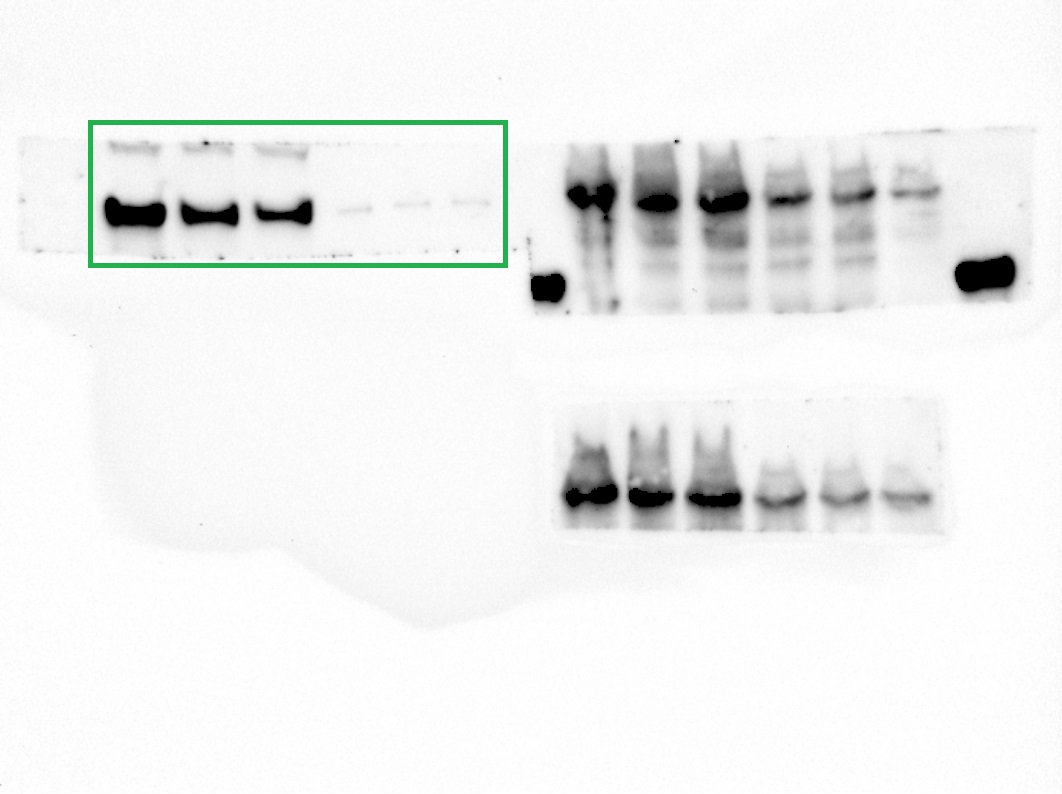

Supplement: Supplementary file 12 — Source data Fig. 3 [file 44320_2024_32_MOESM12_ESM.zip › Figure 3/Figure 3G/Annotated/USP28-LongerExposure-annotated.jpg]

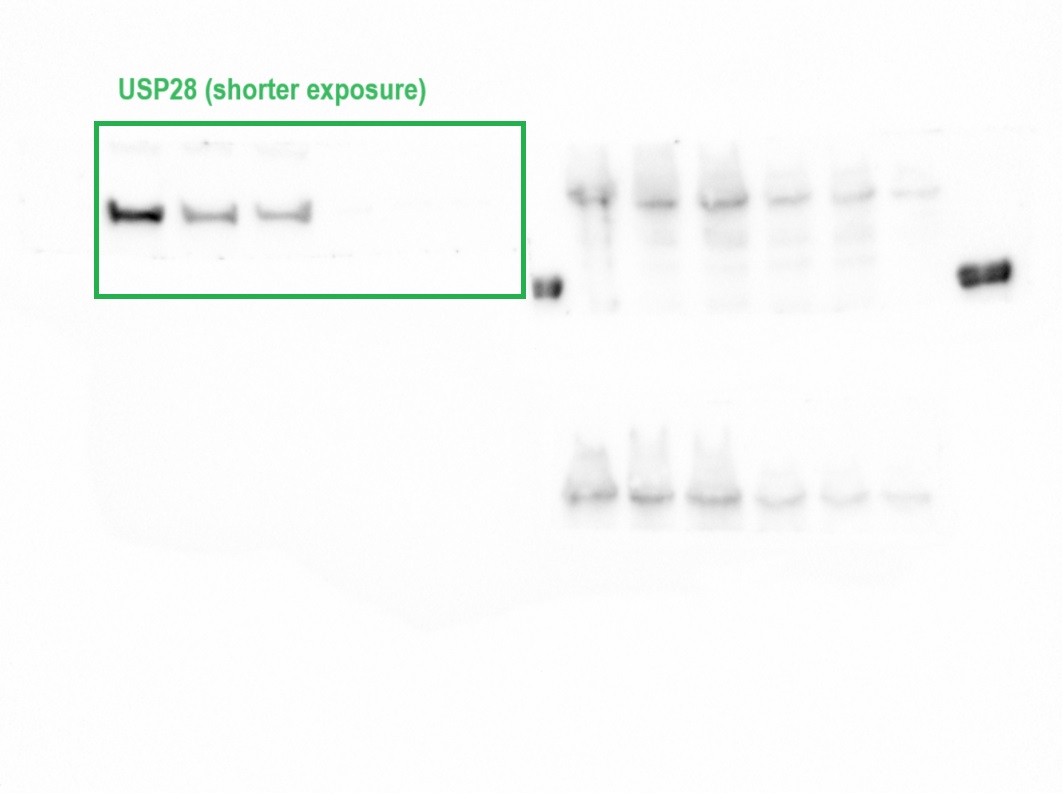

Supplement: Supplementary file 12 — Source data Fig. 3 [file 44320_2024_32_MOESM12_ESM.zip › Figure 3/Figure 3G/Annotated/USP28-ShorterExposure-annotated.jpg]

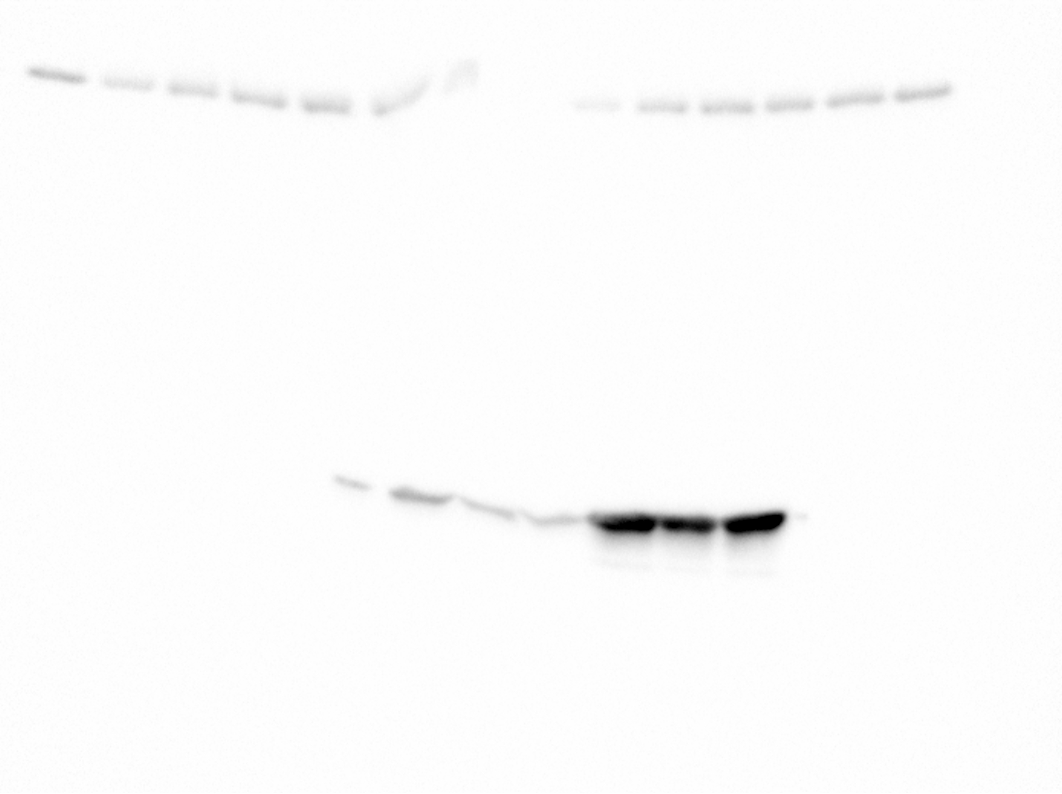

Supplement: Supplementary file 12 — Source data Fig. 3 [file 44320_2024_32_MOESM12_ESM.zip › Figure 3/Figure 3G/Non-annotated/GAPDH.jpg]

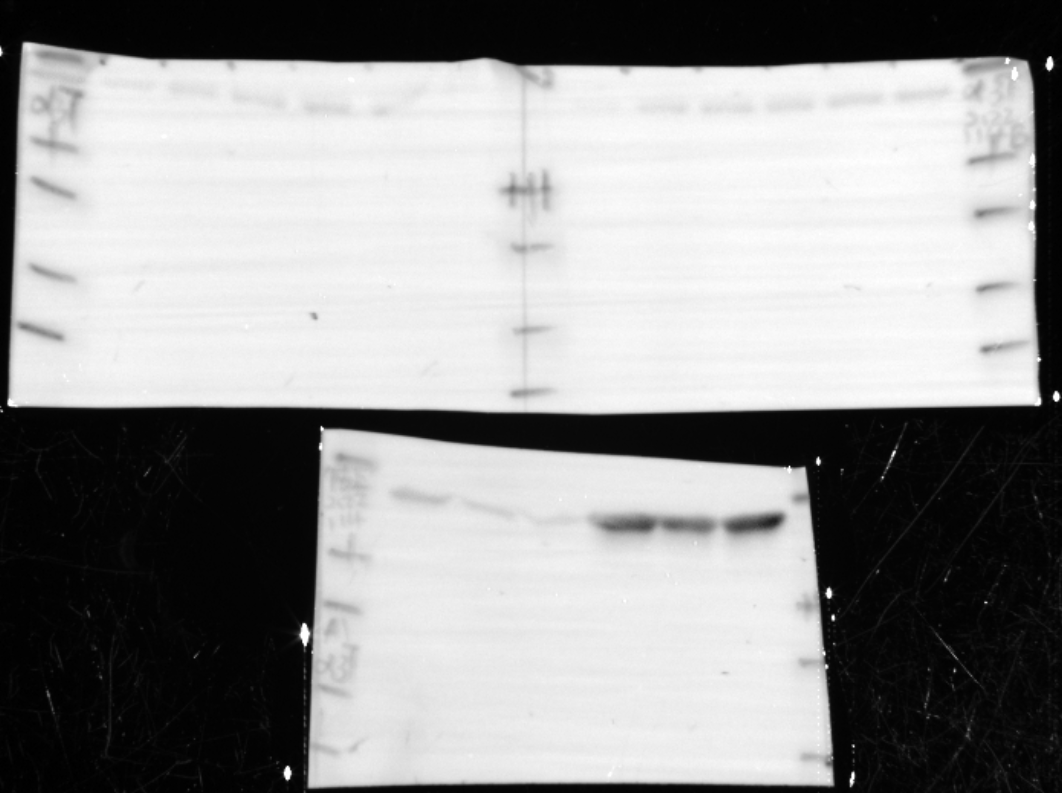

Supplement: Supplementary file 12 — Source data Fig. 3 [file 44320_2024_32_MOESM12_ESM.zip › Figure 3/Figure 3G/Non-annotated/GAPDH-merged.tif]

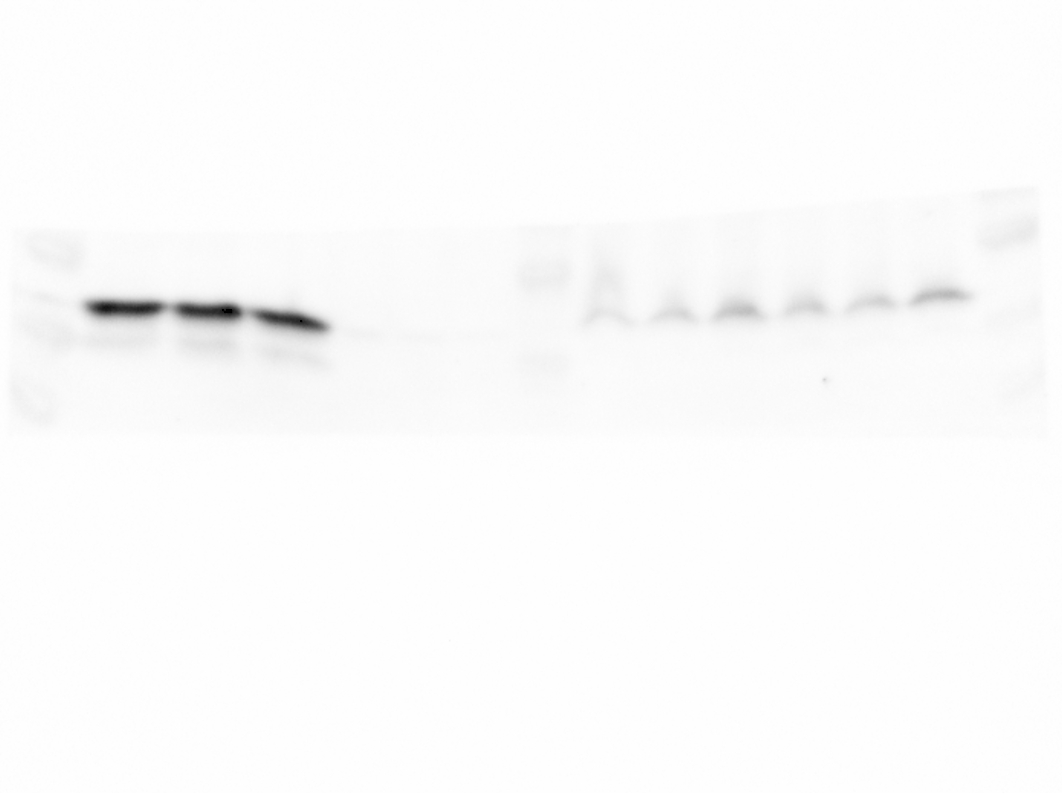

Supplement: Supplementary file 12 — Source data Fig. 3 [file 44320_2024_32_MOESM12_ESM.zip › Figure 3/Figure 3G/Non-annotated/Histone H3.jpg]

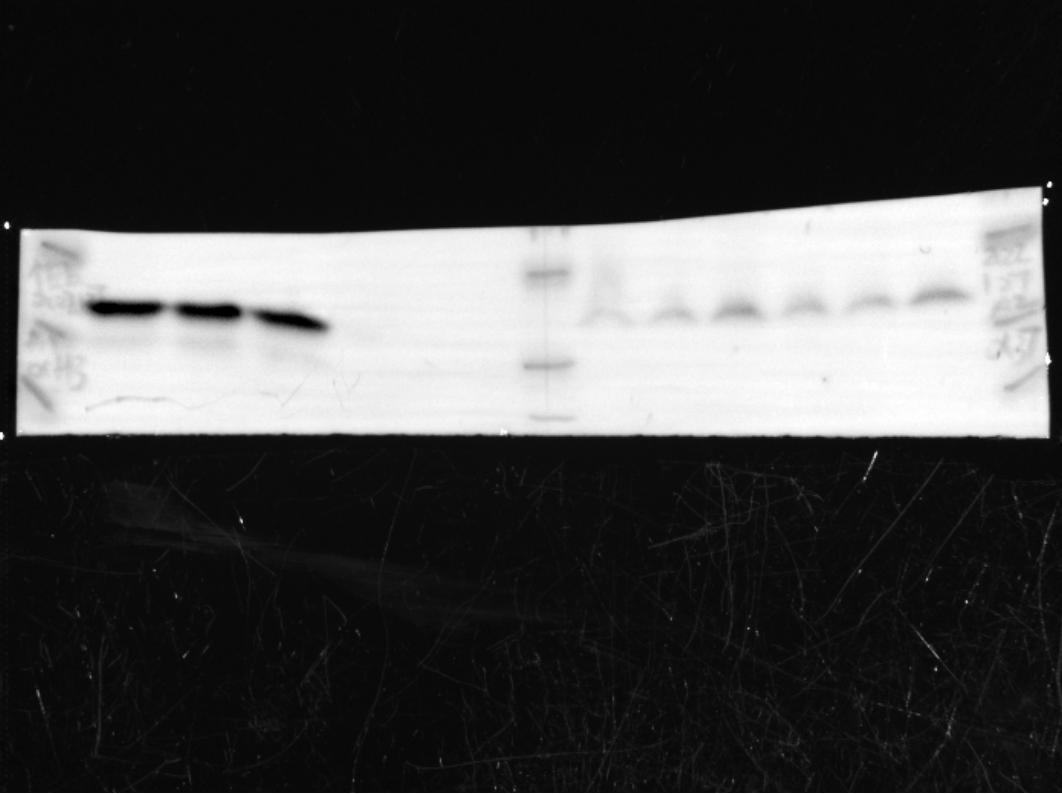

Supplement: Supplementary file 12 — Source data Fig. 3 [file 44320_2024_32_MOESM12_ESM.zip › Figure 3/Figure 3G/Non-annotated/Histone H3-merged.tif]

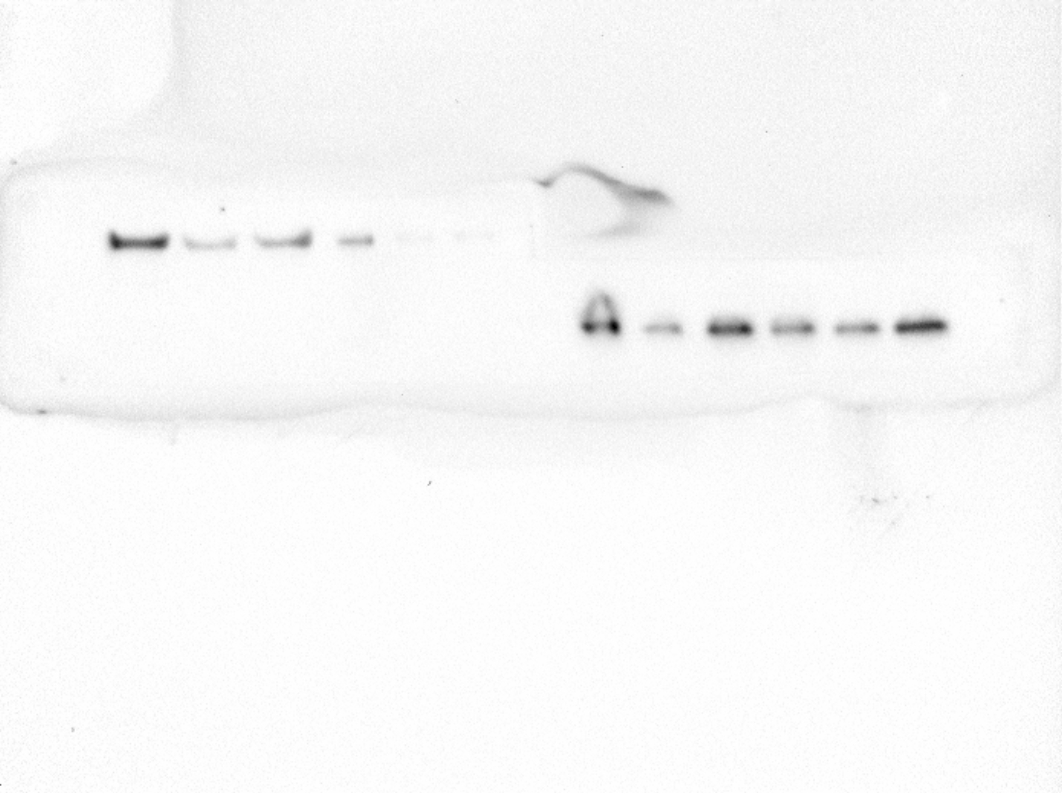

Supplement: Supplementary file 12 — Source data Fig. 3 [file 44320_2024_32_MOESM12_ESM.zip › Figure 3/Figure 3G/Non-annotated/p53.jpg]

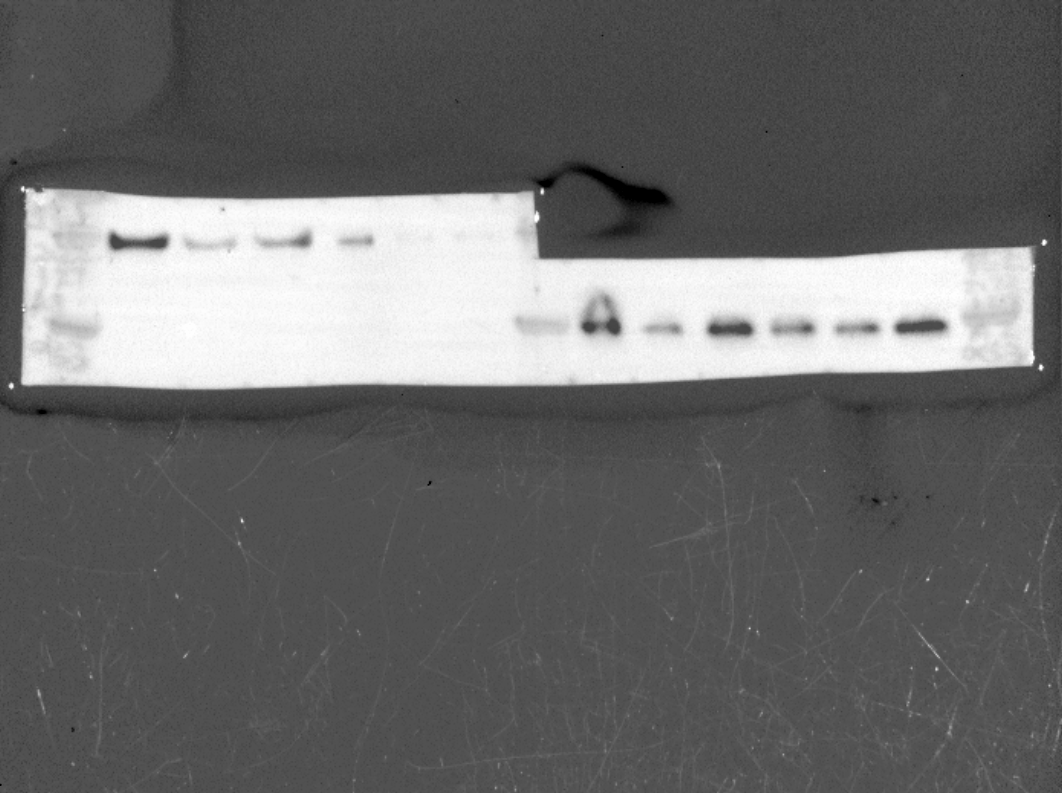

Supplement: Supplementary file 12 — Source data Fig. 3 [file 44320_2024_32_MOESM12_ESM.zip › Figure 3/Figure 3G/Non-annotated/p53-merged.tif]

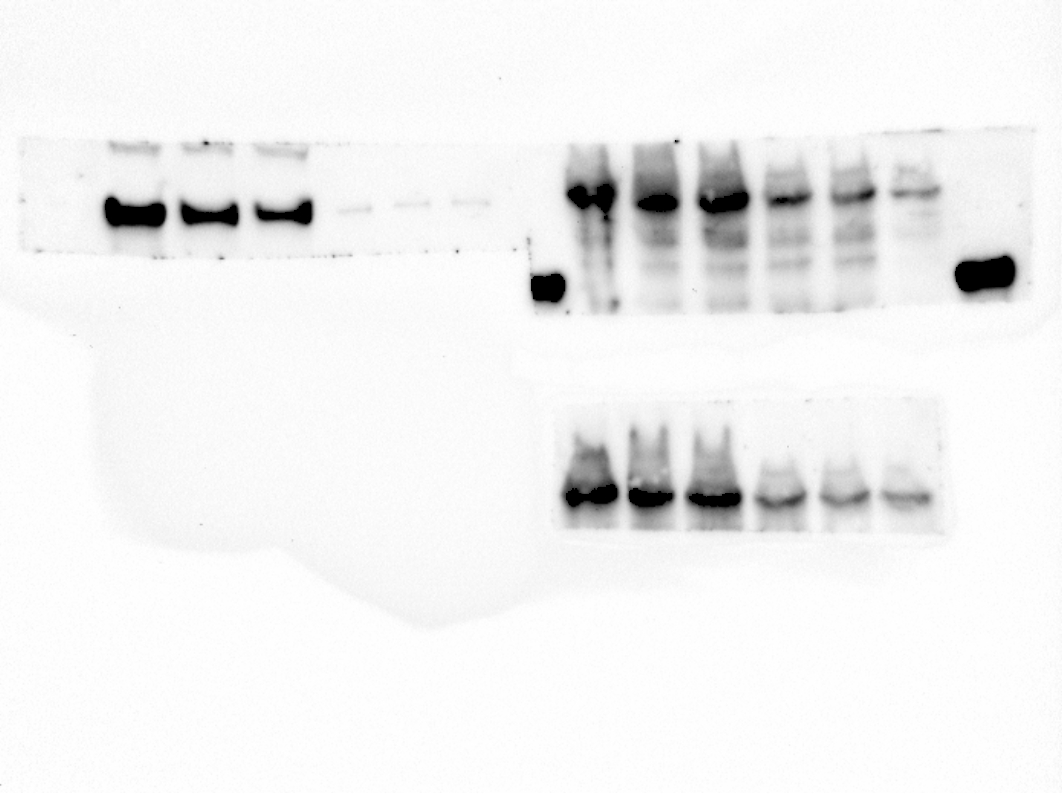

Supplement: Supplementary file 12 — Source data Fig. 3 [file 44320_2024_32_MOESM12_ESM.zip › Figure 3/Figure 3G/Non-annotated/USP28-LongerExposure.jpg]

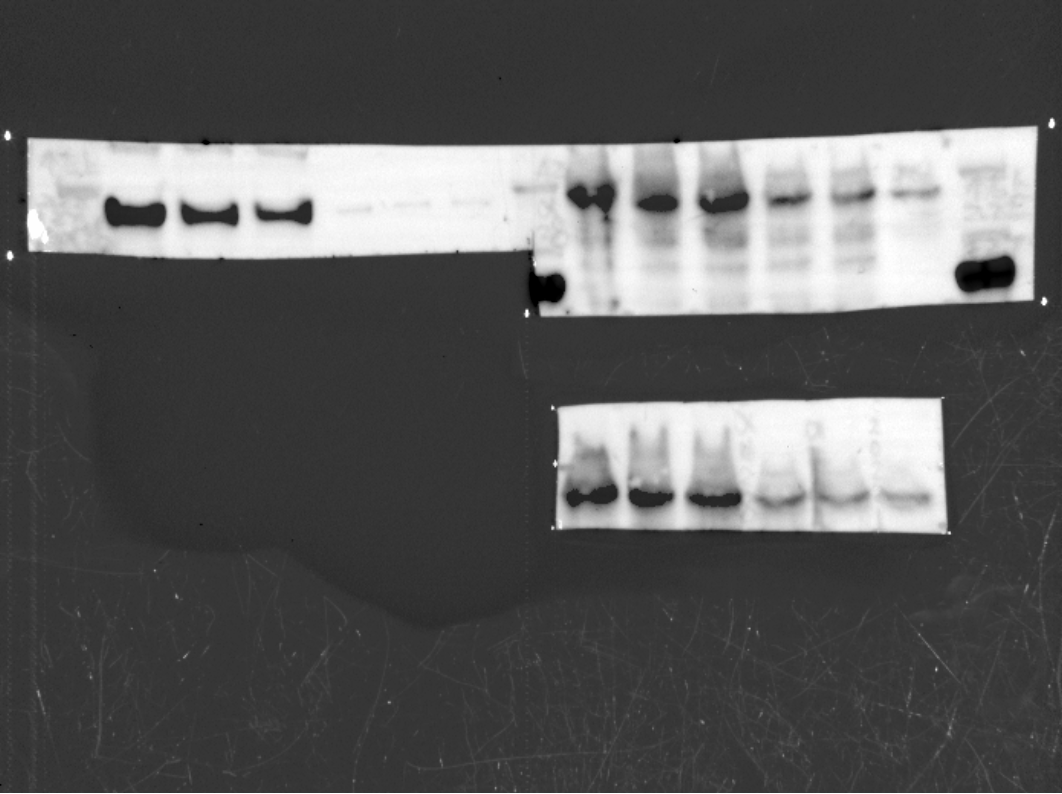

Supplement: Supplementary file 12 — Source data Fig. 3 [file 44320_2024_32_MOESM12_ESM.zip › Figure 3/Figure 3G/Non-annotated/USP28-longer-merged.tif]

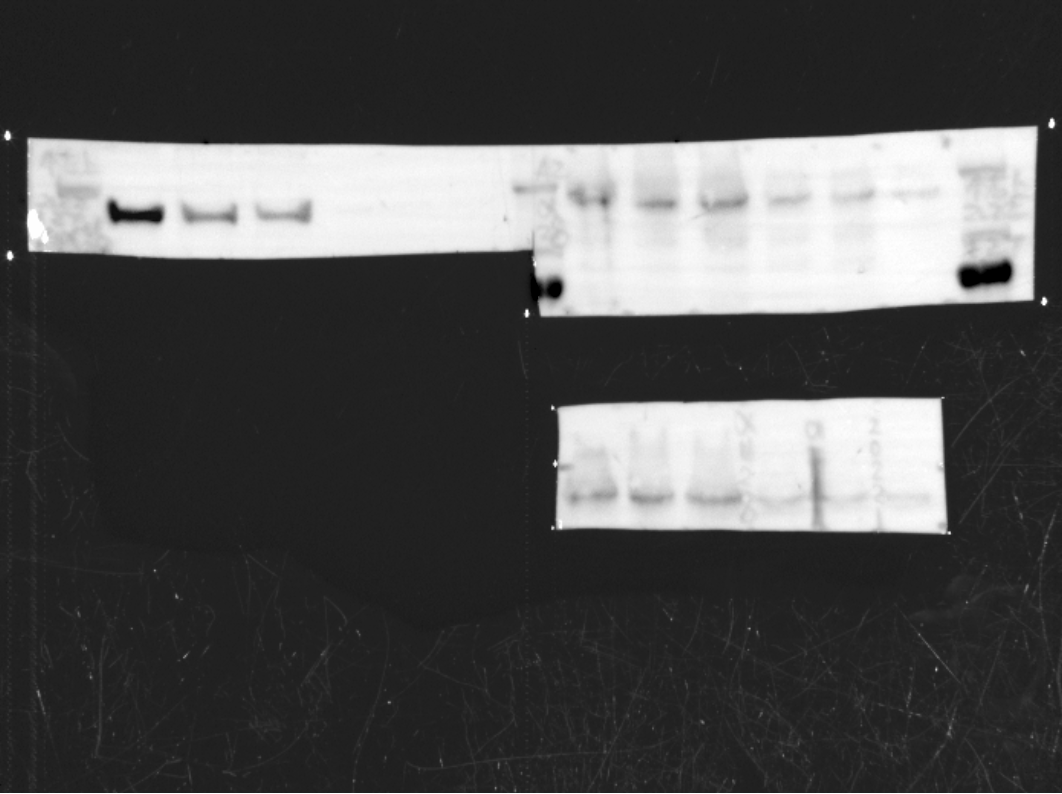

Supplement: Supplementary file 12 — Source data Fig. 3 [file 44320_2024_32_MOESM12_ESM.zip › Figure 3/Figure 3G/Non-annotated/USP28-merged.tif]

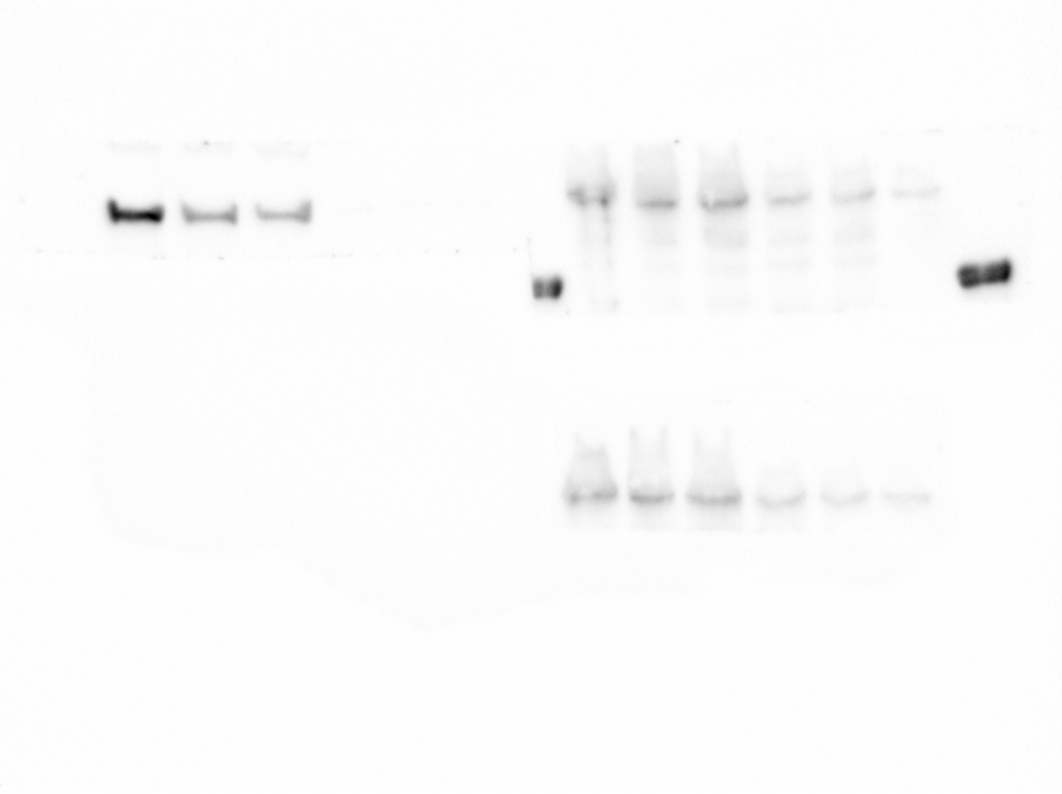

Supplement: Supplementary file 12 — Source data Fig. 3 [file 44320_2024_32_MOESM12_ESM.zip › Figure 3/Figure 3G/Non-annotated/USP28-ShorterExposure.jpg]

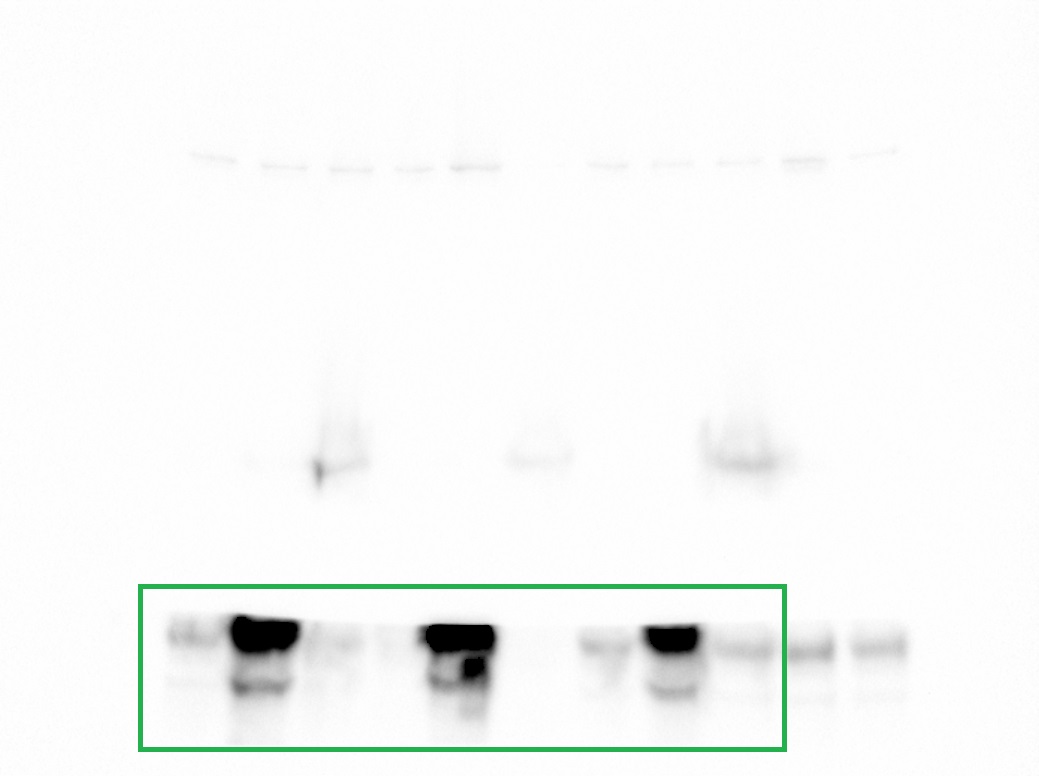

Supplement: Supplementary file 12 — Source data Fig. 3 [file 44320_2024_32_MOESM12_ESM.zip › Figure 3/Figure 3H/Annotated/CCDC6-annotated.jpg]

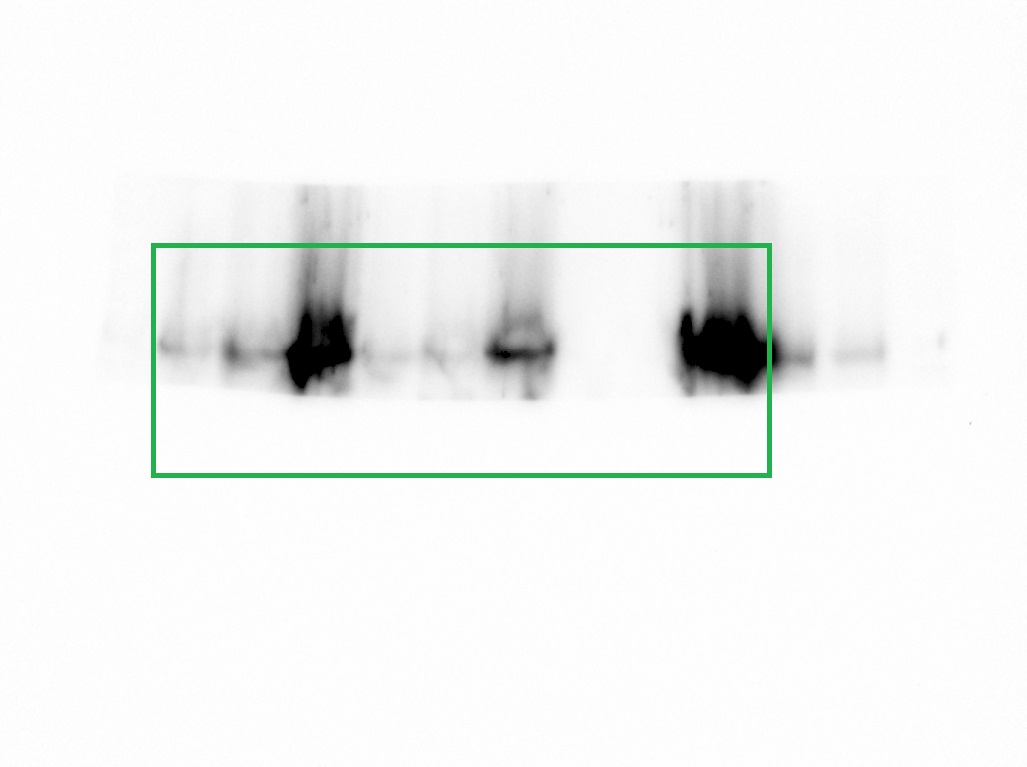

Supplement: Supplementary file 12 — Source data Fig. 3 [file 44320_2024_32_MOESM12_ESM.zip › Figure 3/Figure 3H/Annotated/FBXO42-annotated.jpg]

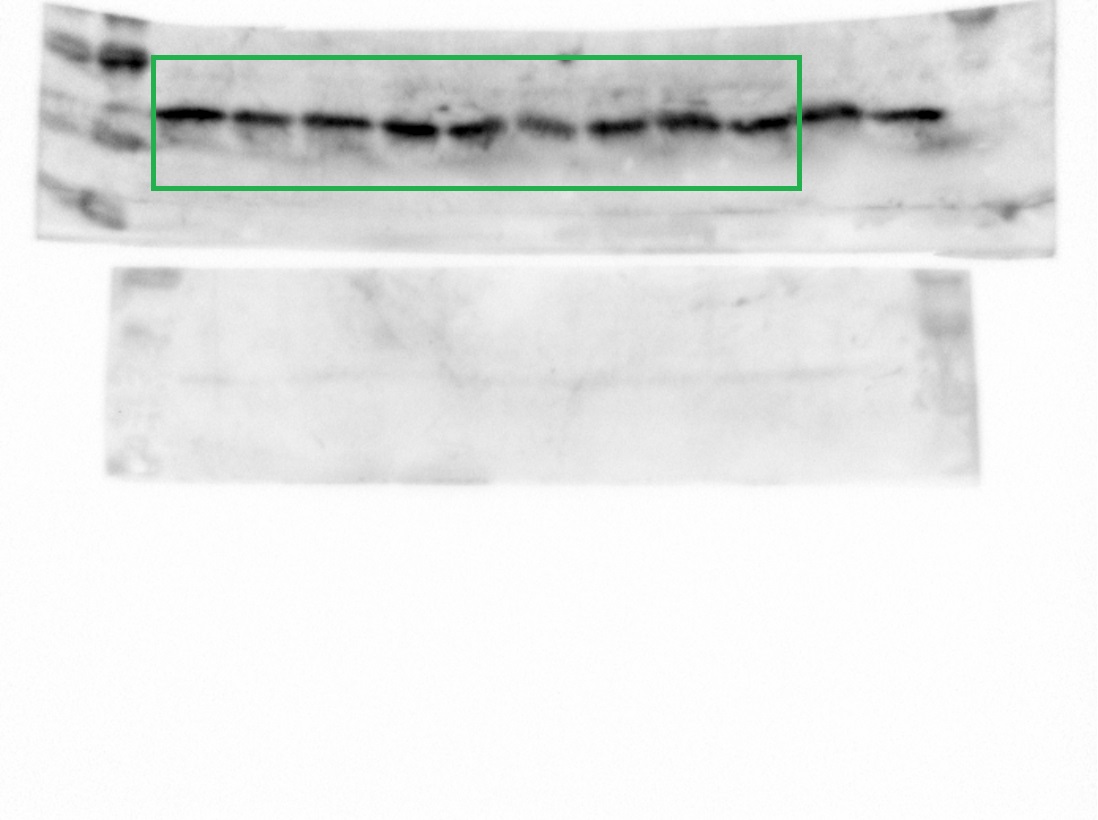

Supplement: Supplementary file 12 — Source data Fig. 3 [file 44320_2024_32_MOESM12_ESM.zip › Figure 3/Figure 3H/Annotated/H3-annotated.jpg]

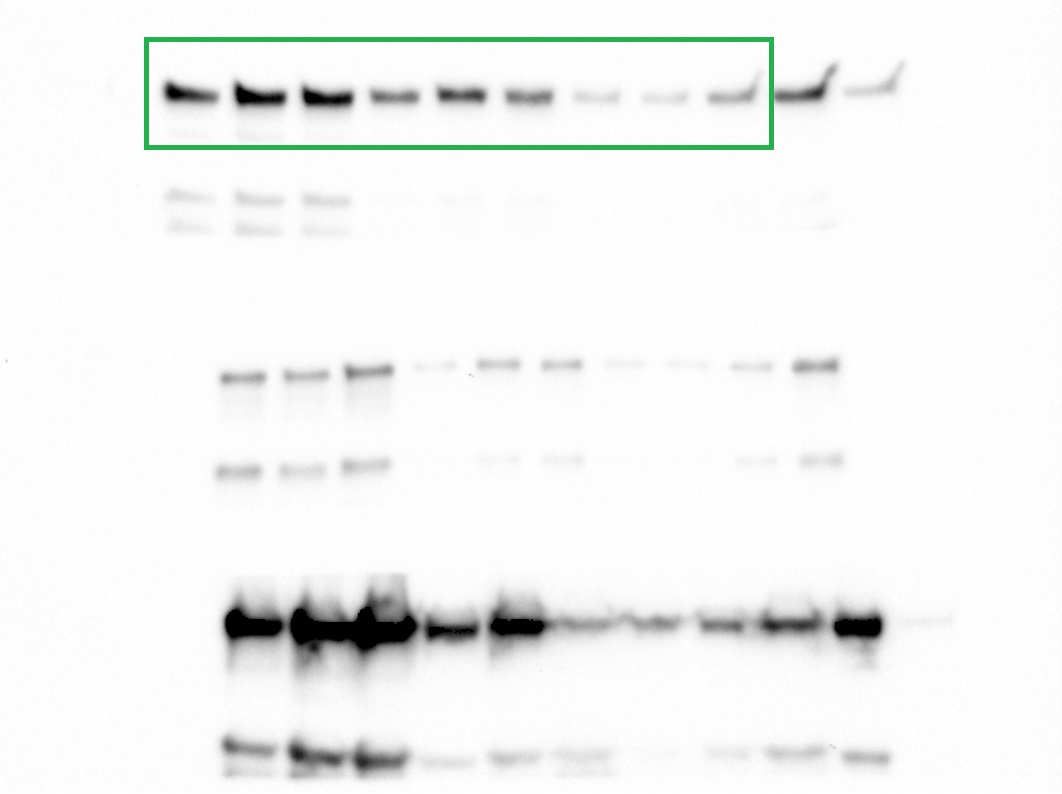

Supplement: Supplementary file 12 — Source data Fig. 3 [file 44320_2024_32_MOESM12_ESM.zip › Figure 3/Figure 3H/Annotated/p53DO1-annotated.jpg]

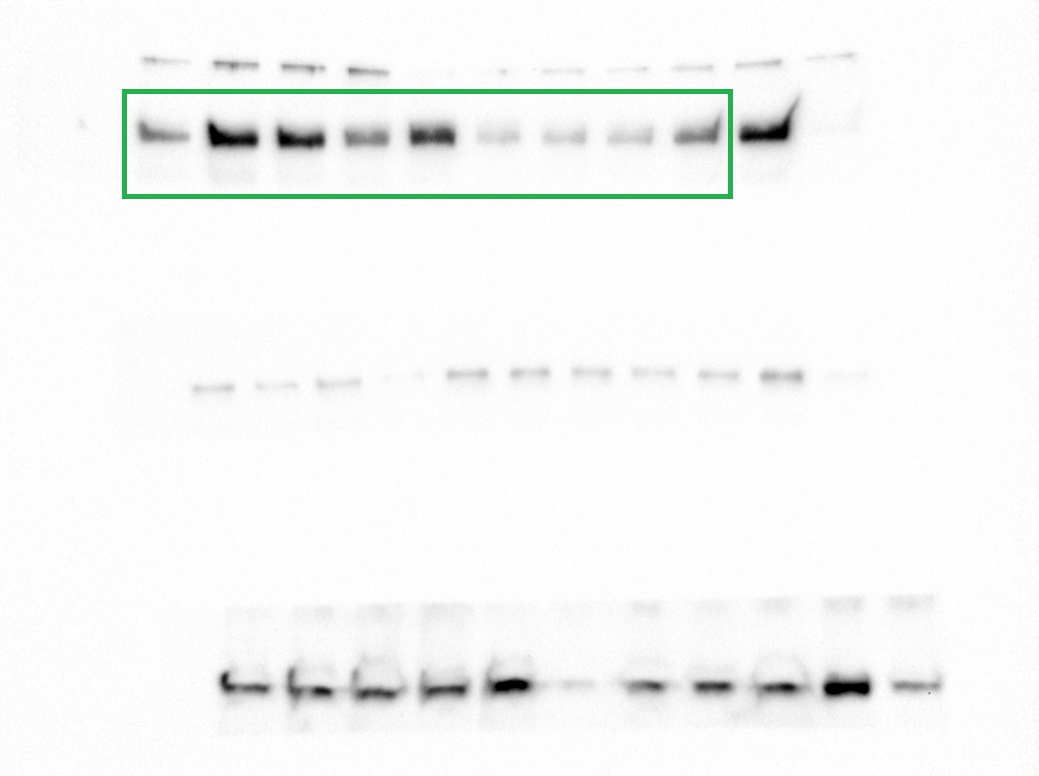

Supplement: Supplementary file 12 — Source data Fig. 3 [file 44320_2024_32_MOESM12_ESM.zip › Figure 3/Figure 3H/Annotated/USP28-annotated.jpg]

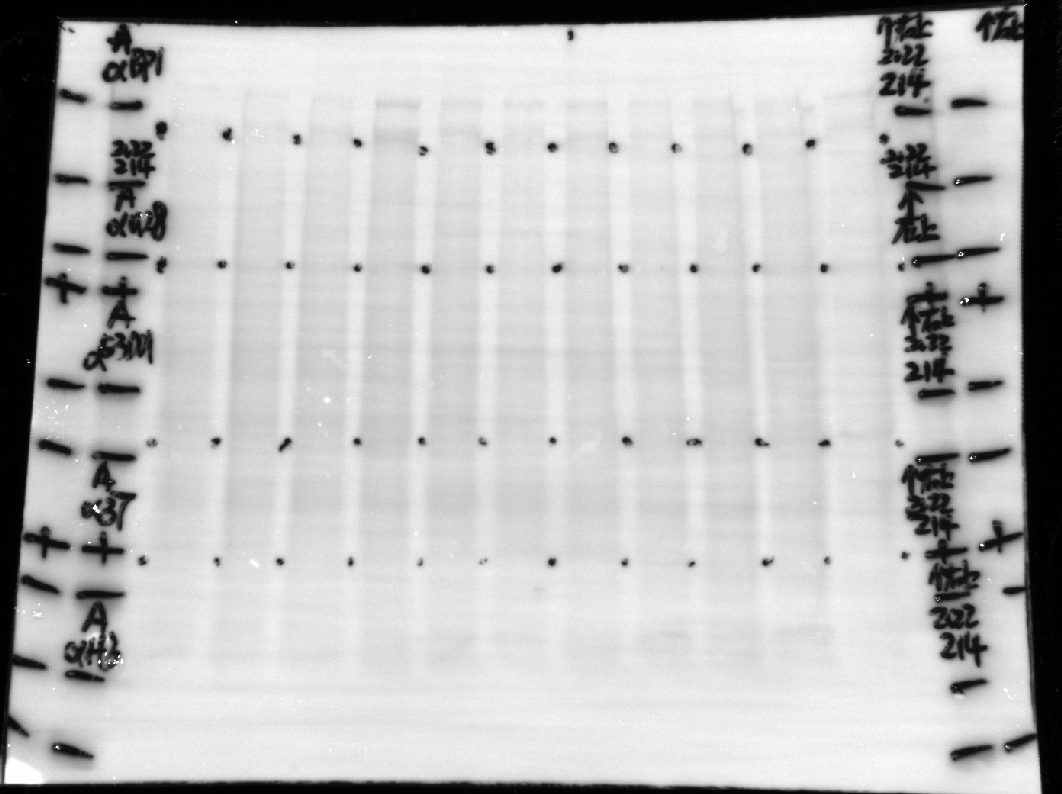

Supplement: Supplementary file 12 — Source data Fig. 3 [file 44320_2024_32_MOESM12_ESM.zip › Figure 3/Figure 3H/Figure 3H-MembraneCutting.tif]

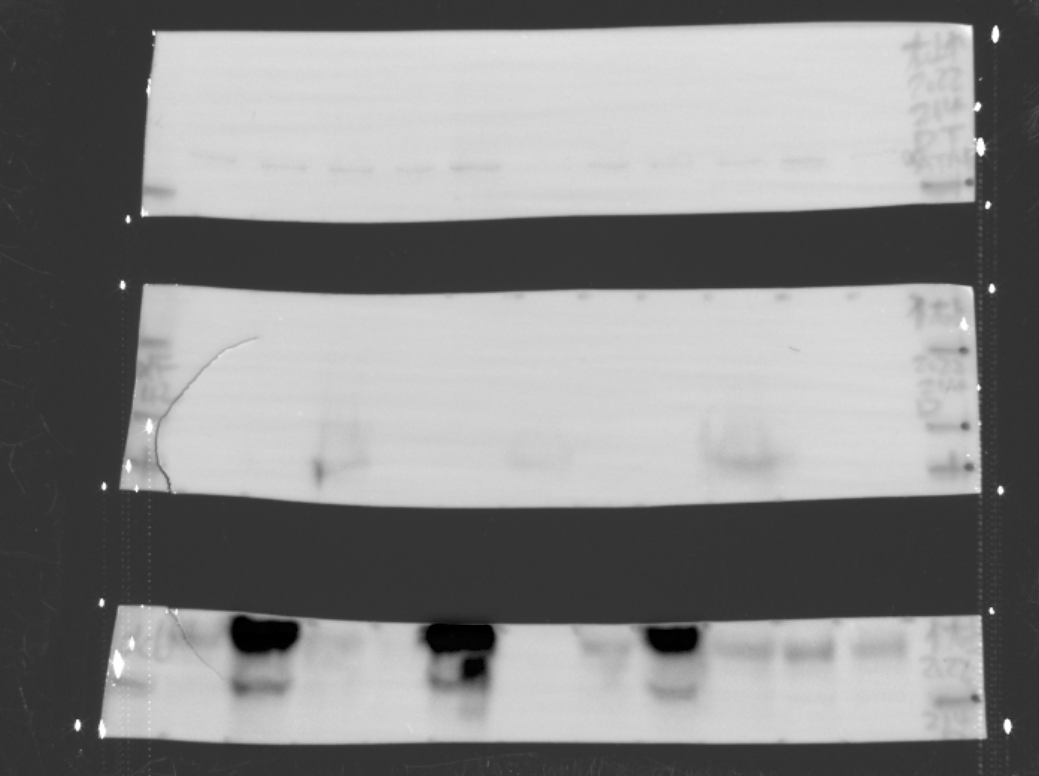

Supplement: Supplementary file 12 — Source data Fig. 3 [file 44320_2024_32_MOESM12_ESM.zip › Figure 3/Figure 3H/Figure-3H-CCDC6-merged.tif]

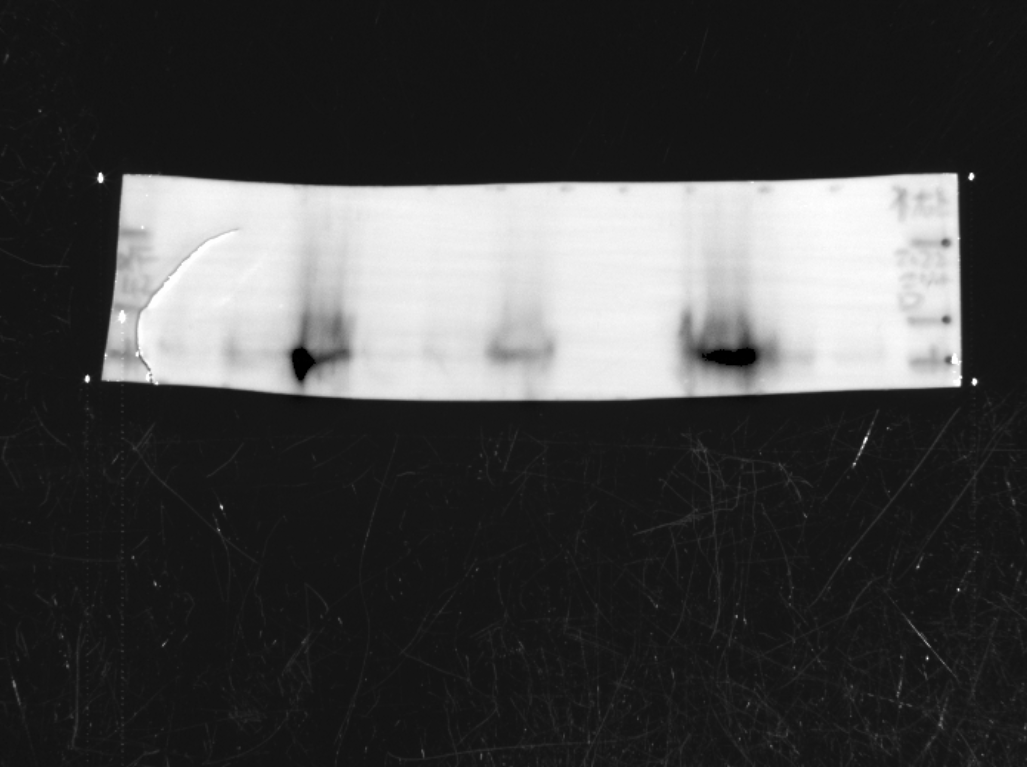

Supplement: Supplementary file 12 — Source data Fig. 3 [file 44320_2024_32_MOESM12_ESM.zip › Figure 3/Figure 3H/Figure-3H-FBXO42-merged.tif]

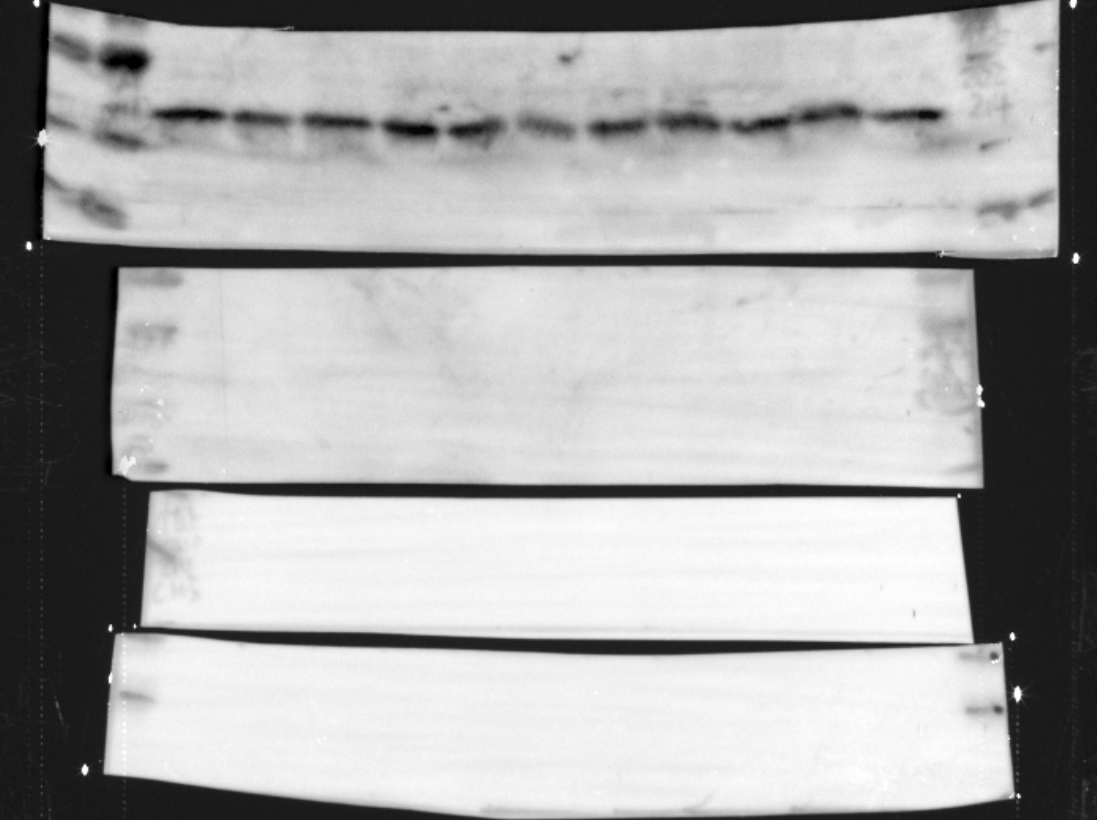

Supplement: Supplementary file 12 — Source data Fig. 3 [file 44320_2024_32_MOESM12_ESM.zip › Figure 3/Figure 3H/Figure-3H-Histone H3-merged.tif]

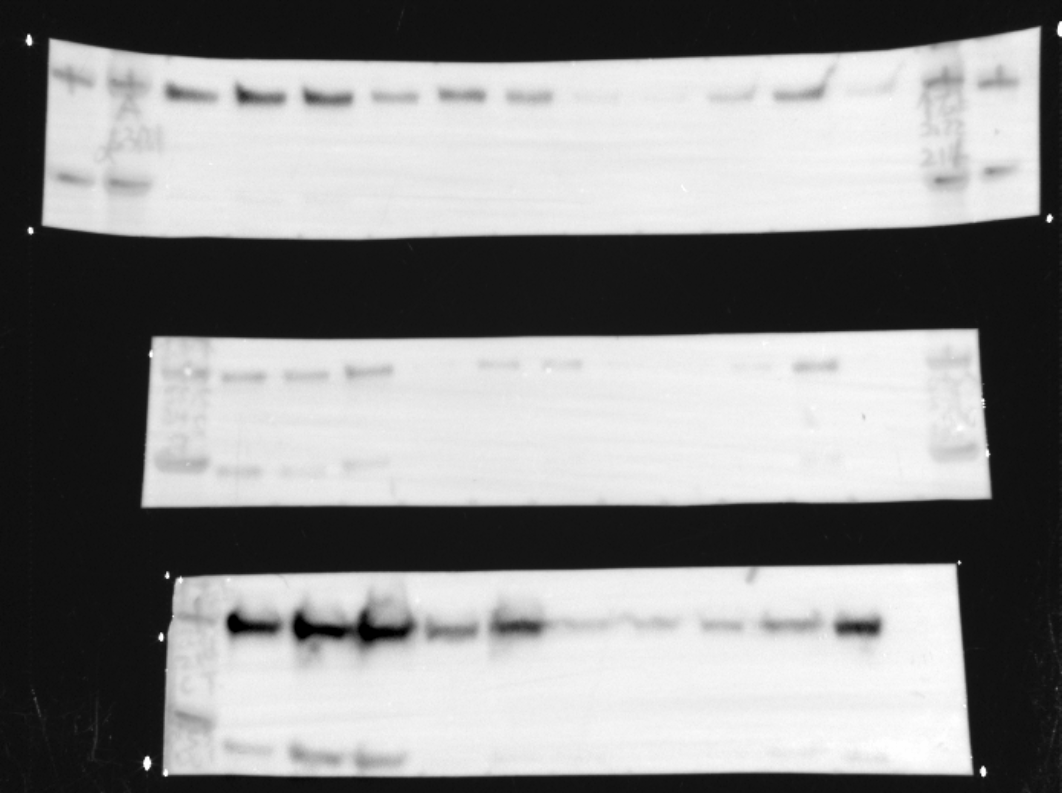

Supplement: Supplementary file 12 — Source data Fig. 3 [file 44320_2024_32_MOESM12_ESM.zip › Figure 3/Figure 3H/Figure-3H-p53DO1-merged.tif]

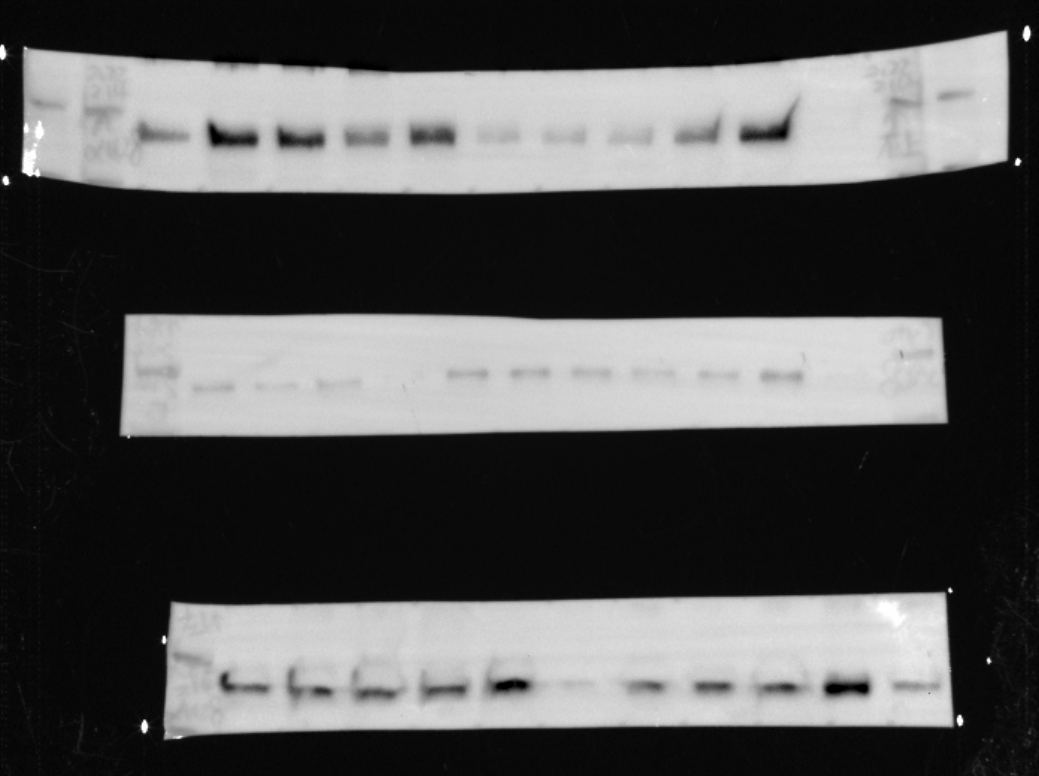

Supplement: Supplementary file 12 — Source data Fig. 3 [file 44320_2024_32_MOESM12_ESM.zip › Figure 3/Figure 3H/Figure-3H-USP28-merged.tif]

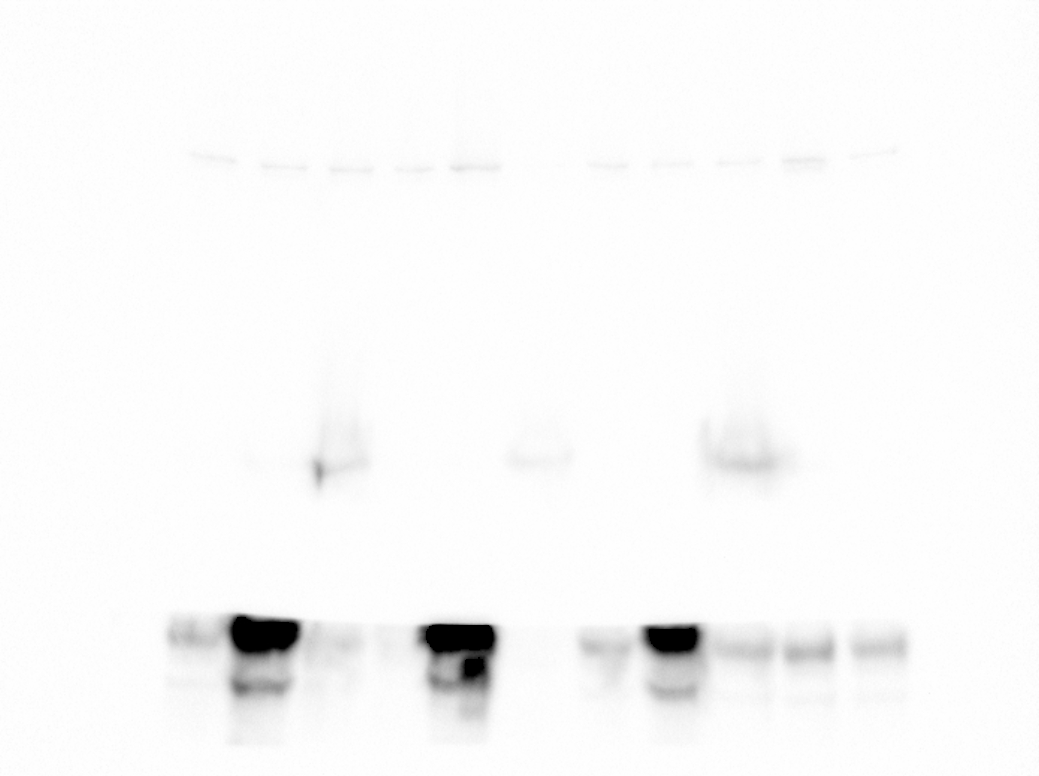

Supplement: Supplementary file 12 — Source data Fig. 3 [file 44320_2024_32_MOESM12_ESM.zip › Figure 3/Figure 3H/Non-annotated/CCDC6.jpg]

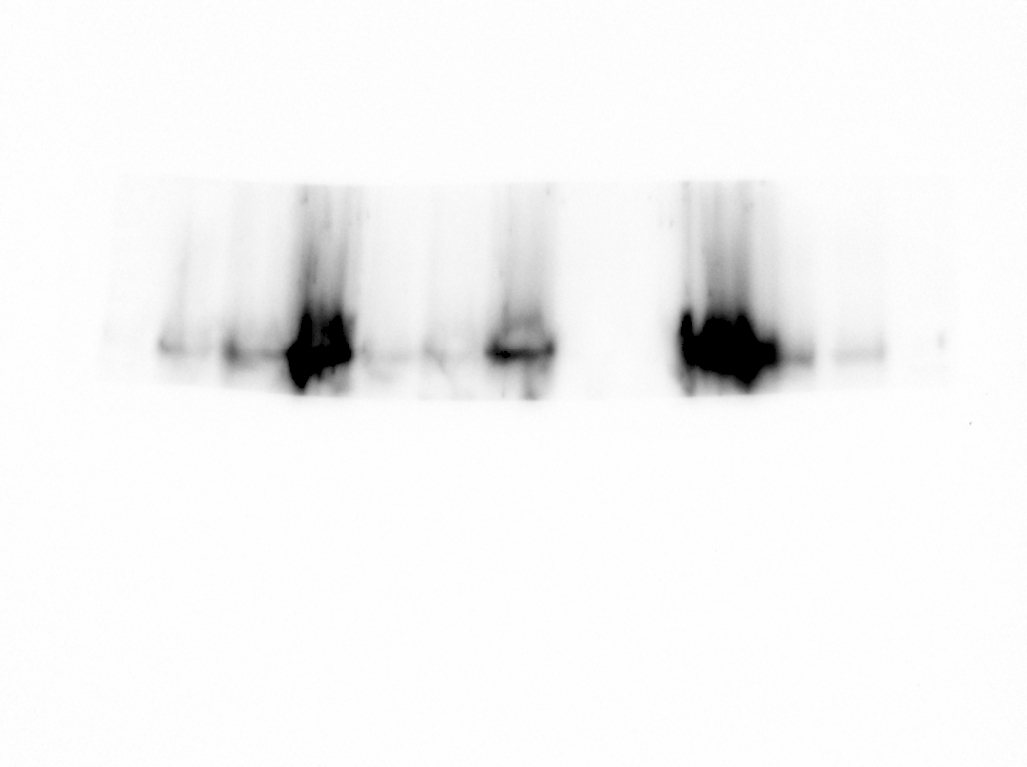

Supplement: Supplementary file 12 — Source data Fig. 3 [file 44320_2024_32_MOESM12_ESM.zip › Figure 3/Figure 3H/Non-annotated/FBXO42.jpg]

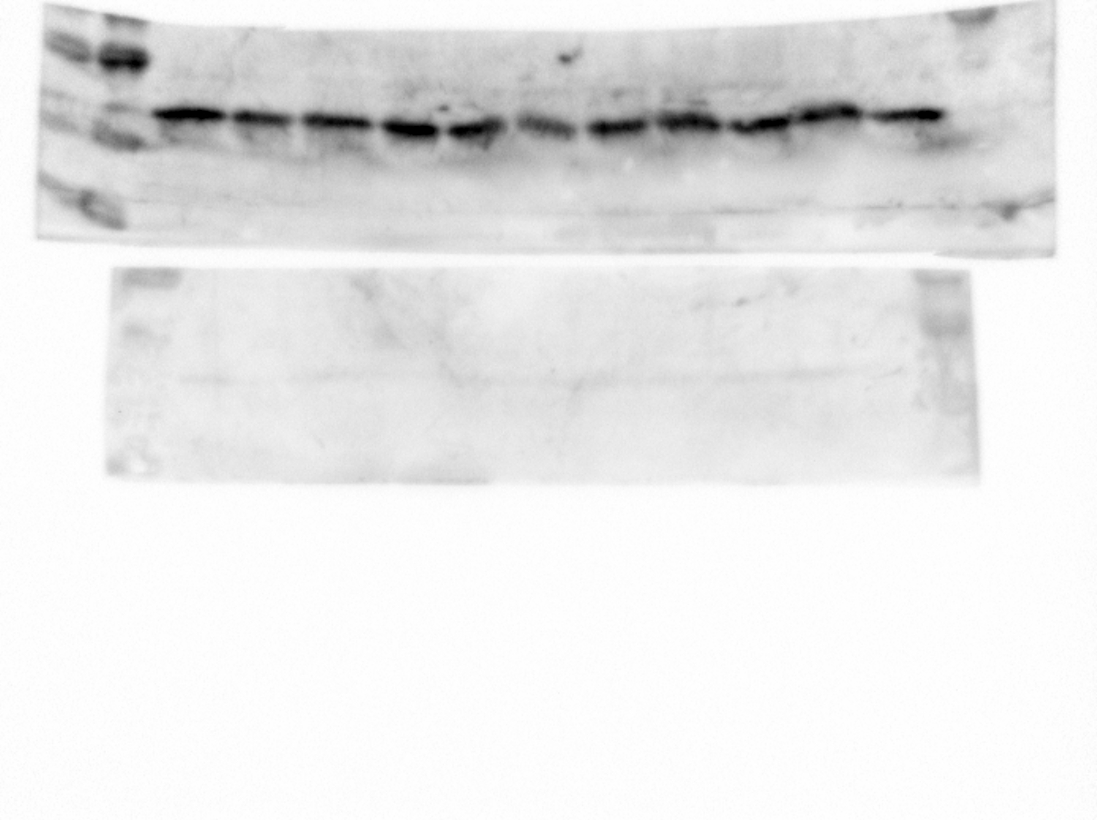

Supplement: Supplementary file 12 — Source data Fig. 3 [file 44320_2024_32_MOESM12_ESM.zip › Figure 3/Figure 3H/Non-annotated/H3.jpg]

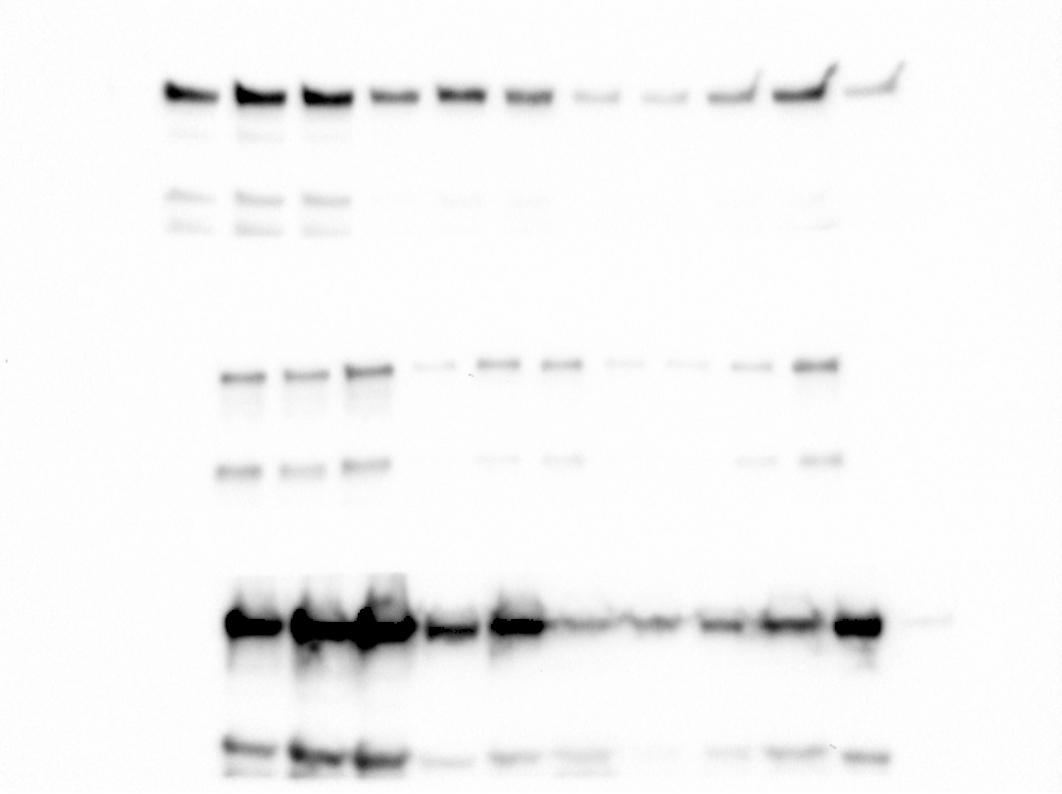

Supplement: Supplementary file 12 — Source data Fig. 3 [file 44320_2024_32_MOESM12_ESM.zip › Figure 3/Figure 3H/Non-annotated/p53DO1.jpg]

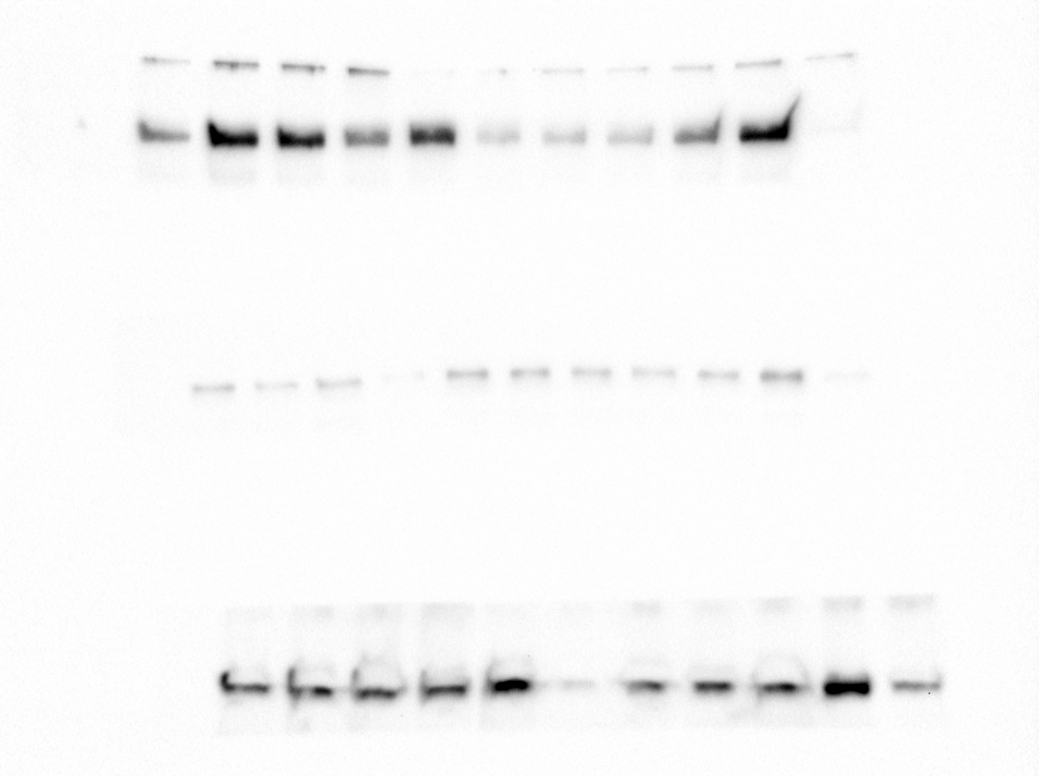

Supplement: Supplementary file 12 — Source data Fig. 3 [file 44320_2024_32_MOESM12_ESM.zip › Figure 3/Figure 3H/Non-annotated/USP28.jpg]

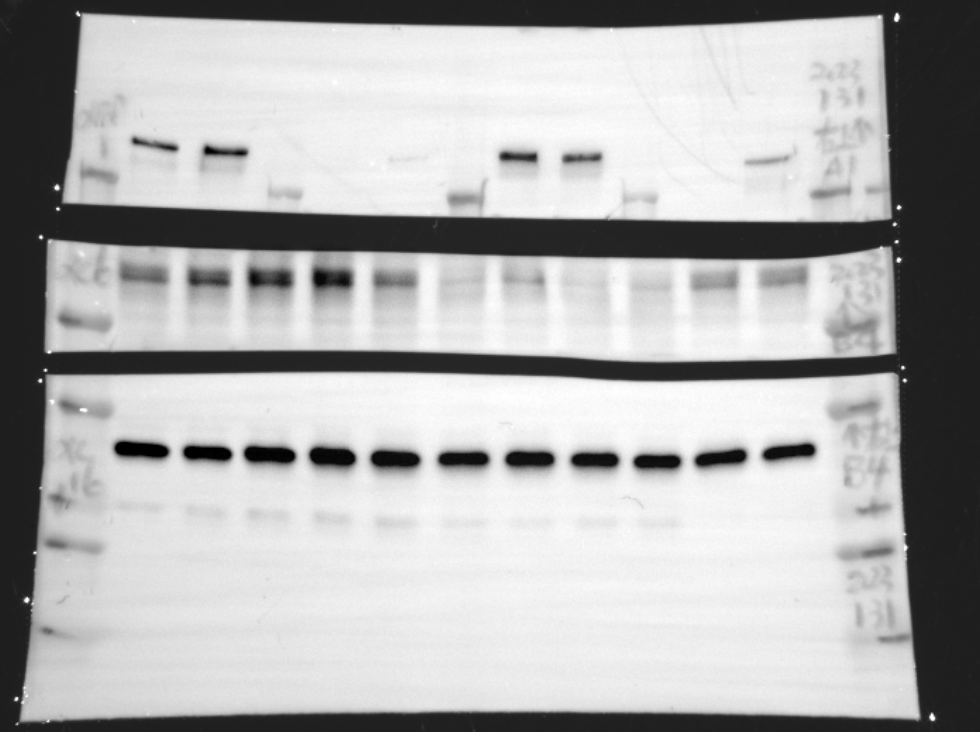

Supplement: Supplementary file 12 — Source data Fig. 3 [file 44320_2024_32_MOESM12_ESM.zip › Figure 3/Figure 3I/CCDC6-the middle gel.tif]

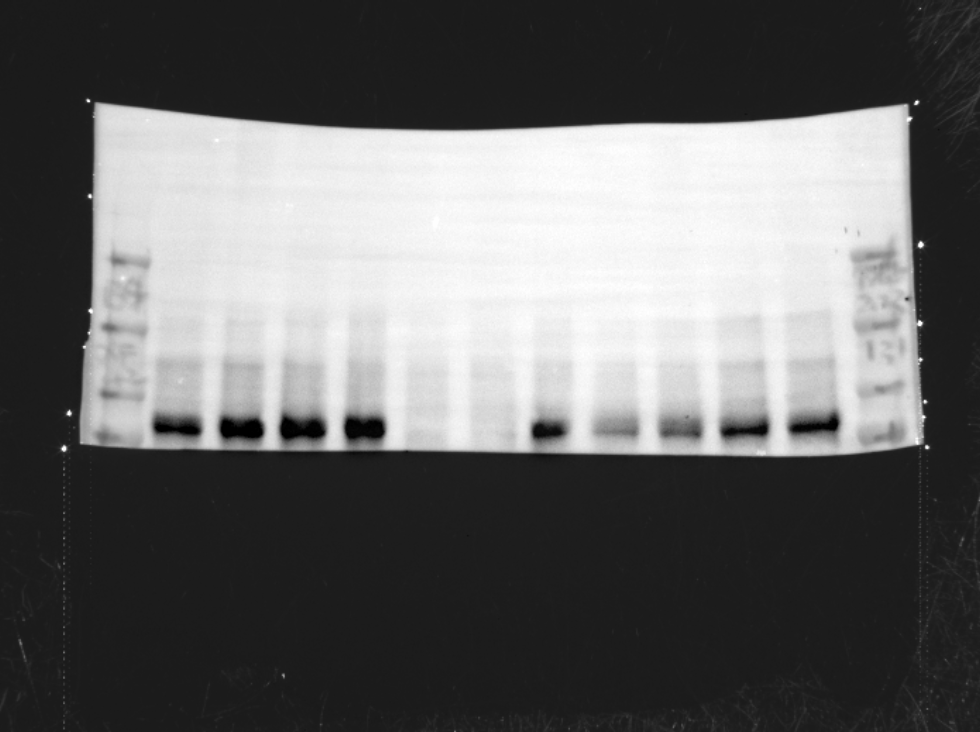

Supplement: Supplementary file 12 — Source data Fig. 3 [file 44320_2024_32_MOESM12_ESM.zip › Figure 3/Figure 3I/FBXO42.tif]

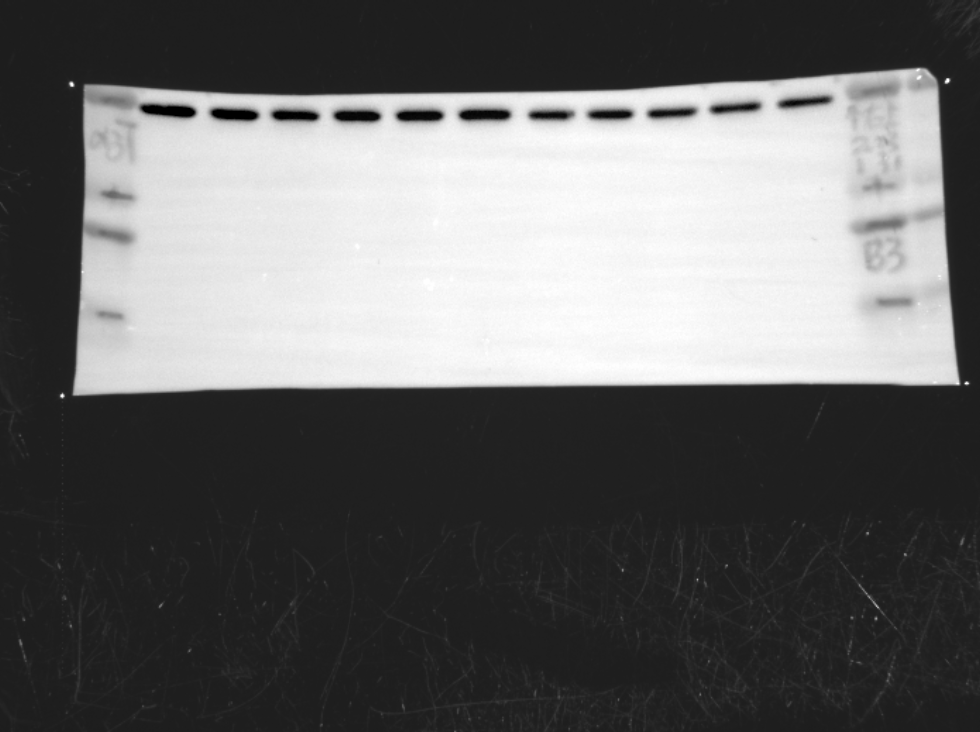

Supplement: Supplementary file 12 — Source data Fig. 3 [file 44320_2024_32_MOESM12_ESM.zip › Figure 3/Figure 3I/GAPDH.tif]

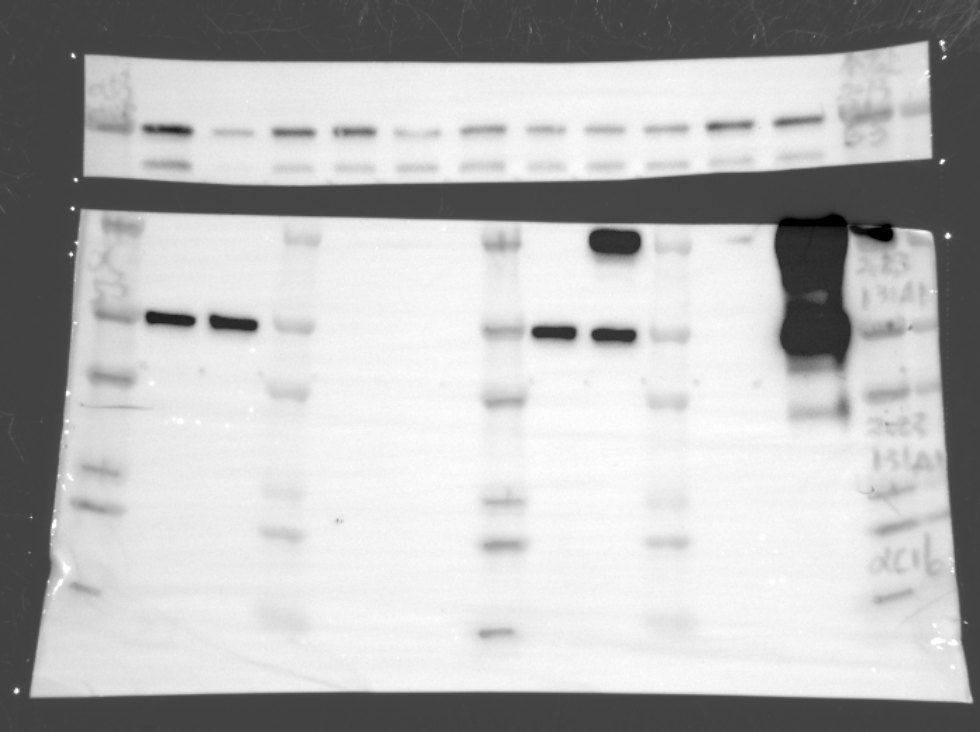

Supplement: Supplementary file 12 — Source data Fig. 3 [file 44320_2024_32_MOESM12_ESM.zip › Figure 3/Figure 3I/p53DO1.tif]

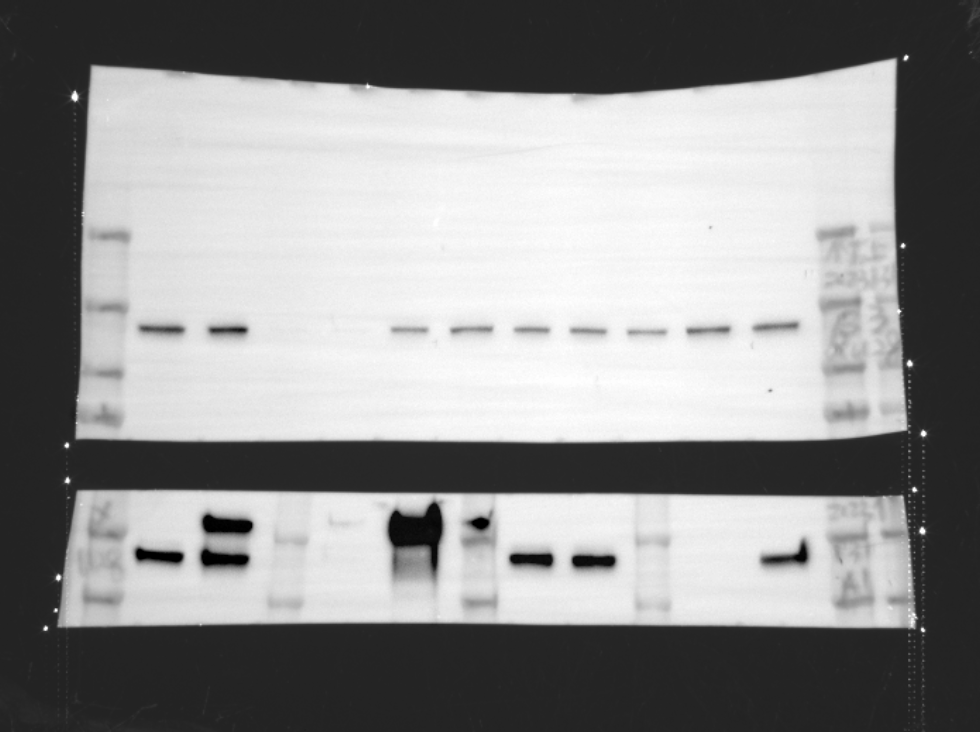

Supplement: Supplementary file 12 — Source data Fig. 3 [file 44320_2024_32_MOESM12_ESM.zip › Figure 3/Figure 3I/USP28.tif]

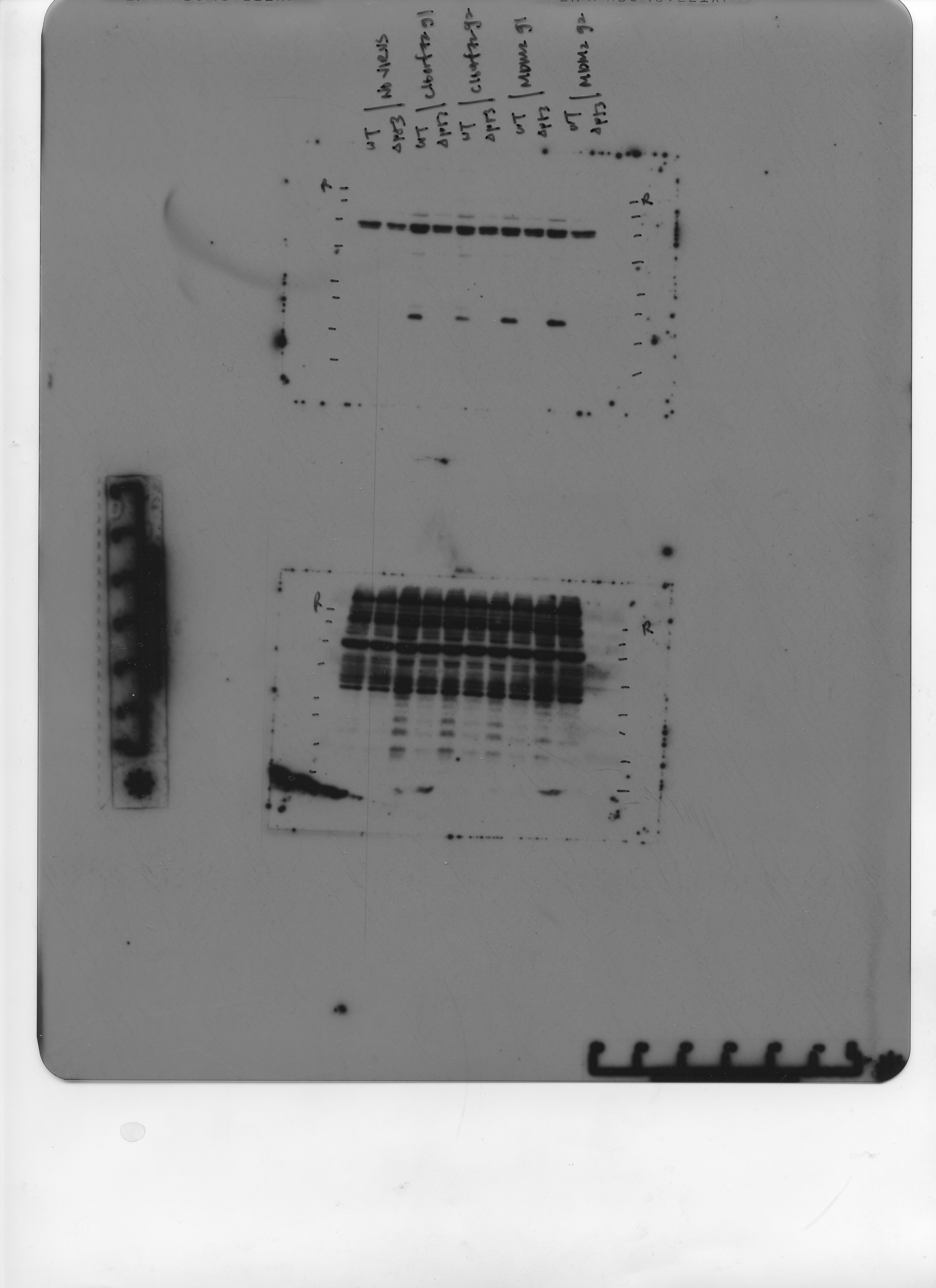

Supplement: Supplementary file 14 — Source data Fig. 5 [file 44320_2024_32_MOESM14_ESM.zip › Figure 5/Figure 5B/p21.tif]

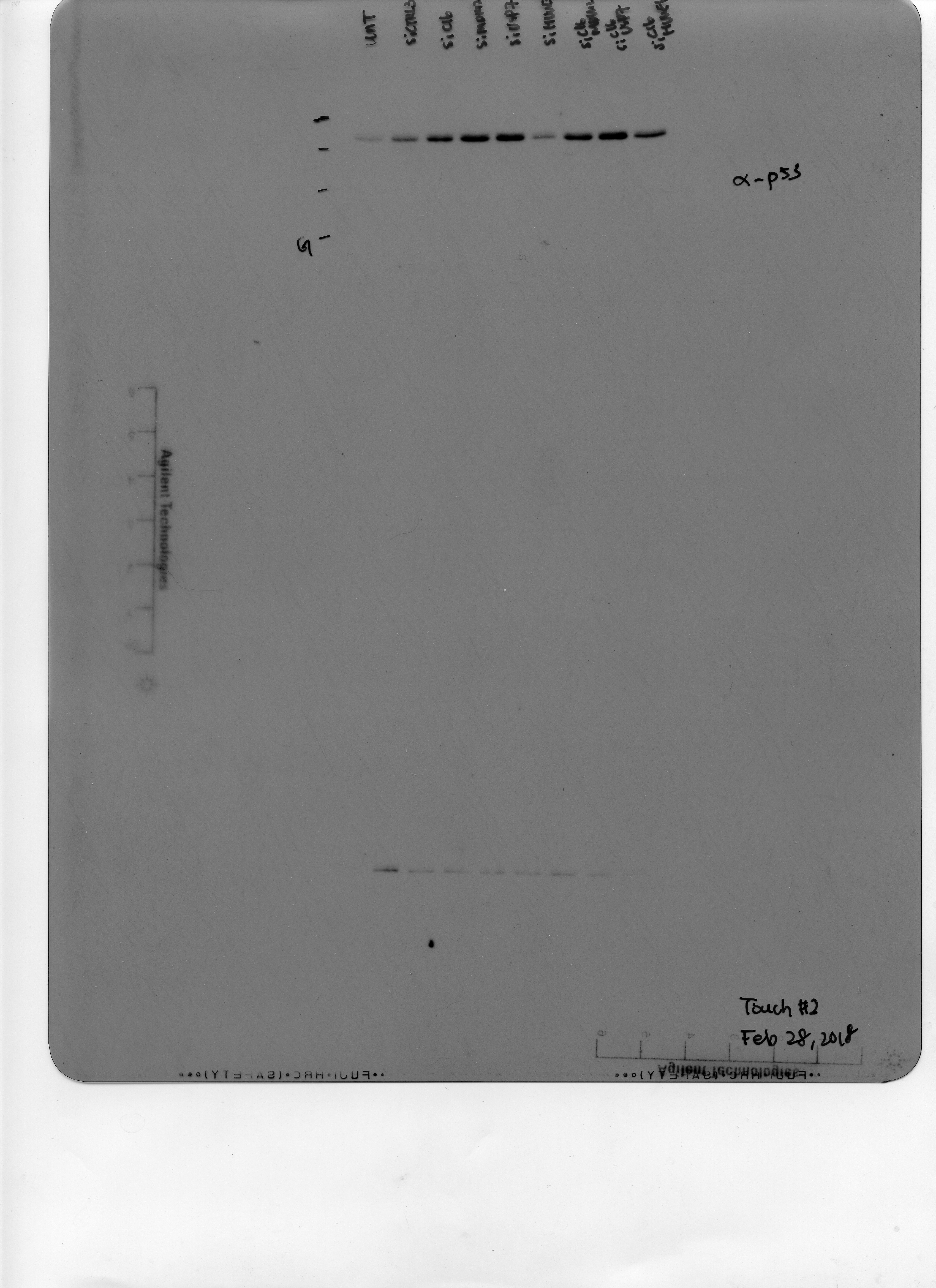

Supplement: Supplementary file 14 — Source data Fig. 5 [file 44320_2024_32_MOESM14_ESM.zip › Figure 5/Figure 5E/si_p53.tif]

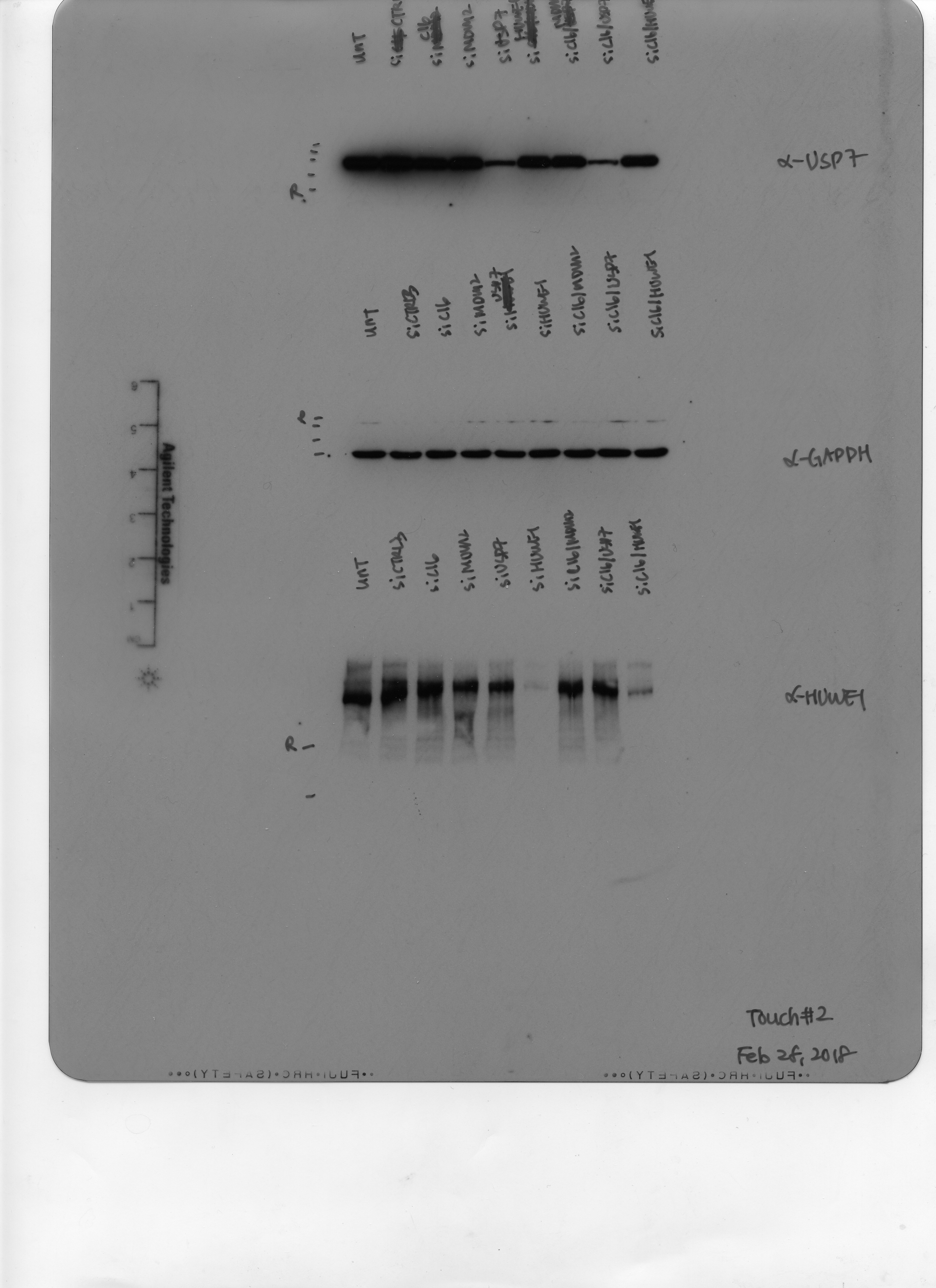

Supplement: Supplementary file 14 — Source data Fig. 5 [file 44320_2024_32_MOESM14_ESM.zip › Figure 5/Figure 5E/si_USP7_GAPDH_HUWE1126.tif]

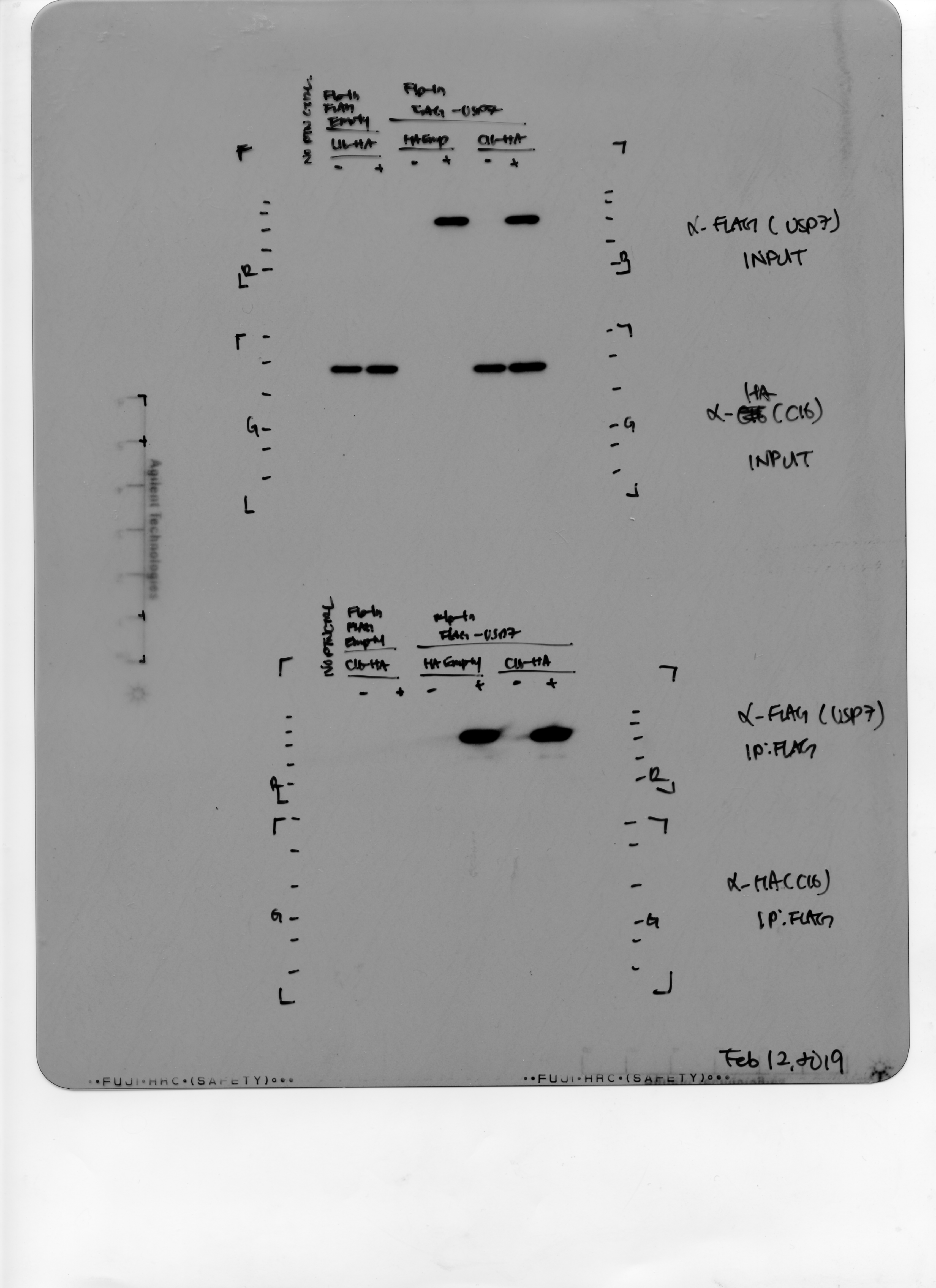

Supplement: Supplementary file 14 — Source data Fig. 5 [file 44320_2024_32_MOESM14_ESM.zip › Figure 5/Figure 5H/FLAG and HA INPUT_FLAG IP_292.tif]

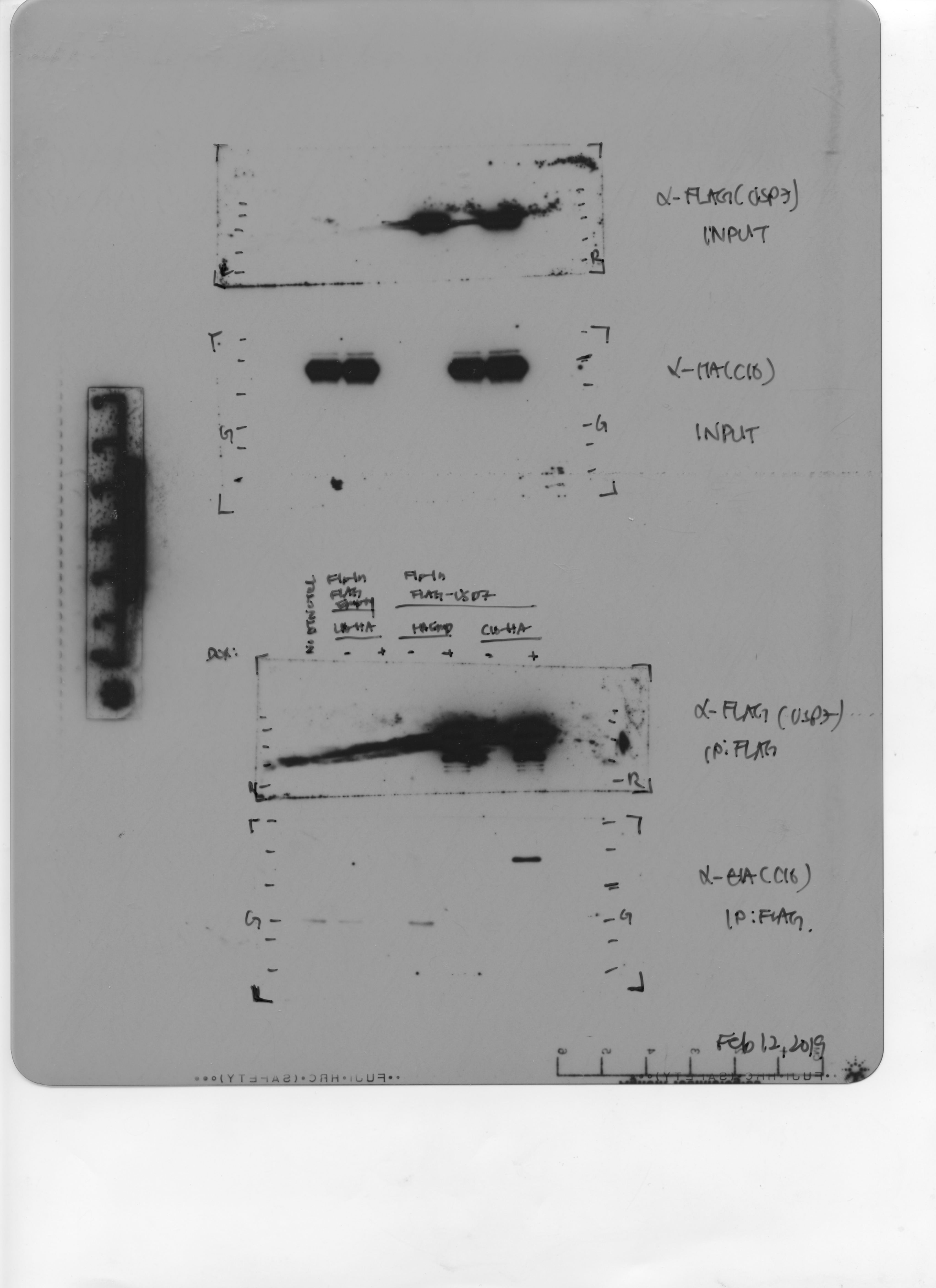

Supplement: Supplementary file 14 — Source data Fig. 5 [file 44320_2024_32_MOESM14_ESM.zip › Figure 5/Figure 5H/HA_IP_293.tif]

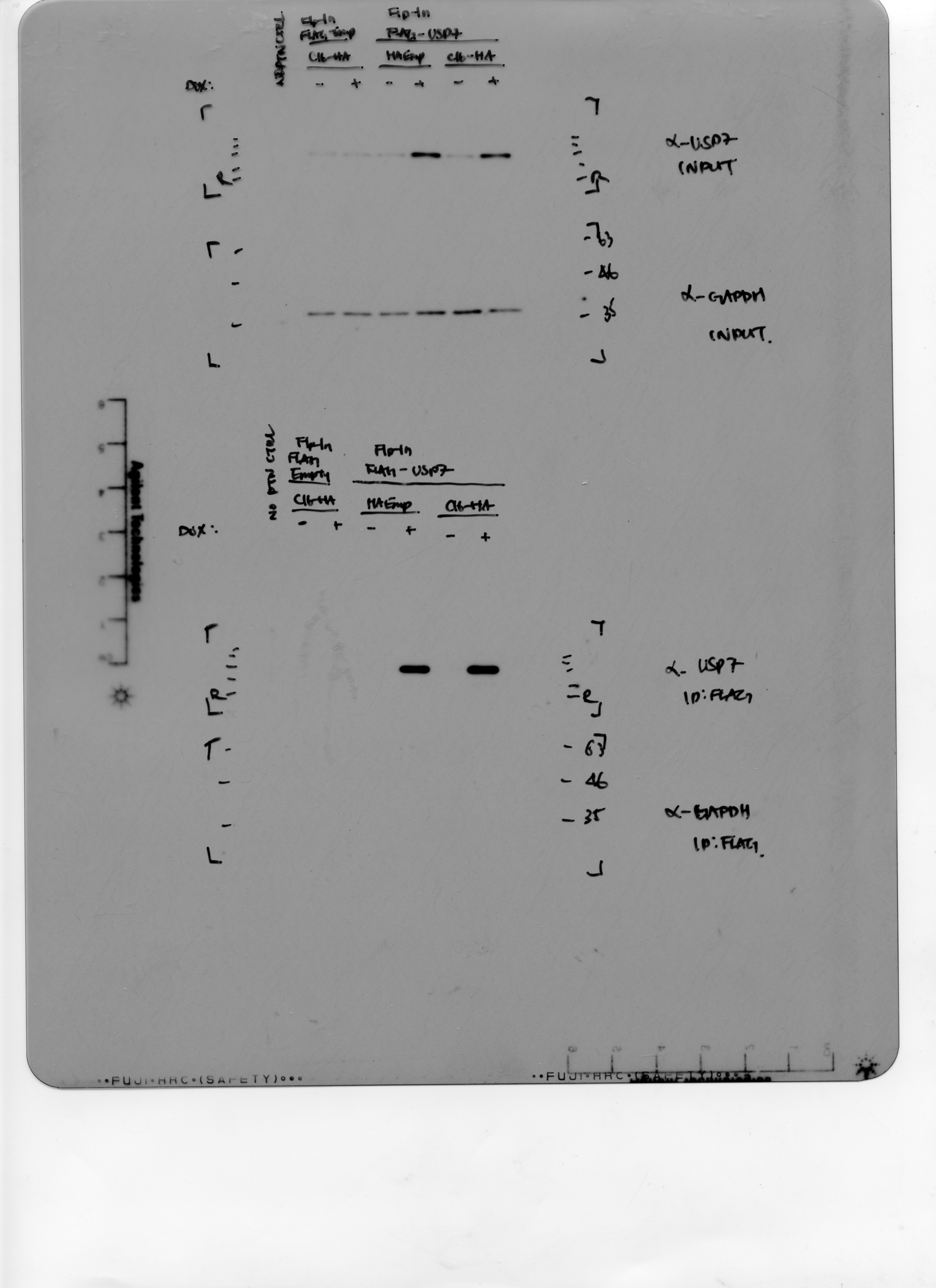

Supplement: Supplementary file 14 — Source data Fig. 5 [file 44320_2024_32_MOESM14_ESM.zip › Figure 5/Figure 5H/USP7 and GAPDH INPUT_295.tif]

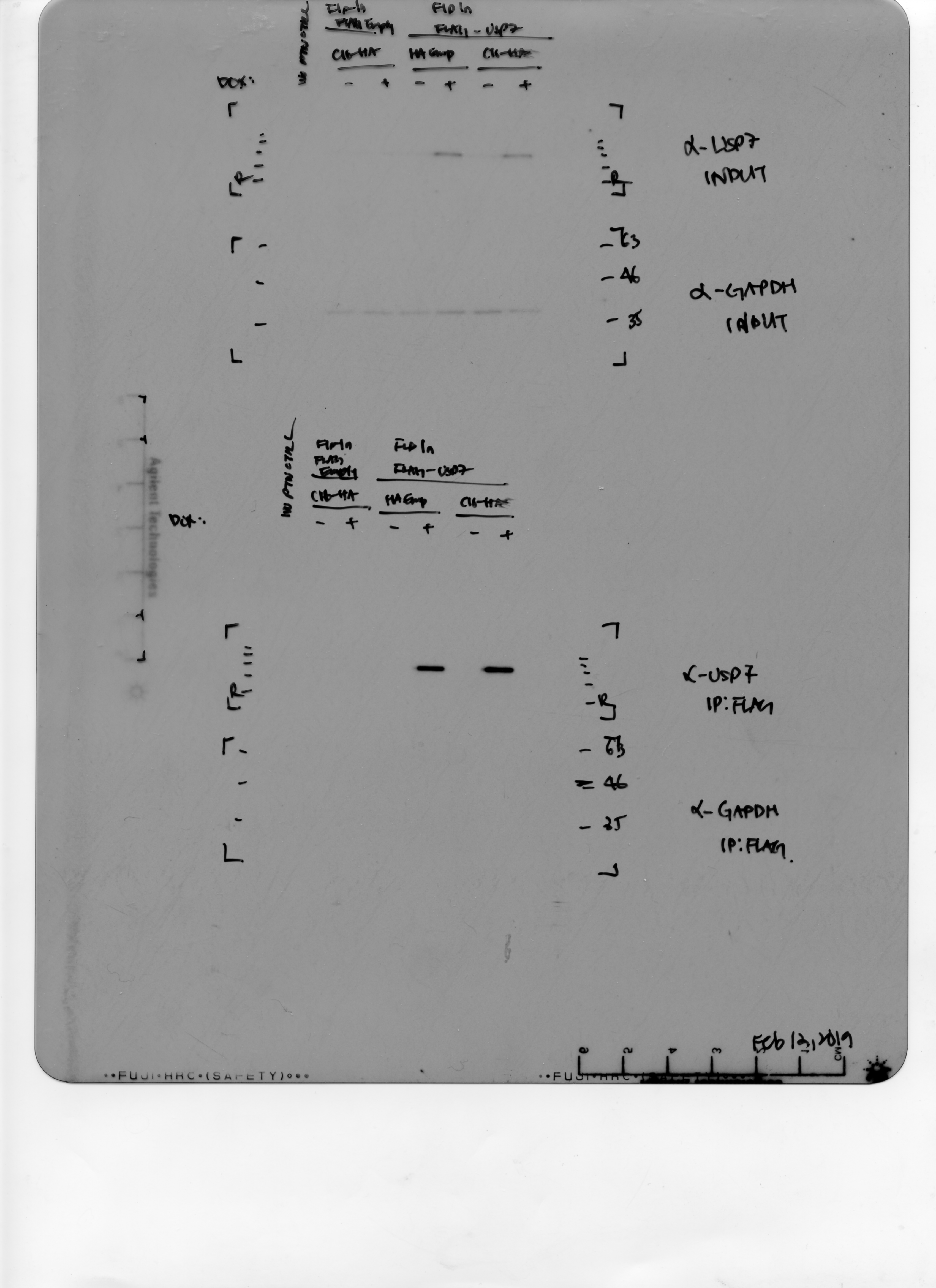

Supplement: Supplementary file 14 — Source data Fig. 5 [file 44320_2024_32_MOESM14_ESM.zip › Figure 5/Figure 5H/USP7_IP_294.tif]

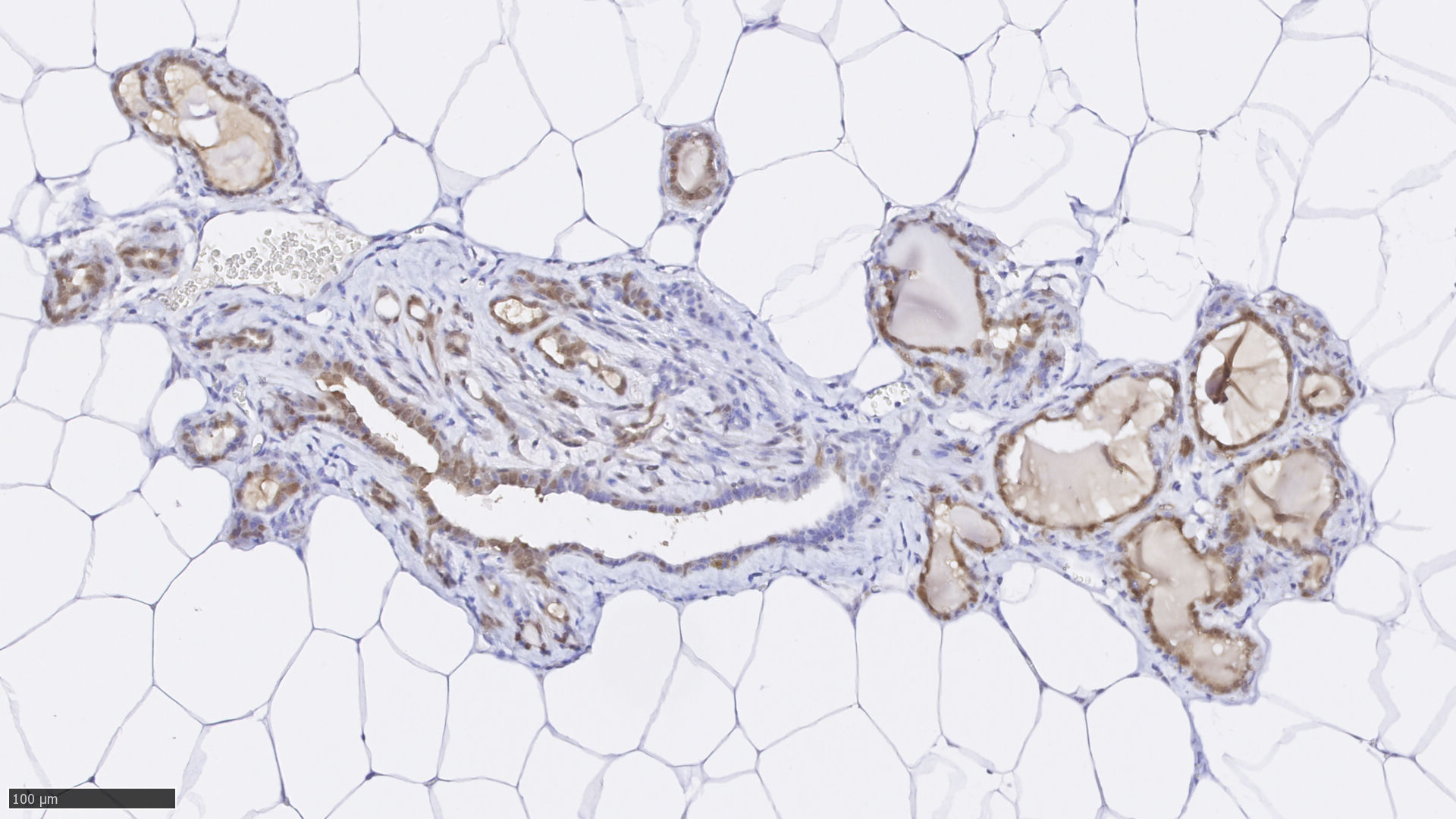

Supplement: Supplementary file 15 — Source data Fig. 6 [file 44320_2024_32_MOESM15_ESM.zip › Figure 6/Figure 6F/GFP B8557 hyper NR3 - 2022-03-24 18.52.jpg]

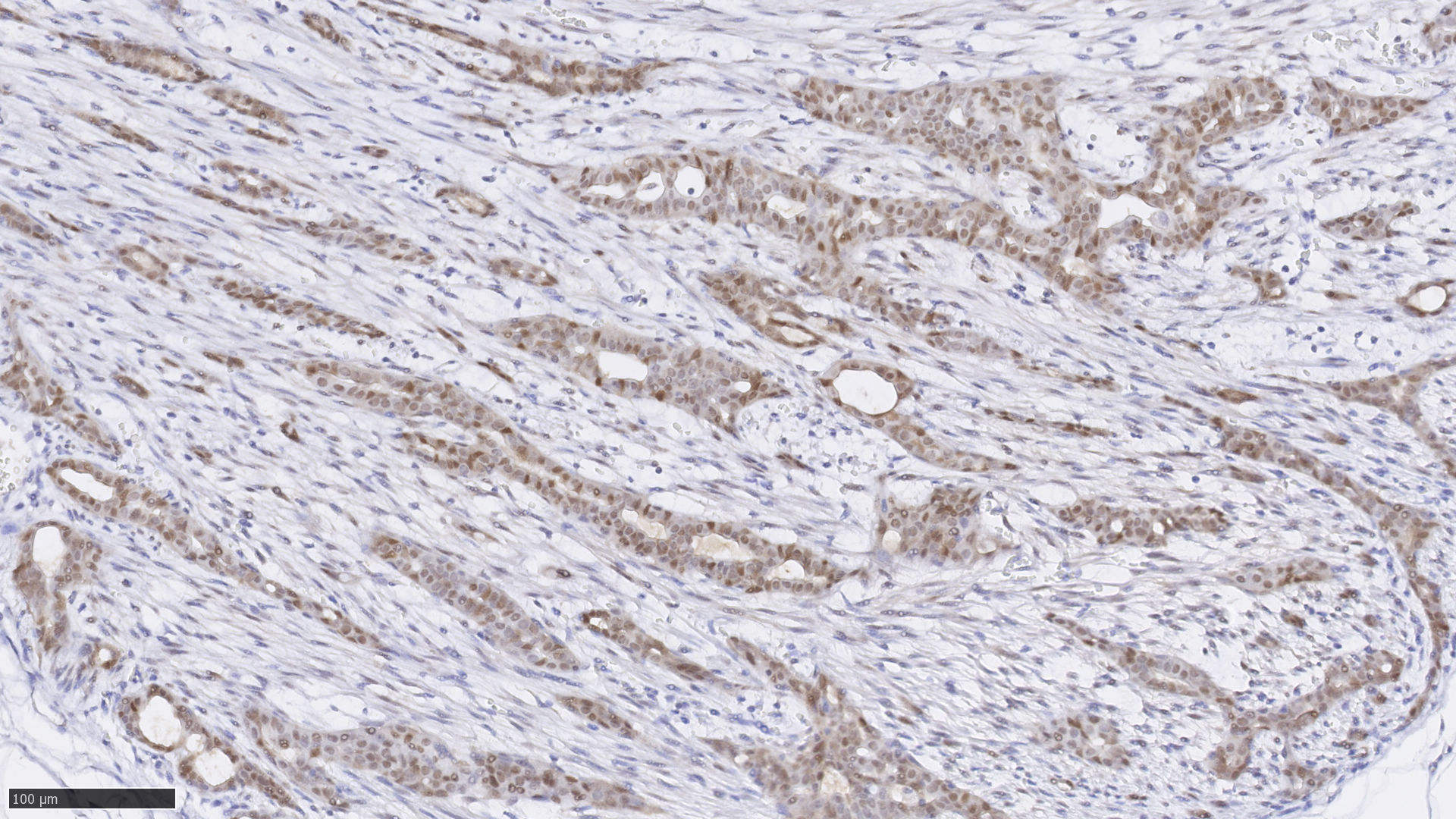

Supplement: Supplementary file 15 — Source data Fig. 6 [file 44320_2024_32_MOESM15_ESM.zip › Figure 6/Figure 6F/GFP B8557 tumour 1 NR3 - 2022-03-24 18.52.jpg]

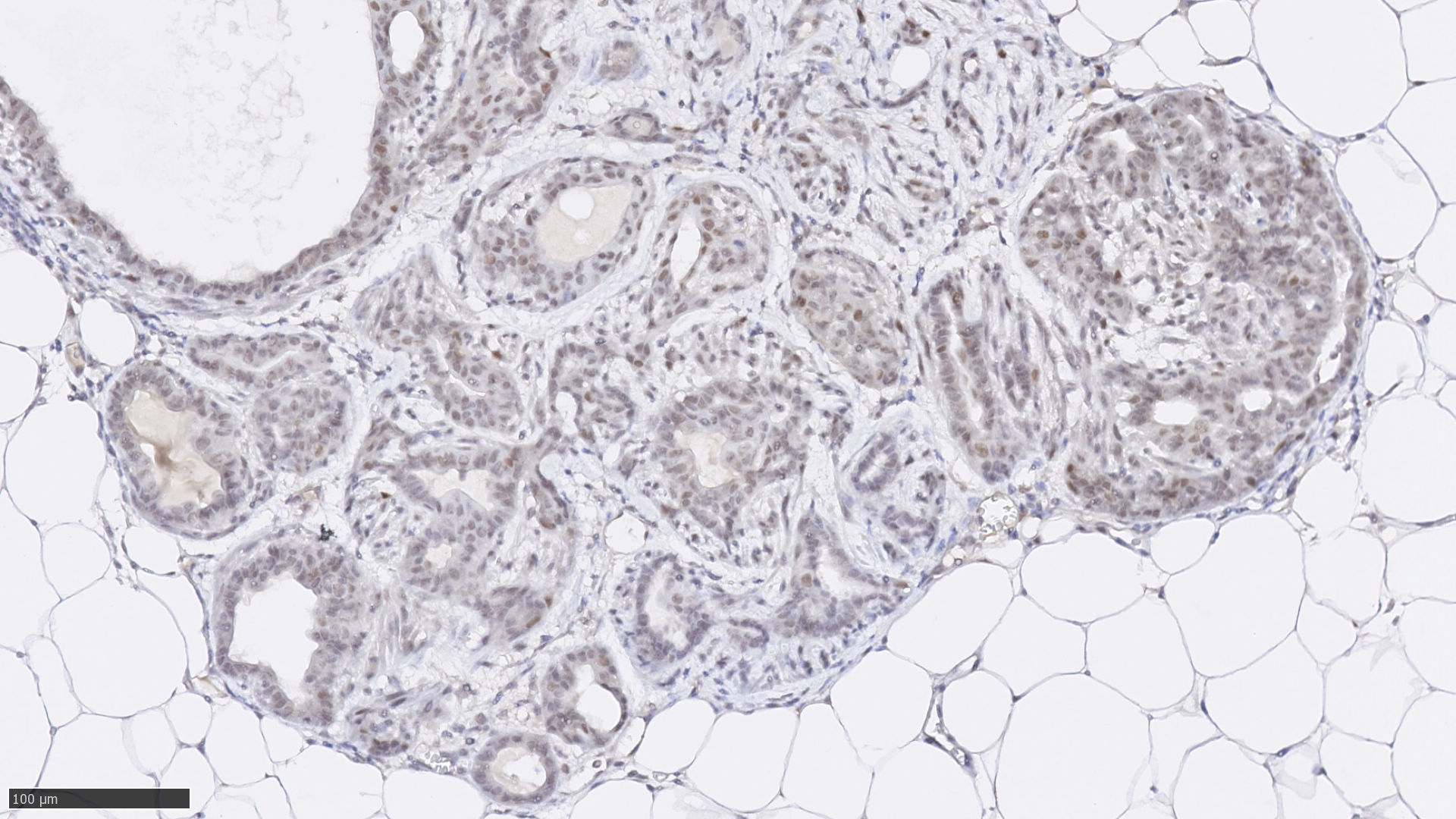

Supplement: Supplementary file 15 — Source data Fig. 6 [file 44320_2024_32_MOESM15_ESM.zip › Figure 6/Figure 6F/p53 B8553 hyper NR4 - 2022-03-24 21.07.jpg]

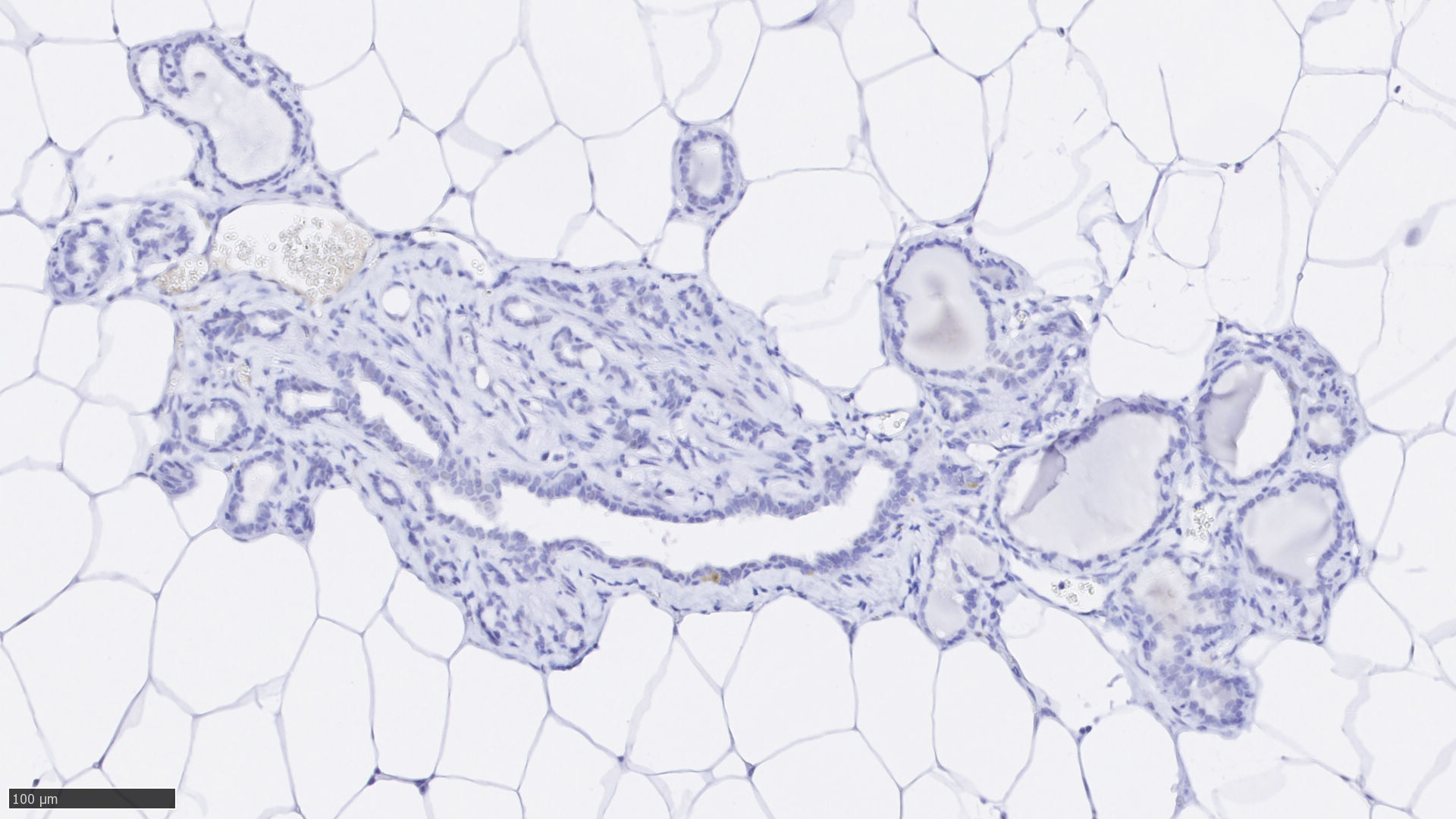

Supplement: Supplementary file 15 — Source data Fig. 6 [file 44320_2024_32_MOESM15_ESM.zip › Figure 6/Figure 6F/p53 B8557 hyper NR3 - 2022-03-24 21.02.jpg]

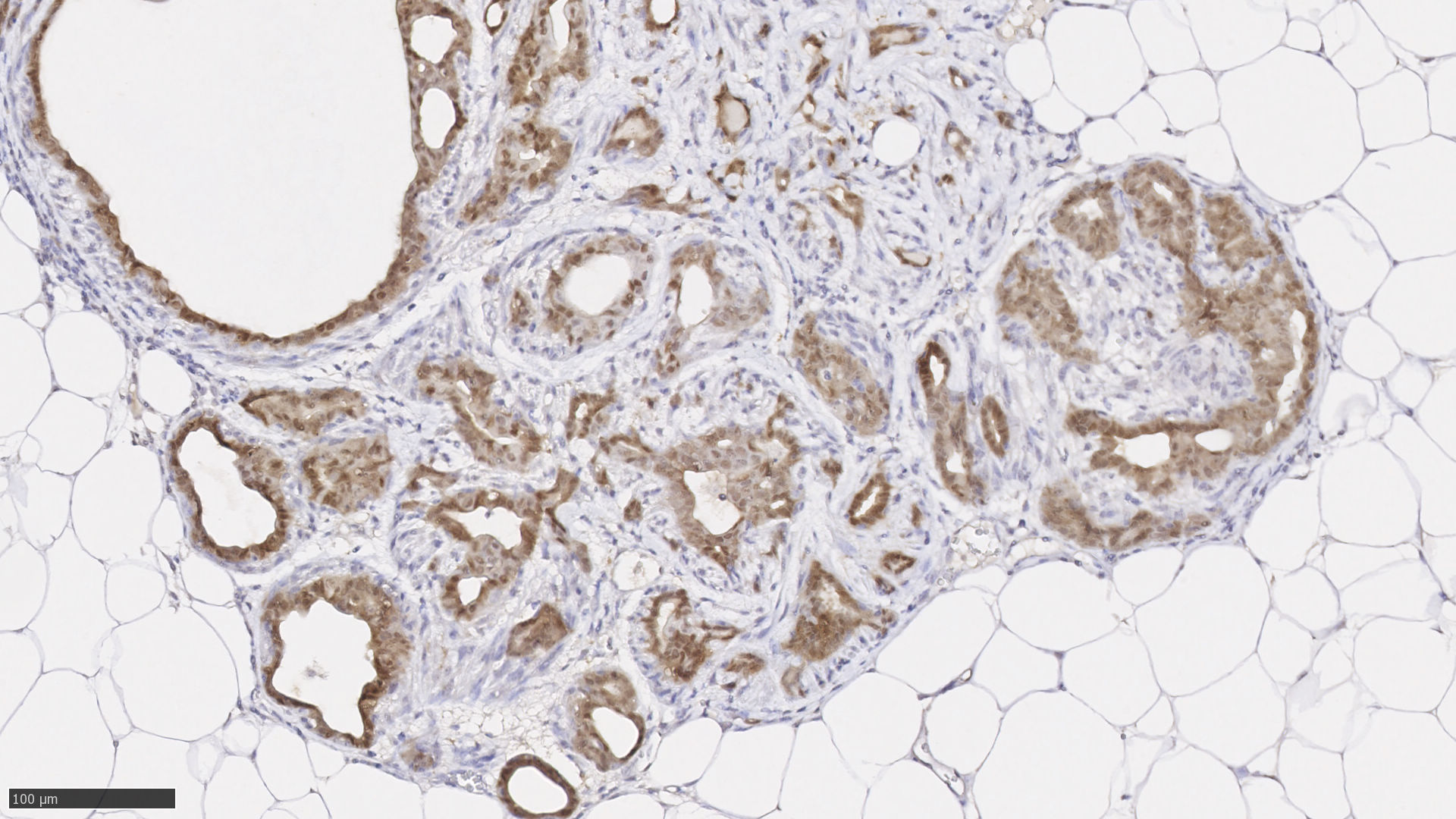

Supplement: Supplementary file 15 — Source data Fig. 6 [file 44320_2024_32_MOESM15_ESM.zip › Figure 6/Figure 6F/GFP B8553 hyper NR4 - 2022-03-28 15.02.jpg]

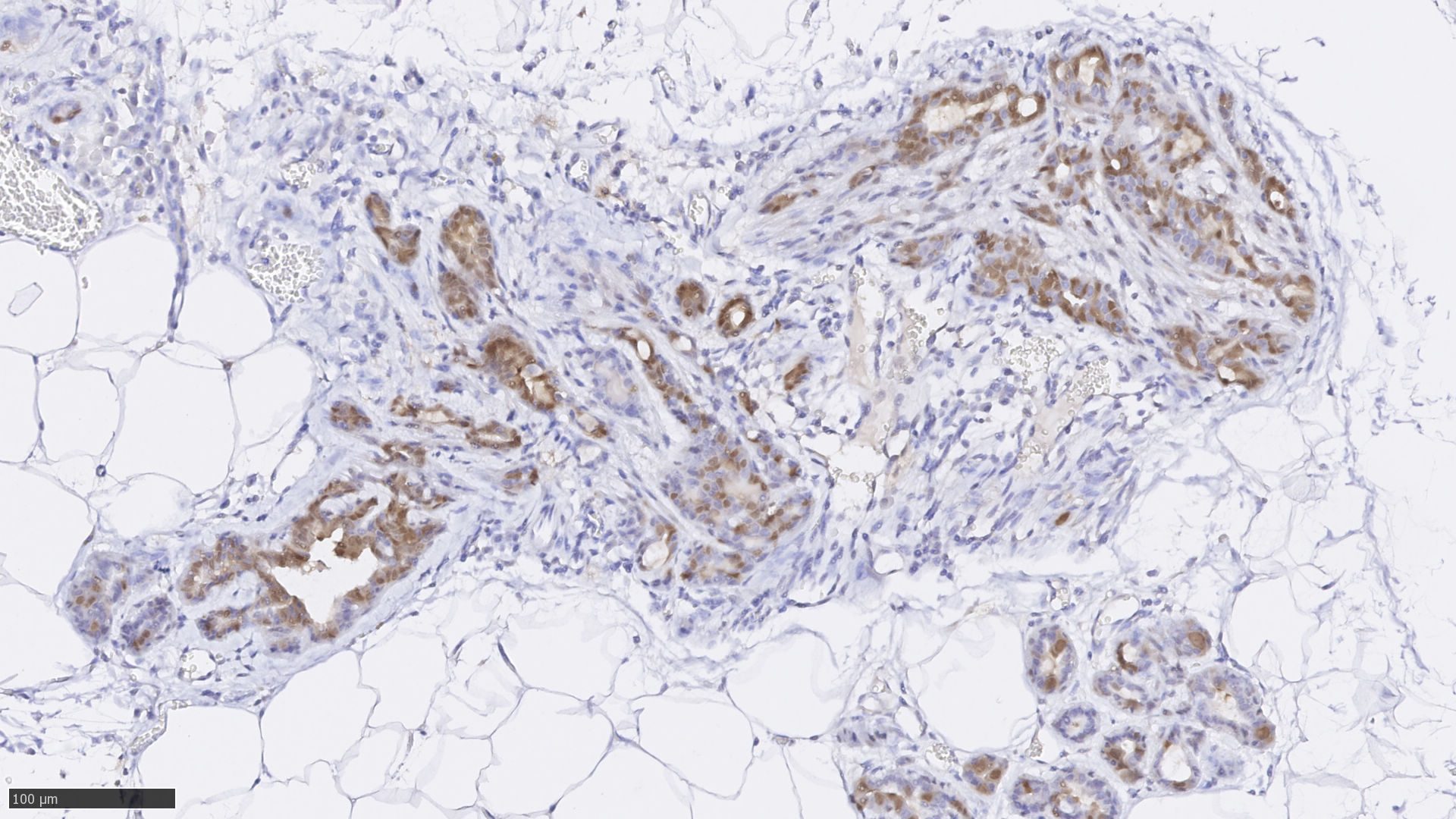

Supplement: Supplementary file 15 — Source data Fig. 6 [file 44320_2024_32_MOESM15_ESM.zip › Figure 6/Figure 6F/GFP B8557 hyper 1 NR3 - 2022-03-24 18.52.jpg]

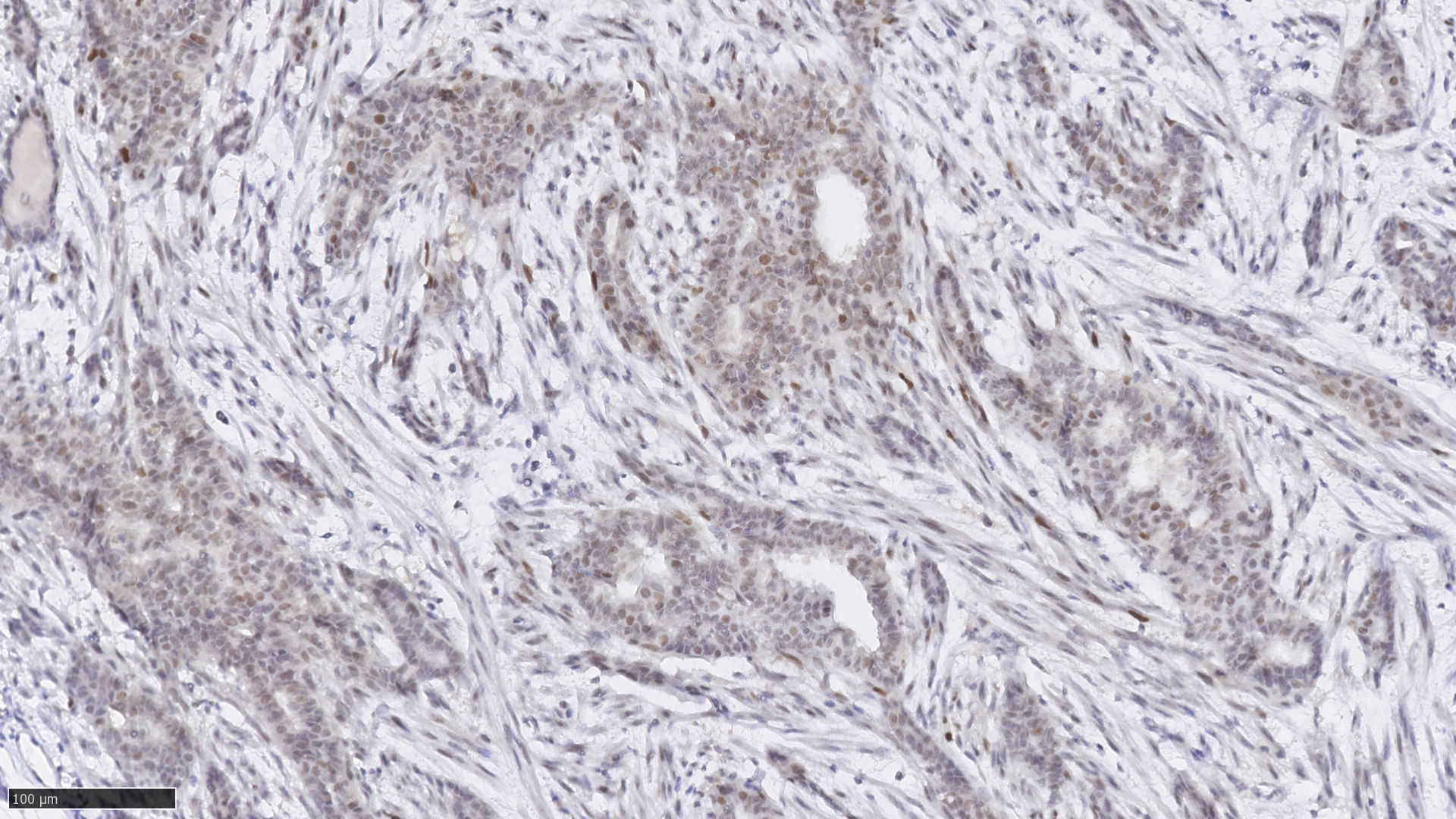

Supplement: Supplementary file 15 — Source data Fig. 6 [file 44320_2024_32_MOESM15_ESM.zip › Figure 6/Figure 6F/p53 B8553 tumour 1 NL3 - 2022-03-24 20.56.jpg]

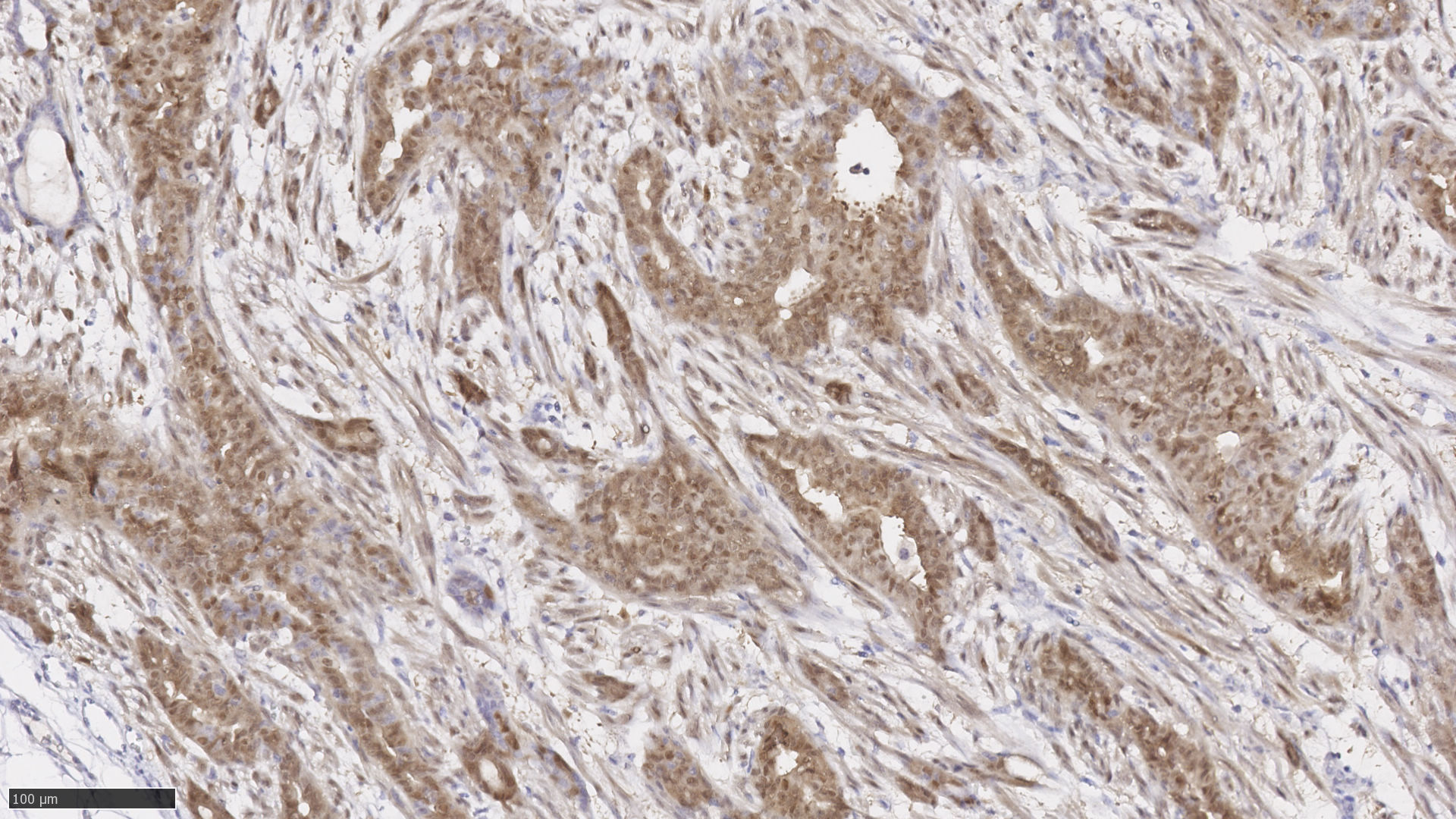

Supplement: Supplementary file 15 — Source data Fig. 6 [file 44320_2024_32_MOESM15_ESM.zip › Figure 6/Figure 6F/GFP B8553 tumour 1 NL3 - 2022-03-28 15.06.jpg]

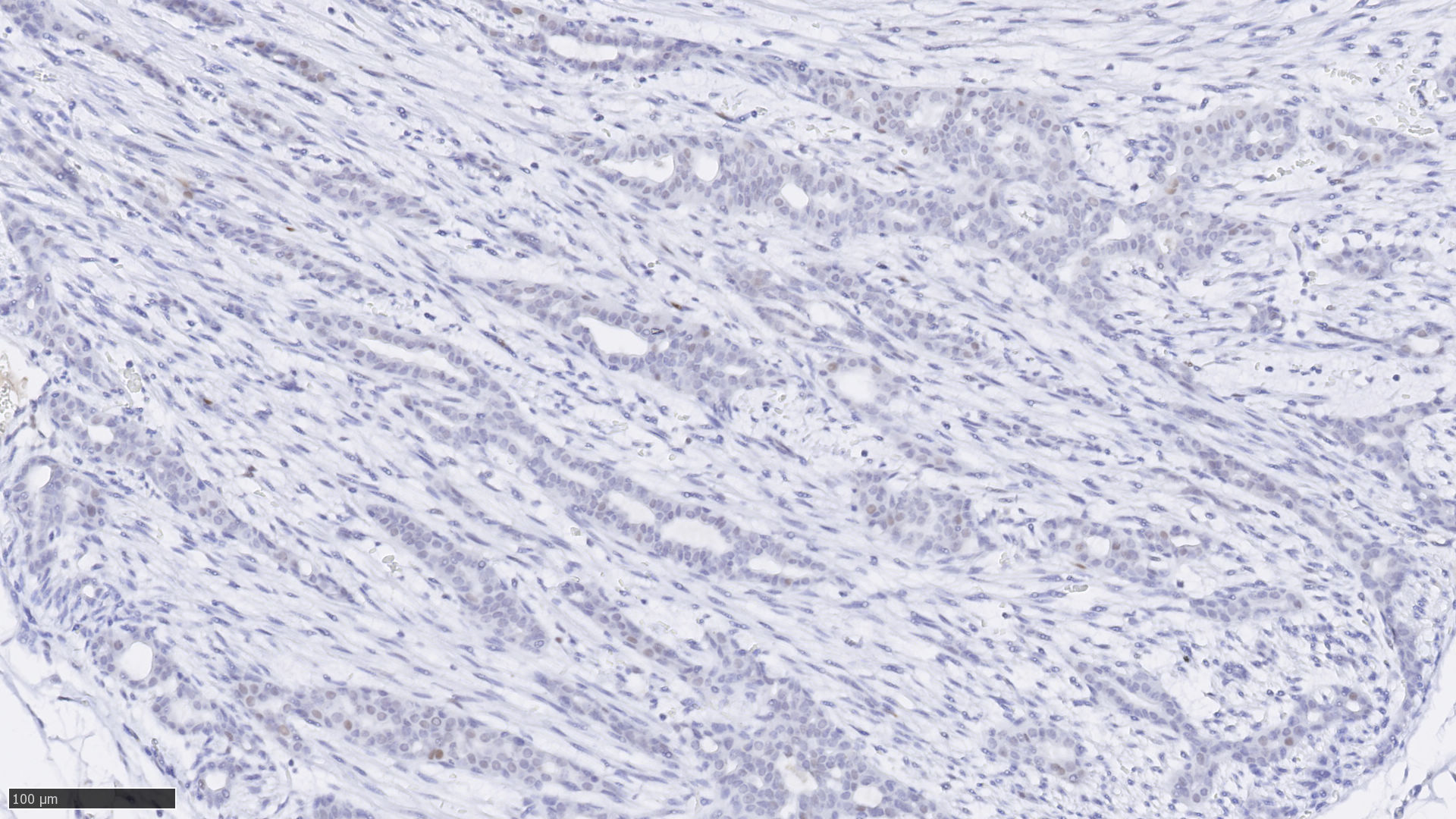

Supplement: Supplementary file 15 — Source data Fig. 6 [file 44320_2024_32_MOESM15_ESM.zip › Figure 6/Figure 6F/p53 B8557 tumour 1NR3 - 2022-03-24 21.02.jpg]

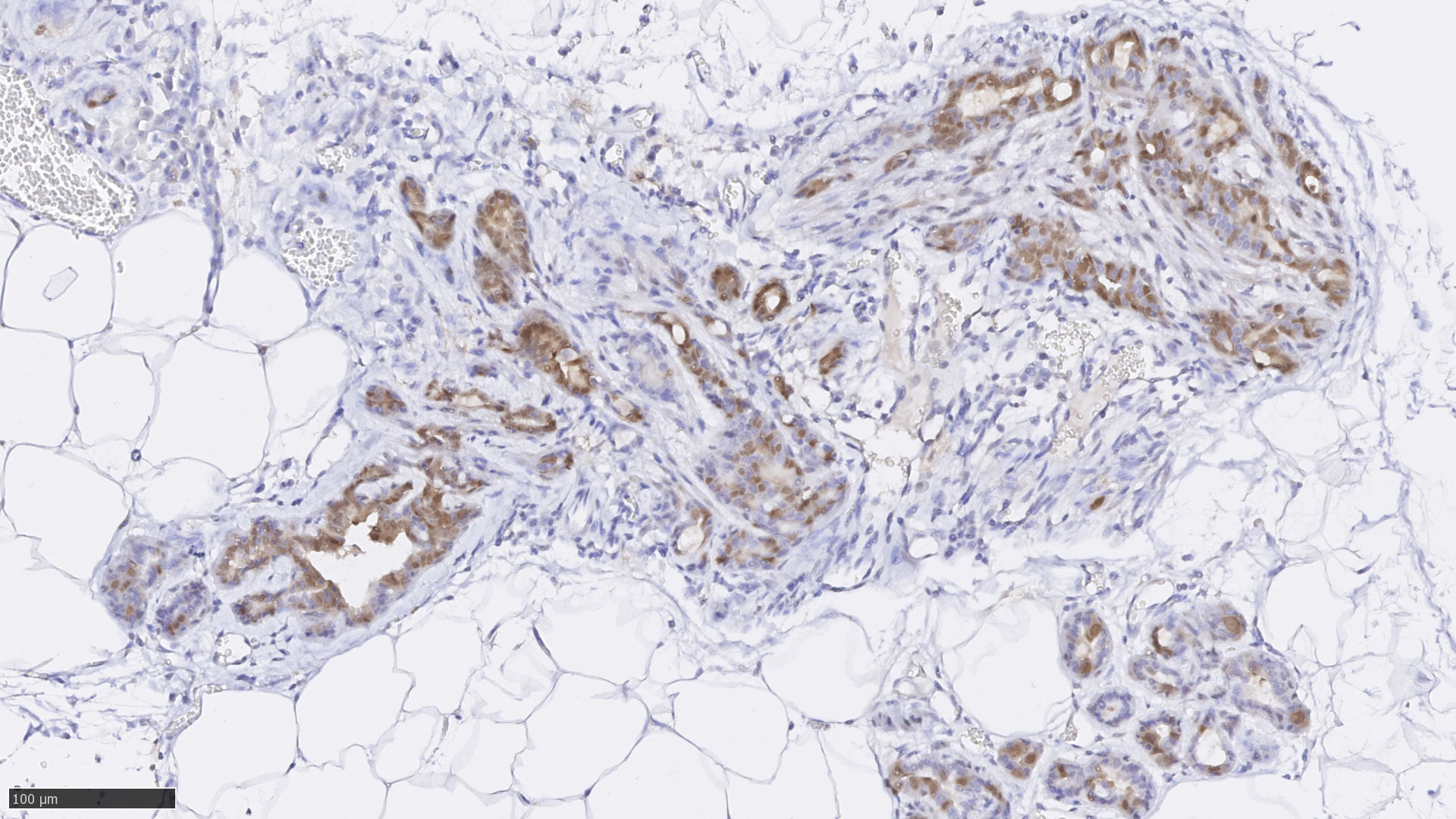

Supplement: Supplementary file 15 — Source data Fig. 6 [file 44320_2024_32_MOESM15_ESM.zip › Figure 6/Figure 6F/GFP B8557 hyper 2NR3 - 2022-03-24 18.52.jpg]

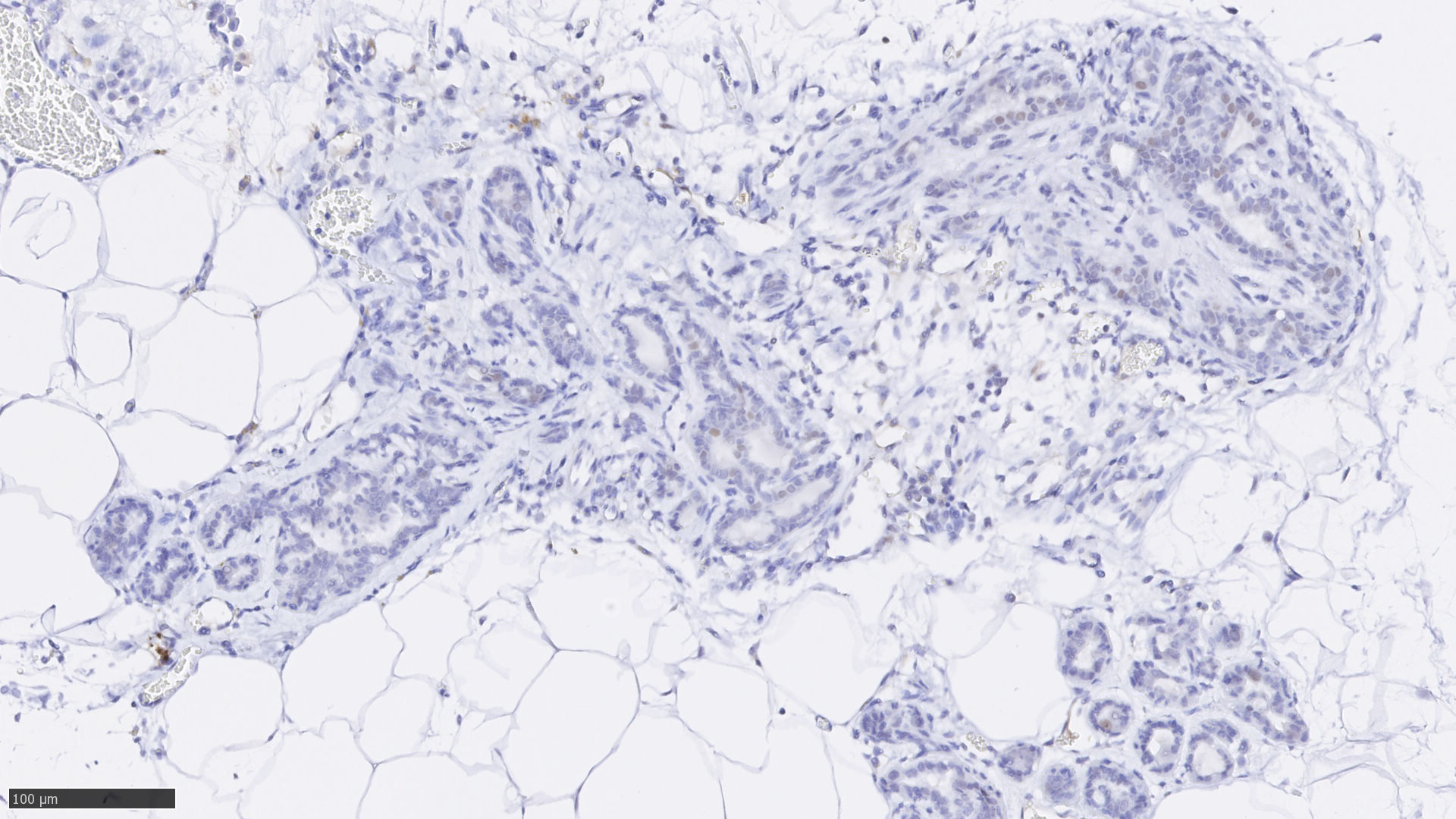

Supplement: Supplementary file 15 — Source data Fig. 6 [file 44320_2024_32_MOESM15_ESM.zip › Figure 6/Figure 6F/p53 B8557 hyper 2 NR3 - 2022-03-24 21.02.jpg]

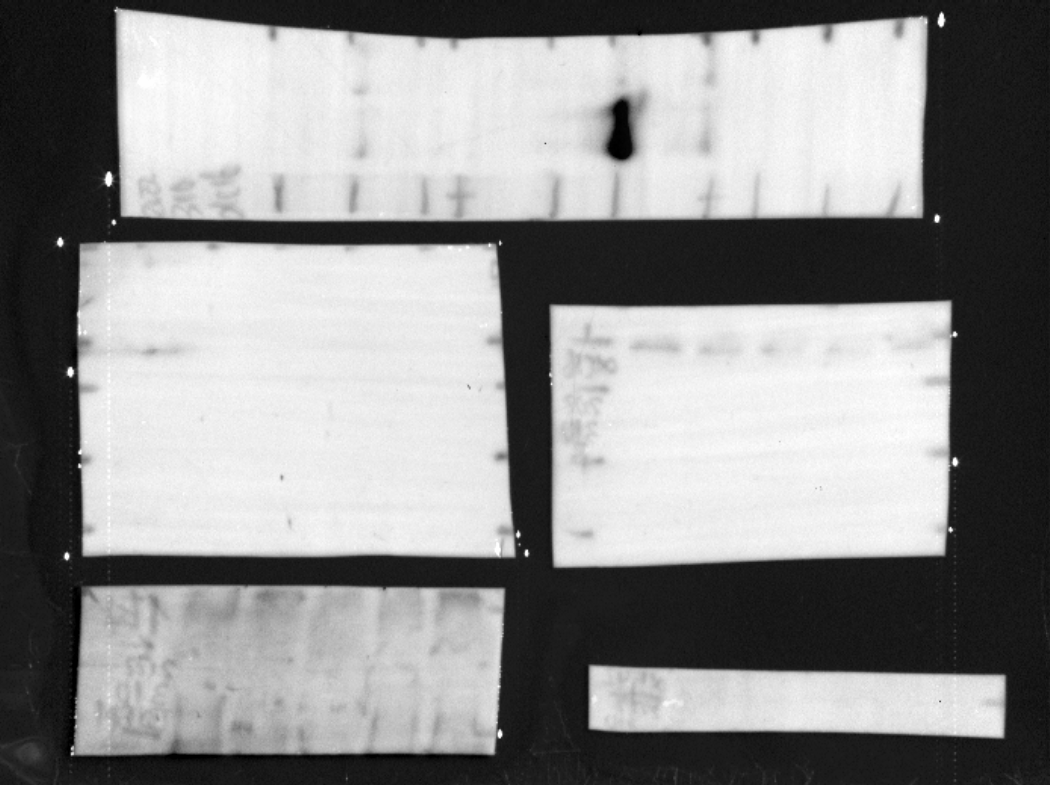

Supplement: Supplementary file 15 — Source data Fig. 6 [file 44320_2024_32_MOESM15_ESM.zip › Figure 6/Figure 6D/C16orf72.tif]

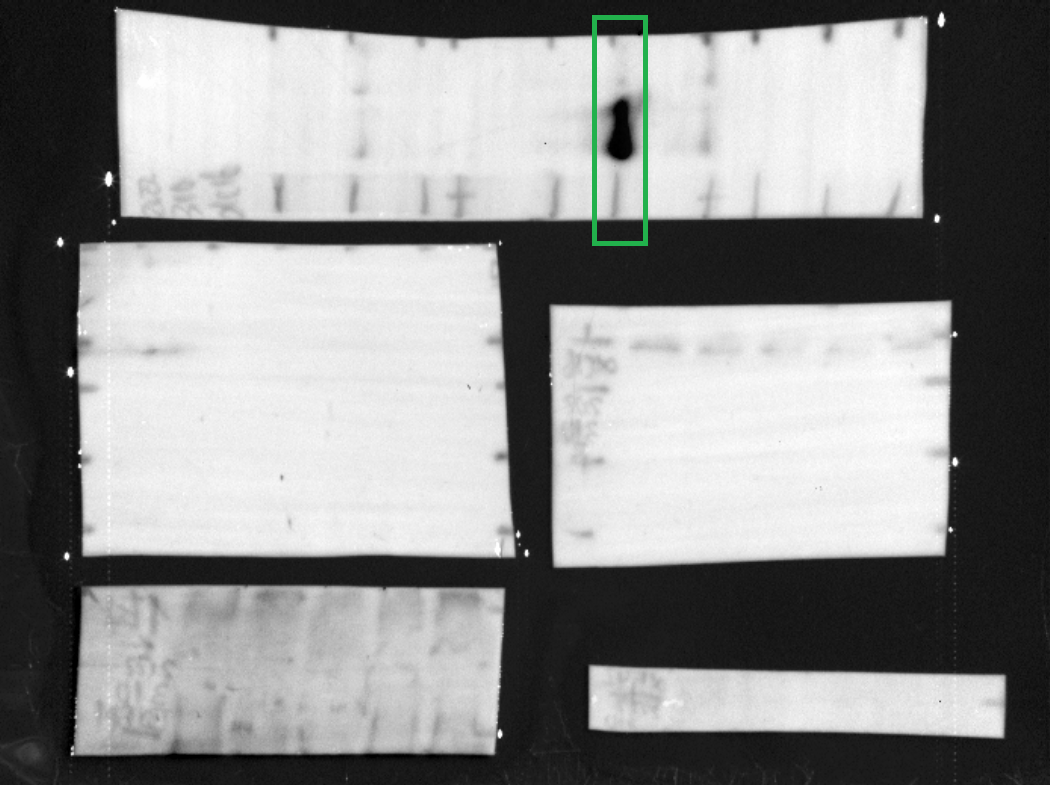

Supplement: Supplementary file 15 — Source data Fig. 6 [file 44320_2024_32_MOESM15_ESM.zip › Figure 6/Figure 6D/C16orf72-annotated.tif]

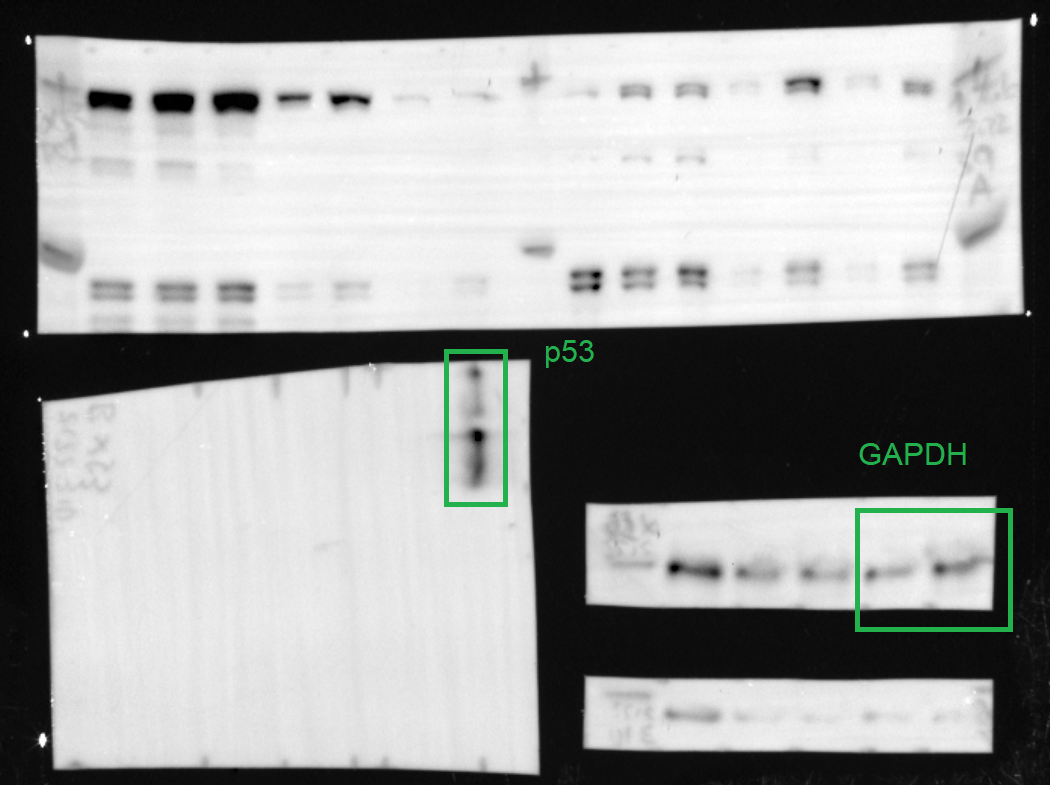

Supplement: Supplementary file 15 — Source data Fig. 6 [file 44320_2024_32_MOESM15_ESM.zip › Figure 6/Figure 6D/p53-GAPDH-annotated.png]

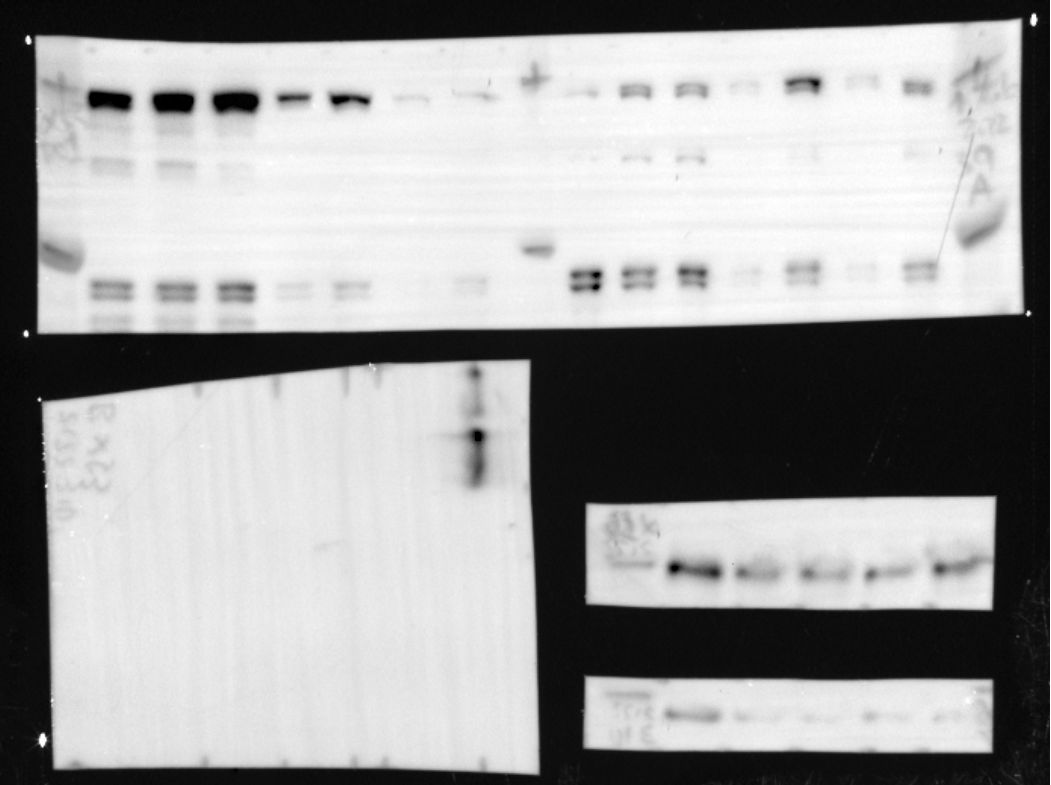

Supplement: Supplementary file 15 — Source data Fig. 6 [file 44320_2024_32_MOESM15_ESM.zip › Figure 6/Figure 6D/p53-GAPDH.tif]
